# Reconfigurable optical arithmetic logic unit and its applications

**DOI:** 10.1038/s41467-026-75750-x

**Published:** 2026-07-18

**Authors:** Xudong Zhou, Mingrui Yuan, Huifu Xiao, Yongheng Jiang, Pu Zhang, Yonghui Tian

**Affiliations:** 1 School of Physical Science and Technology, Lanzhou University, Lanzhou, China; 2 School of Mathematics and Physics, North China Electric Power University, Beijing, China

**Keywords:** Integrated optics, Silicon photonics

## Abstract

The growing constraints of Moore’s Law have fundamentally impeded further advancement in the energy efficiency of conventional electronic integrated circuits. In response, integrated photonics has emerged as a promising solution due to its exceptional bandwidth and ultralow latency. Optical digital computing based on integrated photonics offers inherent advantages in operation speed and energy efficiency compared to electronic digital schemes. However, implementing multi-functional arithmetic logic primitives on photonic chips still face a considerable challenge, and the reported works are mainly limited to basic logic gates or isolated computing modules rather than reconfigurable computational systems. Here, we report a reconfigurable optical computing architecture utilizing silicon-based microring modulators, which can implement reconfigurable basic logic operations and various arithmetic logic units, including 2-bit adder, subtractor and digital comparator. The experimental results demonstrate high computational density of 528 Gb/s/mm^2^ and energy efficiency of 12.36 fJ/bit at the operation speed of 20 Gb/s. The proposed device’s practical viability is further demonstrated through multiple application scenarios including data encryption and image processing.

## Abstract (web summary)

As silicon chips near their physical limits, light offers a faster alternative. The authors built and tested a silicon photonic chip whose tiny microring switches can be reprogrammed to add, subtract and compare 2-bit numbers at 20 Gb/s, and used it for encryption and medical-image processing.

## Introduction

The development of artificial intelligence (AI) is driving a significant increase in the demand for computing resources. However, this demand is increasingly at odds with the slowdown of Moore’s Law^1–3^, which has made it fundamentally challenging for traditional electronic integrated circuits to deliver the computational performance and energy efficiency. To overcome these limitations, integrated photonics serves as a promising solution. By leveraging light’s inherent advantages of parallel processing and large bandwidth, it offers a solution to break through the limitations of the current computational scheme and is poised to eventually replace part of the electronic circuits in future computing tasks^4–7^. As a cornerstone of digital computing, logic operations can also be implemented using integrated photonics, which show great potential to exceed electronic counterparts in speed, parallel processing capability, and energy efficiency^8–10^.

Over the past decade, significant progress has been made in on-chip optical digital computing, demonstrating the ability to perform logical operations using optical signals^11–14^. Current implementations are generally categorized into all-optical and electro-optical (E-O) approaches. By relying on nonlinear optical effects to achieve functionality, all-optical computing schemes face fundamental challenges, including restricted switching speeds, high power thresholds, and material-level constraints that impede large-scale integration^15–18^. In contrast, E-O computing schemes effectively combine the advantages of high-speed modulation and inherent optical parallelism^19,20^. Based on these advantages, E-O logic scheme utilizing integrated photonics is particularly suitable for current application scenarios and system-level integration. However, to the best of our knowledge, the reported works of E-O logic scheme have been primarily restricted to basic logic gates^21–27^, such as NOT, AND, OR, XOR, or single ALU such as one-bit adders, comparators, and subtractors^28–32^. Although recent works have demonstrated large-scale optical computing systems capable of implementing logic-related operations, significantly advancing the development of photonic logic computing, these approaches generally rely on large and complex network architectures, which differ from compact and reconfigurable ALU-oriented designs^33–37^. The absence of a compact reconfigurable architecture capable of implementing multiple complex logic functions continues to limit the development of fully functional photonic ALUs and practical optical processing systems.

In this work, we present a reconfigurable optical digital computing architecture using microring modulators (MRMs) on silicon-on-insulator (SOI) platform. Here, the MRMs are employed as efficient switching units for the proposed device to implement various logic operations due to its advantages such as compact footprint, low power consumption, and high modulation efficiency et al. Specifically, various basic logic operations can be achieved by cascading two MRMs with different switching states to form basic logic units (BLUs). These BLUs are further cascaded to realize ALUs. Using this proposed architecture, we successfully demonstrate BLUs, such as NOT, AND, OR, XOR, XNOR, as well as representative ALUs including 1-bit and 2-bit adders, subtractors, and comparators. Furthermore, we implement various applications based on the proposed device, such as information encryption, image watermark embedding, and difference amplification. The proposed device can achieve a computational density of 528 Gb/s/mm^2^ and an energy efficiency of 12.36 fJ/bit at the operation speed of 20 Gbps for ALUs, indicating its strong potential for high-performance photonic digital computing.

## Results

### Principle of this device

**Fig. 1:**
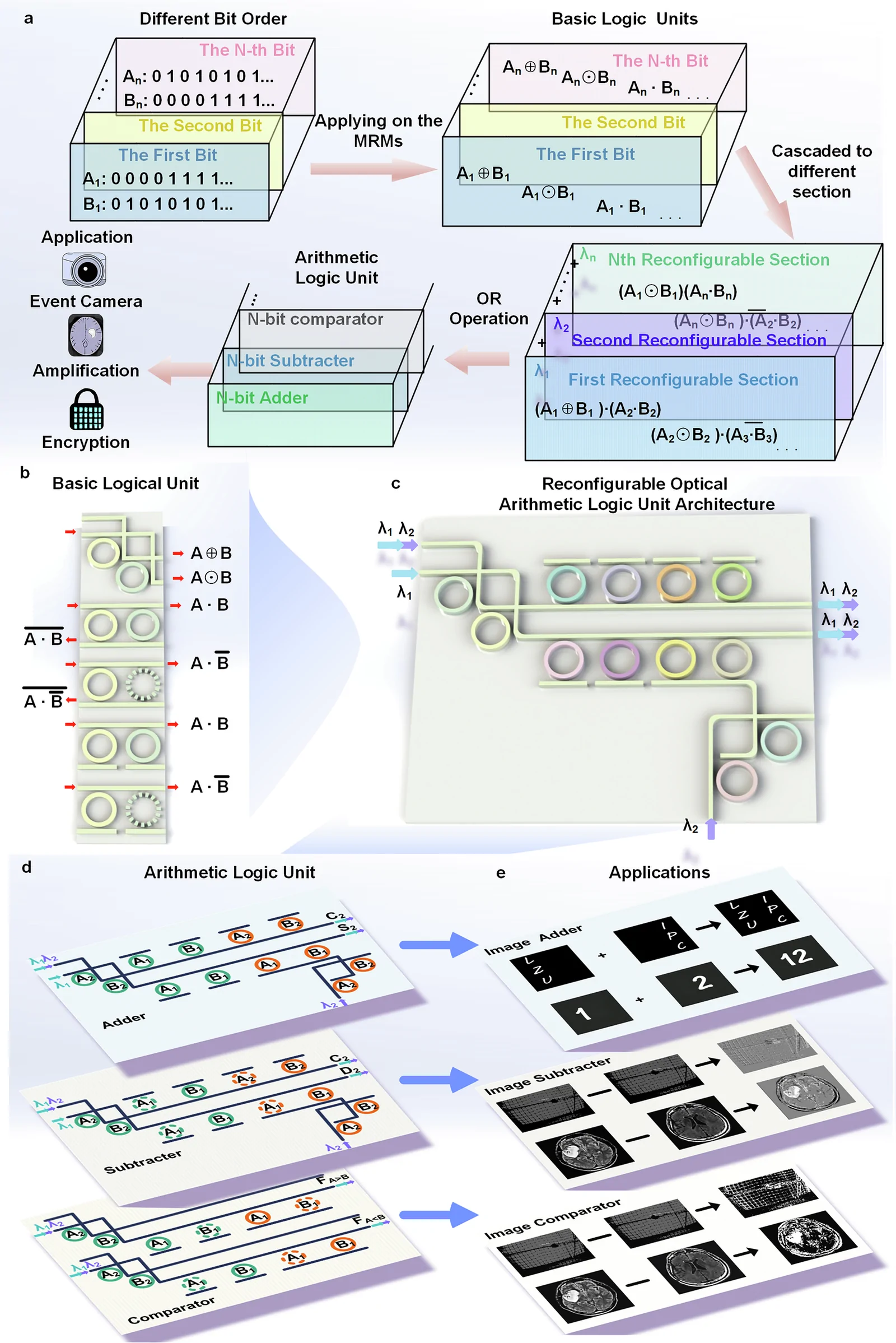
Schematic illustration of the proposed reconfigurable photonic computing architecture. **a** Conceptual diagram and functional schematic of this device. **b** Operational principle of BLU enabled by controlled state switching in MRM. **c** System architecture of the reconfigurable ALU platform. **d** Implementation scheme of 2-bit arithmetic units: adder, subtractor, and comparator. **e** Exemplary image processing applications enable by the 2-bit ALUs.

Figure 1a illustrates the operational principle of the proposed device, which processes two *n*-bit binary numbers, *B*_*n*_*B*_*n-1*_*…B*_*2*_*B*_*1*_ and *A*_*n*_*A*_*n-1*_*…A*_*2*_*A*_*1*_. In the electrical domain, it is easy to implement BLUs and cascade them to form more complex circuits. However, electrical systems lack inherent parallel processing capability, which prevents the efficient execution of OR operations following the cascading of BLUs^28^. Moreover, implementing reconfigurable BLUs in electrical systems remains highly complex due to the increasing circuit overhead and interconnection constraints. To overcome these limitations, we use E–O schemes in which electrical signals are applied to MRMs, with signals corresponding to different bit positions (e.g., *A*_*1*_*, A*_*2*_, *B*_*1*_, *B*_*2*_) addressed to distinct MRMs. By reconfiguring the cascading configuration of two MRMs driven by different input signals (for example, *A*_*1*_ and *B*_*1*_), the optical propagation paths are altered, thereby enabling the realization of different BLU functionalities within the same configuration. By further cascading these BLUs along programmable optical paths, reconfigurable circuit sections can be constructed. In addition, distinct functional regions are assigned to different optical wavelengths, allowing multiple OR operations to be executed simultaneously by exploiting the inherent parallelism of wavelength-division multiplexing. Complete ALUs are realized by altering the interconnection configurations among these sections. Since every processing stage is reconfigurable via electro-optic control, the overall function of the ALU can be dynamically tuned. For example, a ALU supporting operations such as addition, subtraction, and bitwise comparisons can be integrated on a single architecture. Here, we have applied this approach to implement a reconfigurable 2-bit ALU on-chip, thereby demonstrating its feasibility.

To achieve high-speed, compact, and reconfigurable optical digital computing, we utilize MRMs for efficient E-O signal conversion. These resonators can be electrically tuned with low voltage to switch between pass‑block and block‑pass states, enabling dynamic logic reconfiguration through operational state control (as shown in Supplementary Note 2). As depicted in Fig. 1b, the overall architecture is built from such discrete BLUs. Following the method in ref. ^38^, complementary XOR and XNOR operations are generated at separate output ports, while other basic units are realized by cascading modulators configured to different states. Through flexible combinations of these units, the system can perform a variety of complex computational functions.

Adders, subtractors, and comparators constitute the foundation of any ALU, respectively performing carry‑in addition, borrow‑in subtraction, and magnitude comparison. Their performance directly affects key system metrics such as processing capability, energy efficiency, and latency. Accordingly, we propose a generalized architecture capable of dynamically reconfiguring into any of these essential ALU modules. This design fully uses the advantages of MRMs, maintaining low power consumption while supporting parallel signal processing via wavelength‑division multiplexing, thus offering a paradigm for building high‑performance photonic computing systems.

For 1-bit logic computing, the truth table and corresponding Boolean expressions for the mentioned ALUs are summarized in Table 1. When operand sequences increase from one bit to two bits, electronic implementations require cascading multiple 1-bit ALUs in series; for instance, a 2-bit adder necessitates two 1-bit adders connected sequentially. This arrangement creates a sequential computational flow that severely limits processing efficiency, as each operation must await completion of the preceding one. In contrast, optical computing inherently supports parallel processing, enabling simultaneous calculations across different digit bits. While both electronic and optical approaches can implement these ALUs, only optical schemes fully exploit the inherent advantages of optical computing such as parallel processing and high-speed operation. Capitalizing on this advantage, we employ two distinct wavelength channels to represent 2-bit operands, allowing parallel implementation of 2-bit adders, subtractors, and comparators within our architecture. This wavelength-division multiplexing approach maintains the parallelism of optical computing while providing a scalable pathway for higher-bit operations.

**Table 1:** Truth table and logic expressions for the 1-bit optical ALU operations

Input		Adder		Subtractor		Comparator		
A_1_	B_1_	C_1_	S_1_	C_1+1_	D_1_	F_A>B_	F_A<B_	F_A=B_
0	0	0	0	0	0	0	0	1
0	1	0	1	1	1	0	1	0
1	0	0	1	0	1	1	0	0
1	1	1	0	0	0	0	0	1
Expression		A⋅B	A⊕B	$\overline{A}\cdot B$	A⊕B	$A\cdot \overline{B}$	$\overline{A}\cdot B$	A⊙B

For 2-bit ALUs, the corresponding simplified Boolean expressions are summarized in Table 2. It can be observed that each expression comprises one logical OR operation, combined with either an XOR or an XNOR operation, along with an AND operation. Due to the *A*⊕*B* operation can be simplified into $\overline{A}\cdot B+A\cdot\overline{ B}$. For 2-bit adder, the output can be simplified as^26^:

1 $${S}_{1}=	 {A}_{1}\oplus {B}_{1}\\ {S}_{2}=	 ({A}_{2}\oplus {B}_{2})(\overline{{A}_{1}\cdot {B}_{1}})+({A}_{2}\odot {B}_{2})({A}_{1}\cdot {B}_{1})\\ {C}_{out}=	 ({A}_{1}\cdot {B}_{1})({A}_{2}\oplus {B}_{2})+{A}_{2}\cdot {B}_{2}$$

**Table 2:** Logic expressions for the 2-bit optical ALU operations

Adder	C_i_	${C}_{i-1}({A}_{i}\oplus {B}_{i})+({A}_{i}\cdot {B}_{i})$
	S_i_	${A}_{i}\oplus {B}_{i}\oplus {C}_{i-1}$
Subtractor	C_i_	${C}_{i-1}(\overline{{A}_{i}\oplus {B}_{i}})+(\overline{{A}_{i}}\cdot {B}_{i})$
	D_i_	${A}_{i}\oplus {B}_{i}\oplus {C}_{i-1}$
Comparator	F_A>B_	${A}_{2}\cdot \overline{{B}_{2}}+({A}_{2}\odot {B}_{2})({A}_{1}\cdot \overline{{B}_{1}})$
	F_A<B_	$\overline{{A}_{2}}\cdot {B}_{2}+({A}_{2}\odot {B}_{2})(\overline{{A}_{1}}\cdot {B}_{1})$
	F_A=B_	$({A}_{1}\odot {B}_{1})\cdot({A}_{2}\odot {B}_{2})$

For 2-bit subtractor, the output can also be simplified as:

2 $${D}_{1}=	 {A}_{1}\oplus {B}_{1}\\ {D}_{2}=	 ({A}_{2}\oplus {B}_{2})(\overline{\overline{{A}_{1}}\cdot {B}_{1}})+({A}_{2}\odot {B}_{2})(\overline{{A}_{1}}\cdot {B}_{1})\\ {C}_{borrow\_out}=	 (\overline{{A}_{1}}\cdot {B}_{1})({A}_{2}\odot {B}_{2})+\overline{{A}_{2}}\cdot {B}_{2}$$

For 2-bit comparator, the output can also be simplified as:

3 $${F}_{A > B}=	 {A}_{2}\cdot \overline{{B}_{2}}+({A}_{2}\odot {B}_{2})({A}_{1}\cdot \overline{{B}_{1}})\\ {F}_{A < B}=	 \overline{{A}_{2}}\cdot {B}_{2}+({A}_{2}\odot {B}_{2})(\overline{{A}_{1}}\cdot {B}_{1})\\ {F}_{A=B}=	 ({A}_{1}\odot {B}_{1})\cdot({A}_{2}\odot {B}_{2})$$

Accordingly, we propose the architecture illustrated in Fig. 1c to realize reconfigurable ALUs using two XOR/XNOR units and two additional BLUs. By controlling the state of the MRMs, different logical operations can be performed. Furthermore, through wavelength-selective input signals, distinct microrings can be activated to process different digits concurrently. This approach enables the effective implementation of 2-bit ALUs within the presented architecture. Here, Fig. 1d shows the details of the different states of the microrings in different ALUs architecture, where the solid ring is the pass/block ring and the dotted-line ring is the block/pass ring (as shown in Supplementary Note 2). Besides, the different colors mean the modulator interacts with different wavelengths. By using this architecture, different outputs of ALUs can be received at different ports, which can support reconfigurable functions.

### Fabrication of the device

**Fig. 2:**
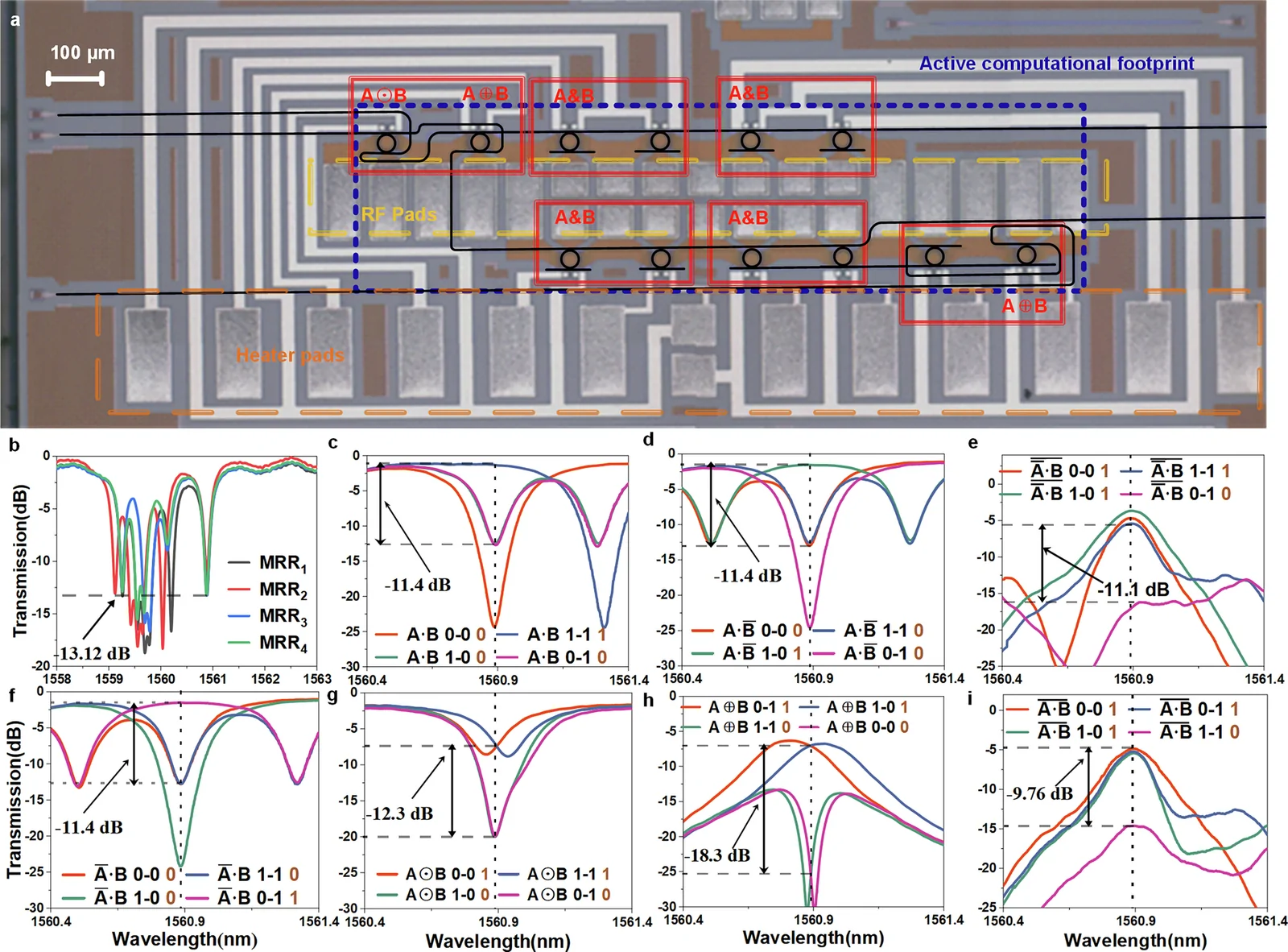
Architectural micrographs and basic logic operation characterization. **a** The microscope image of the proposed device with different region. **b** The different microrings resonator at same wavelength. Static spectral response of different fundamental optical operations **c** A⋅B **d** $A\cdot \overline{B}$ **e** $\overline{\overline{A}\cdot B}$ **f** $\overline{A}\cdot B$ **g** A⊙B **h** A⊕B **i** $\overline{A\cdot B}$.

The proposed photonic computing device is fabricated on a SOI platform comprising a 220-nm-thick top silicon layer and a 2-μm buried oxide layer, with a complete device micrograph shown in Fig. 2a. And in Fig. 2a, we have additionally added red boxes to indicate the units performing different BLUs, black lines to represent the waveguide routing, pads within orange boxes for applying thermal signals, and yellow boxes to denote the pads for high-speed RF signals. Here, we use a rib waveguide with a rib height of 150 nm and a slab of 70 nm to construct the proposed device. To achieve optimal optical performance and manufacturing viability, the radii of waveguide bends are designed to be 10-μm radii to minimize bending losses while maintaining efficient light confinement, alongside a layout that reduces electrical signal attenuation while ensuring precise alignment with corresponding contact pads. Each microring resonator implements a dual-modulation scheme, integrating both a microheater for thermal resonance wavelength tuning and a PN junction operating on the carrier-depletion principle for high-speed electro-optic modulation; the performance characterization of the MRM is presented in Supplementary Note 3. This hybrid configuration enables independent control of operating wavelength and dynamic signal modulation, significantly enhancing logical function reconfigurability. The co-integration of these complementary tuning methods within individual microrings improves functional versatility while maintaining a compact footprint. Fabrication utilized standard 180-nm CMOS-compatible processes, with 70-nm shallow etching for the grating coupler. This integrated approach establishes a solid foundation for developing scalable, reconfigurable optical computing systems that bridge conventional electronic processing and E-O computing paradigms, with the successful device implementation validating the feasibility of complex photonic systems for practical computing applications.

### Demonstration of device’s functions

To experimentally validate the design consistency and fabrication stability of the photonic architecture, we characterized the static transmission spectra of four different microring resonators. Figure 2b shows that all resonators exhibit nearly identical spectral responses, with extinction ratios consistently around −13.12 dB. This uniformity confirms high fabrication reproducibility of the fabricated device. The output spectra for various logic operations are presented in Fig. 2c–i. These results demonstrate the successful implementation of all basic logic functions, including AND, XOR, XNOR, etc., using only two microring resonators in the proposed architecture. The dynamic range between the optical power levels representing logic high and low states varies across different logic units. The measured extinction ratios range from −9.76 to −18.3 dB. The high extinction ratios and uniform resonator performance confirm that the basic logic operations provide sufficient signal integrity for reliable cascading in more complex arithmetic logic units. These experimental results collectively validate the fabrication stability and the functional reliability of the designed photonic logic units. The architectural approach supports reconfigurable and compact optical computing with clear potential for scalable integration.

**Fig. 3:**
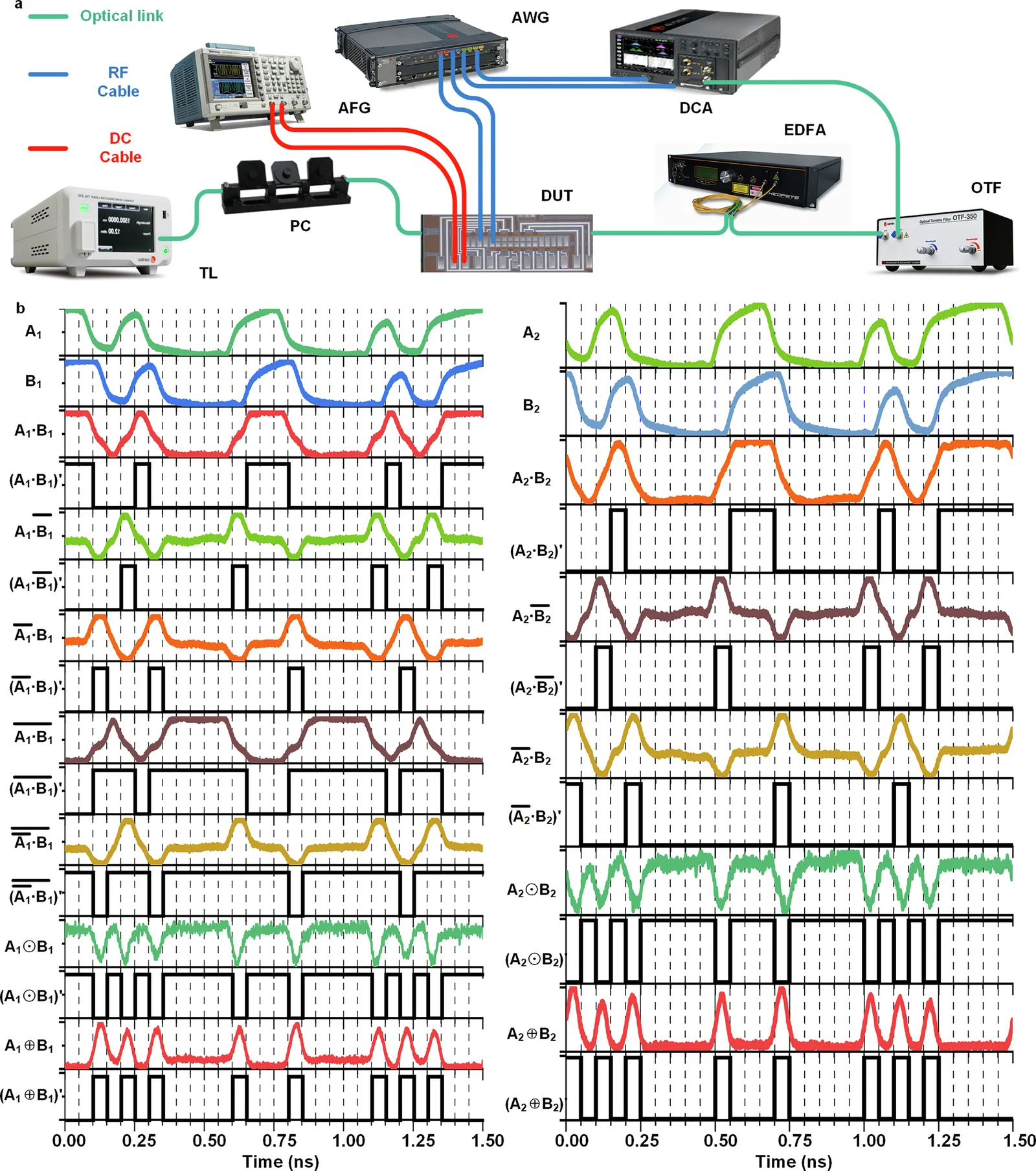
Experimental demonstration of the BLUs at 20 Gbit/s. **a** Experimental setup for characterizing BLUs of the proposed device. **b** Experimental results of basic logical operation at 20 Gbit/s and results after DSP with marked by apostrophes.

Following the static performance evaluation, we conduct high-speed dynamic tests to assess the logic operation performance of the device under realistic operating conditions. Figure 3a illustrates the complete experimental setup. A tunable laser generated the continuous-wave optical signal, which passed through a polarization controller to align its polarization state with the input grating coupler before being coupled into the device under test (DUT). The output optical signal from the DUT is amplified by an erbium-doped fiber amplifier (EDFA) and then captured and analyzed by a digital communications analyzer (DCA). On the electrical driving side, two independent radio frequency signals generated by an arbitrary waveform generator (AWG) are applied to the DUT electrodes via high-speed probes. Direct-current bias voltages are introduced through the same probe setup. A synchronization clock signal from the AWG triggered the DCA to ensure precise time-aligned acquisition of the output waveforms. Due to the AWG hardware limitation of providing only two synchronized electrical outputs simultaneously, all basic logic operations are characterized using this two-signal configuration. For logic functions requiring an OR operation between two BLUs, this gate is implemented in the electrical domain. Lower-speed modulation tests confirmed that this electrical-domain processing produced results fully consistent with a full optical implementation, as documented in the Supplementary Notes 4 and 5.

The dynamic test utilized a 20 Gbit/s with a length of 30 bit as the electrical input. The corresponding output optical signal is converted by a photodetector and recorded by the DCA. The initially captured raw waveform, summarized in Fig. 3b, shows distorted logic states due to device response limitations and system noise. To recover the valid computational data, we use a custom digital signal processing (DSP) algorithm. This algorithm performs adaptive threshold decision and precise timing alignment on the recorded waveforms. Its implementation details are provided in the Supplementary Note 6. The processing effectively suppressed inter-symbol interference and clarified the logic levels. The final processed results, indicated by apostrophes in Fig. 3b, now clearly demonstrate correct logic functionality at 20 Gbit/s for all target operations. These results confirm that the microring-based logic units can perform reliable high-speed computations and are suitable as core components for constructing practical optical arithmetic logic units.

**Fig. 4:**
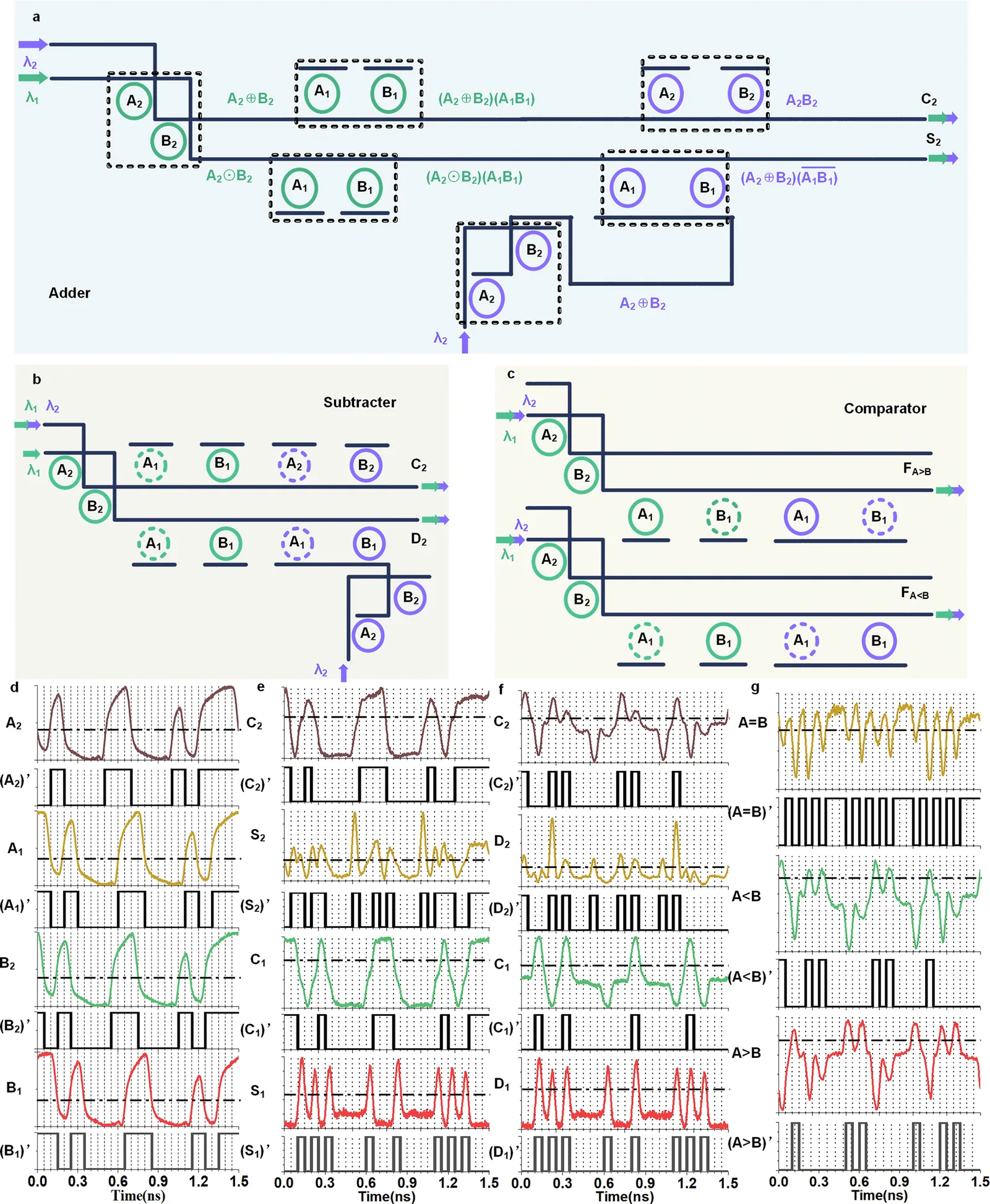
Experimental results of ALUs at 20 Gbit/s. **a** The architecture for reconstructing adder with details. **b** The architecture for reconstructing subtractor. **c** The architecture for reconstructing comparator. **d** The pseudo-random code signal. **e**–**g** The experimental results of 2-bit adder, 2-bit subtractors and 2-bit comparator, respectively, and results after DSP with marked by apostrophes.

To demonstrate the functional reconfigurability of the proposed device, we have implemented fundamental 2-bit arithmetic operations, including an adder, a subtractor, and a comparator. Using the 2-bit adder as a primary example, Fig. 4a outlines its detailed implementation process. The output of the adder consists of three distinct signals as defined by Eq. (1): the first-order sum bit *S*_*1*_, the second-order sum bit *S*_*2*_, and the output carry bit *C*_*2*_. Based on the derived logical expressions, *S*_*1*_ is produced directly by an AND logic unit when the input signals *A*_*1*_ and *B*_*1*_ are applied to the corresponding microring resonators. The computation of *S*_*2*_ is separated into two independent wavelength-specific components. The first component corresponds to the logical expression $({A}_{2}\odot {B}_{2})({A}_{1}\cdot {B}_{1})$ and is processed by a microring modulator tuned to wavelength λ_1_. The second component corresponds to the logical expression $({{A}_{2}} \oplus {{B}_{2}}) (\overline{{{A}_{1}} \cdot {{B}_{1}}})$ and is handled simultaneously by a second modulator tuned to wavelength λ_2_. The intrinsic parallelism of optical signal transmission allows both data streams to be carried and processed concurrently. This capability permits the output from one BLU to feed directly into the input of a subsequent unit, supporting necessary OR operations and the combination of sequential logic stages. Additionally, optical signals at different wavelengths operate without mutual interference, enabling the two independent components for *S*_*2*_ to be combined through direct optical summation at the output port, yielding the final S_2_ result. The output carry bit *C*_*2*_ is generated using an identical computational approach, applying the same principles of wavelength-division multiplexing. This same reconfigurable architecture also supports the implementation of a 2-bit subtractor and a comparator. By adjusting the operational states of the microring modulators, the device can switch between these different arithmetic functions without physical redesign.

The detailed architectures for the 2-bit subtractor and comparator are provided in Fig. 4b, c, respectively. Each ALU is reconfigured by electrically switching the operational states of MRMs. As defined in the basic logic operation section, a solid-ring symbol denotes a resonator in the pass/block state, while a dotted-line ring indicates the block/pass state. By applying this method, different input signals are directed to specific microrings to generate the required elementary logic outputs. Subsequent OR logic operations necessary for the final result are then performed in the electrical domain, completing the overall computation. The experimental results for all three ALU functions are summarized in Fig. 4d–f. A 20 Gbit/s pseudo-random bit sequence is used as the input signal to test each reconfigured ALU. In the figures, results without DSP are marked as their digit with a different color, and the same DSP algorithm employed for the BLU characterization is applied to recover clear logic states from the measured output waveforms. All processed results show correct logical functionality for the adder, subtractor, and comparator operations, which align perfectly with the expected truth tables. This confirms the successful reconfiguration of the same hardware platform into multiple, fully functional arithmetic units, validating the practicality and versatility of the proposed wavelength-parallel design for photonic computing.

### Application of this device

#### Encryption and decryption processing using XOR unit

The XOR operation serves as an ideal cryptographic primitive due to its fundamental role in symmetric-key cryptography: when a plaintext bit undergoes XOR operation with a key bit, the resulting ciphertext can be perfectly decrypted through a second XOR operation with the identical key^39^. This bijective property, combined with the inherent resilience of photonic implementations against side-channel attacks, establishes a robust foundation for encryption systems.

**Fig. 5:**
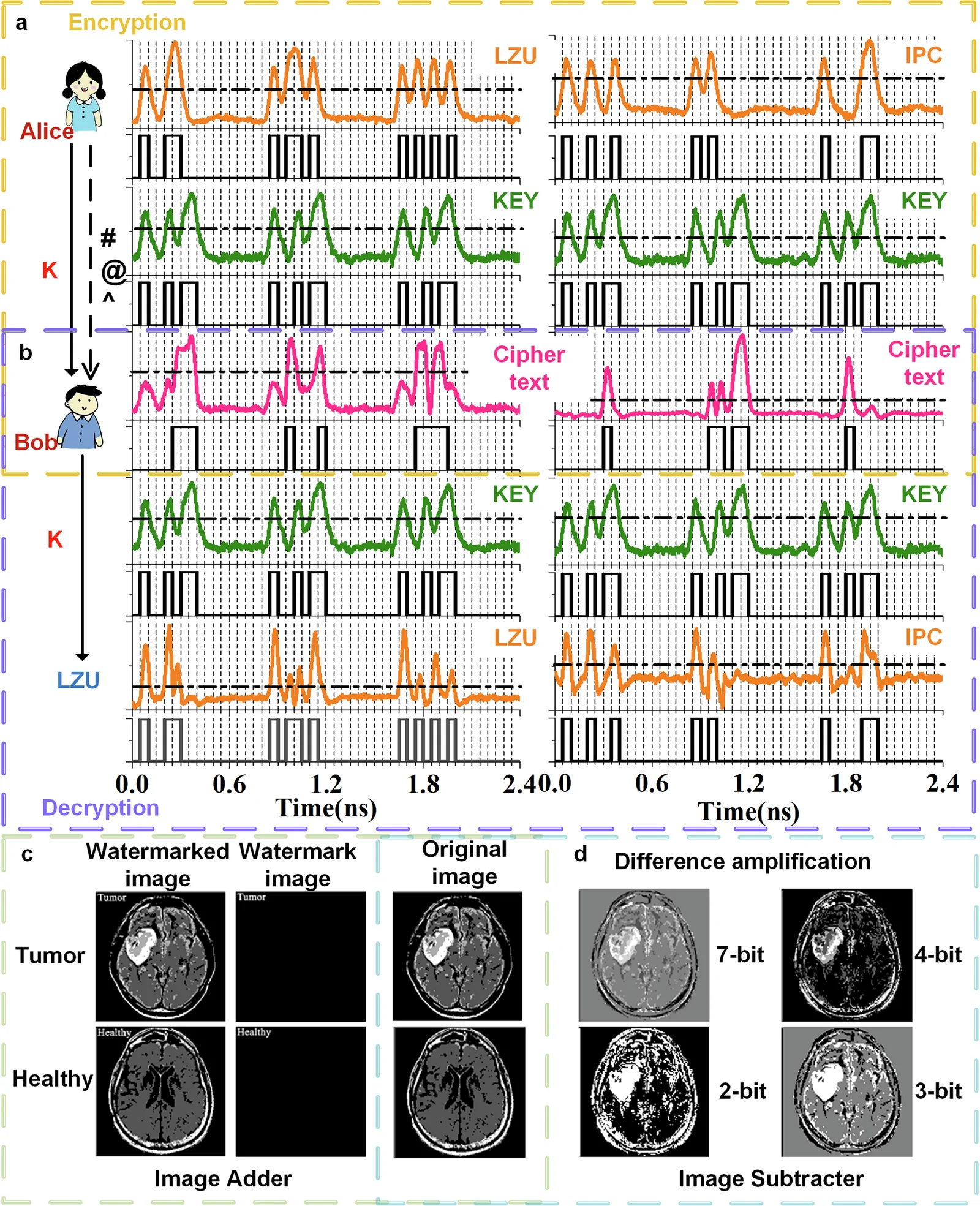
Experimental results of applications. **a** The experimental results with and without DSP of encryption processing. **b** The experimental results with and without DSP of decrypt processing. **c** The experimental result of image adder for watermark adding application. **d** The experimental result of the image subtracter for the differential amplification application.

In this work, we implement an encryption system based on the proposed architecture by utilizing two MRMs to execute the XOR operation for cryptographic processing. The system is configured with the first MRM carrying the plaintext data stream and the second MRM carrying an encryption key stream. The optical XOR logic performed between these two streams directly generates the corresponding ciphertext at the output. For experimental validation, we encoded representative text strings into 20 Gbit/s electrical signals using ASCII format. The plaintext messages “L Z U” and “I P C” are encrypted with a repeating key sequence “K K K”. These electrical signals are then applied to their respective MRMs. Both modulators are operated at the same optical wavelength and are precisely synchronized to ensure correct bit-level temporal alignment during the XOR process. The output optical signal is converted to the electrical domain and processed using the mentioned DSP methodology outlined earlier. The processed experimental results with and without DSP show the obtained ciphertext, which is presented in Fig. 5a and matches the theoretically expected output exactly. Subsequently, decryption is performed by re-applying the same key sequence to the ciphertext in a second, identical XOR operation using the same photonic hardware configuration. This process successfully recovered the original plaintext in its entirety without any errors, as shown in Fig. 5b. This successful encryption and decryption cycle validates the practical functionality of the photonic XOR unit and demonstrates its direct applicability as a core for high-speed components for secure optical communication systems.

This cryptographic implementation exemplifies the architecture’s remarkable functional flexibility. While we have demonstrated the XOR operation’s effectiveness in encryption applications, the same hardware platform can be reconfigured to utilize other basic logic operations for different computational tasks. Such reconfigurability establishes our architecture as a truly versatile computing platform capable of addressing diverse processing requirements through programmable control of its fundamental logic elements, highlighting its adaptability across various application scenarios.

#### Image watermarking with adder

The implemented ALUs demonstrate significant practical utility in application scenarios that exploit the inherent advantages of optical digital computing. To illustrate this capability, we employ medical image watermarking as a representative case utilizing the proposed 2-bit adder architecture. This application leverages the four distinct states encodable in a 2-bit binary signal to embed critical diagnostic information directly into medical images.

As shown in Fig. 5c, we utilize Magnetic Resonance Imaging (MRI) scans, the original image, as 128 × 128 base images^40^, with the upper image designated for “Tumor” watermarking and the lower for “Healthy” watermarking, creating a visually intuitive demonstration of the watermarking capability while maintaining diagnostic relevance. The watermarking process employs a streamlined technical approach where both the MRI base images and diagnostic markers undergo identical four-level grayscale conversion, with each intensity level mapped to corresponding 2-bit binary representations. The 2-bit adder architecture then performs parallel pixel-wise addition between the encoded data streams, achieving 100% calculation accuracy throughout the watermarking pipeline. This operational reliability demonstrates the system’s robustness for adder applications.

#### Image difference amplification with subtractor

The 2-bit subtractor architecture demonstrates versatility in image processing applications, particularly in difference amplification. Building upon the previously established watermarking demonstration, we employ the same MRI image to implement image difference amplification, designed to enhance contrast discrimination between healthy and pathological tissue regions. This application capitalizes on the intrinsic capability of optical computing to perform parallel arithmetic operations across entire image frames simultaneously, enabling real-time enhancement of subtle anatomical variations that may escape conventional visual inspection. The differential processing methodology proves particularly valuable in medical diagnostics where minute morphological differences often carry critical diagnostic significance. In the implementation, input images undergo identical encoding procedures as established in the watermarking workflow, with pixel intensities mapped to corresponding 2-bit binary representations before being applied to synchronized MRMs within the subtractor configuration. A feature of this approach lies in the 2-bit subtractor’s output range, which spans seven discrete intensity levels corresponding to decimal values from −3 to +3. This expanded dynamic range enables the reconstruction of a different-level grayscale image that preserves polarity of inter-image differences, as shown in Fig. 5d. The resulting differential images provide significantly enhanced visualization of pathological features, with the negative-to-positive value continuum allowing clear differentiation between various tissue characteristics.

The implementation validates not only the practical effectiveness of our optical computing approach but also highlights its potential for automated image processing systems, combining the inherent advantages of optical computing, including low latency and high parallelism, with the rigorous accuracy requirements of practical applications.

## Discussion

The proposed architecture employs MRMs to implement digital logic computing, offering significant advantages in integration density, power efficiency, and modulation performance. To balance integration scale with modulation capability, the microring resonators are designed with a radius of 20 μm, substantially more compact than conventional MZI-based modulators. The reported device dimensions of 1.42 × 0.32 mm² correspond to the active computational footprint, which includes only the MRMs and their adjacent functional waveguides directly involved in logic operations. The larger layout shown in Fig. 2 additionally contains passive routing waveguides, optical access ports, and electrical probe-pad structures required for experimental characterization, and these components are not included in the computational density evaluation. The current experimental implementation is primarily constrained by the 100 μm pitch requirement for high-frequency electrical probes and the 200 μm pitch requirement for thermo-optic tuning probes. With optimized optoelectronic co-packaging and electrical routing, the overall footprint could be further reduced to 0.66 × 0.25 mm². While functional verification has been conducted at 20 Gbit/s, this data rate represents neither the fundamental limit of the MRMs nor the maximum potential of the architecture, as the modulators have been demonstrated to support operation up to 80 Gbit/s in separate characterization. This higher modulation speed establishes the practical performance boundary for the current architectural implementation while highlighting substantial headroom for future development.

The proposed architecture requires only two MRMs to perform fundamental logical operations, enabling simultaneous execution of six basic logic functions. This configuration yields an aggregate computational throughput of 120 Gbit/s in the current implementation, with a theoretical maximum of 480 Gbit/s achievable under optimized conditions. The specific throughput varies across different ALUs according to their respective operational complexities: adders and subtractors each operate at 20 Gbit/s, while comparators achieve 40 Gbit/s. These operational rates represent current demonstrated performance, with theoretical capabilities projecting a fourfold increase potential. When evaluated for computational density, the architecture presently achieves 528 Gbit/s/mm^2^, with a prospective density of 5.8 Tbit/s/mm^2^ attainable through further development. This density metric substantially surpasses those achieved by alternative architectures employing MZI modulators, effectively validating the superior integration density and high-speed performance inherent to the MRM design paradigm.

In addition to the compact physical dimensions, the MRMs employed in this architecture demonstrate superior power efficiency compared to conventional MZI modulators. The system utilizes twelve MRMs for electrical signal loading, substantially reducing overall power consumption. With per-bit energy consumption estimated at 1.03 fJ/bit for individual MRMs^33^, the power requirements for different computational operations can be precisely quantified. Each basic logic operation, utilizing two MRMs, consumes 0.0412 mW at the operating speed of 20 Gbit/s. For the complete set of ALUs, the aggregate power consumption reaches 0.247 mW under the same operating conditions. Particularly noteworthy is the image difference amplification process, which requires only 2.43 × 10^−7 ^mW during computation. These remarkably low power figures, coupled with the previously demonstrated compact footprint, effectively validate the MRM-based architecture as an energy-efficient solution for high-speed photonic computing applications, achieving significant advantages in both spatial efficiency and power consumption relative to alternative modulator technologies. A detailed performance comparison against ohter E-O counterparts is provided in Table 3.

**Table 3:** Performance comparation against electro-optic counterparts

Work	OU.	BLu	ALU	R.f.	Fre. [GHz]	CD [Gbit/s/mm^2^]	PC	Applications
[20]	MZI	6	/	Yes	32	/	/	/
[22]	MRR	/	3	Yes	/	/	/	/
[28]	MZI	2	1	No	20	20	3.9 fJ/bit	/
[32]	MZI	>5	/	Yes	25	200	0.32 pJ/bit	3^a^
This work	MRM	>7	3	Yes	20	528	12.36 fJ/bit	3^b^

*OU* operation unit, *R.f.* reconfigurable, *Fre.* frequency, *CD* computing density, *PC* power consumption.

^a^Elementary cellular automata, encryption and decryption, edge extraction (XOR).

^b^Encryption and decryption, image adder, image subtractor.

The proposed architecture demonstrates significant potential for reconfigurable basic logic operations and ALUs through the innovative implementation of MRMs, achieving notably lower power consumption alongside higher computational density. To further advance the performance of this platform, several promising development paths emerge. First, the modulation rate could be enhanced through optimized design of the PN junction or by transitioning to alternative platforms featuring superior electro-optic coefficients, such as lithium niobate on insulator (LNOI), lead zirconate titanate (PZT), or barium titanate (BTO). Such improvements would leverage higher electro-optic modulation speeds to elevate computational density while maintaining strict power constraints. Second, while the current device successfully implements reconfigurable 2-bit adders, subtractors, and comparators by cascaded BLUs, we have validated a time-domain iterative computation strategy to enable multi-bit ALU operation without proportional hardware scaling, with full scalability analysis and mitigation strategies for large-scale integration challenges detailed in Supplementary Note 9. Future work could further refine this cascading and iterative processing methodology to support more diverse and computationally complex applications without sacrificing reconfigurability or energy efficiency. Third, adopting co-packaged optics technology presents a compelling route to further increase device integration density. This approach would enable the replacement of conventional electronic chips with photonic counterparts, delivering higher computing speeds at reduced power consumption.

In conclusion, we demonstrate a reconfigurable optical digital computing architecture based on MRMs on a SOI platform, designed to overcome the growing limitations of electronic circuits in terms of energy efficiency and computational throughput. The architecture enables dynamic implementation of basic logic operations (NOT, AND, OR, XOR, XNOR) and complex ALUs, including 2-bit adders, subtractors, and comparators. Functioning through low-voltage tuning between block/pass and pass/block states, the MRMs provide complementary outputs and support wavelength-division multiplexing, which facilitates parallel signal processing. Experimental validation confirmed successful operation at 20 Gb/s, with the MRMs themselves supporting up to 80 Gb/s, highlighting significant potential for speed scaling. The architecture achieves a computational density of 528 Gb/s/mm^2^ and an ultra-low power consumption of 12.36 fJ/bit, substantially outperforming MZI-based alternatives. Practical functionality is further verified through applications such as optical encryption, image watermarking, and differential image enhancement, underscoring the versatility and applicability of this platform for future high-speed, energy-efficient photonic computing systems.

## Methods

### Simulation and fabrication of the proposed architecture

The static transmission characteristics of the proposed architecture are simulated using the finite-difference time-domain method, with particular focus on the coupling ratio between the microring resonators and strip waveguides. Additionally, the modulation performance is evaluated through numerical simulations. The key geometrical parameters of the device are specified as follows. The waveguide structure features a uniform width of 0.4 μm and a bending radius of 10 μm to ensure efficient light confinement and low-loss propagation. The microring resonators are designed in a racetrack configuration with a bending radius of 20 μm, a straight racetrack section of 2 μm, and a coupling gap of 0.47 μm between the ring and the adjacent waveguide. These dimensions are optimized to achieve critical coupling conditions while maintaining fabrication feasibility within standard silicon photonics processes.

The device is fabricated through a multi-project wafer process to ensure reproducibility and scalability. For the thermal tuning components, titanium nitride (TiN) microheaters are implemented with aluminum interconnects, providing stable and efficient thermo-optic control of the microring resonators. The high-speed electro-optic modulation section employs a reverse-biased PN junction design utilizing a combined heavy-and-light doping profile to optimize both modulation efficiency and optical loss. Optical input and output are facilitated by grating couplers, which enable efficient vertical light coupling while maintaining compatibility with standard fiber-array packaging techniques. This integrated fabrication approach successfully combines thermal and carrier-depletion tuning mechanisms within a unified silicon photonics platform, ensuring robust performance across both static and dynamic operating conditions.

### Experimental setup

The experimental setup, schematically illustrated in Fig. 3a, incorporates commercially available instruments to ensure reliable characterization. In the optical path, a Santec TSL-570 tunable laser source provides the input optical signal, followed by polarization state adjustment using a Thorlabs FPC561 polarization controller to optimize coupling efficiency with the grating couplers. A KEOPSYS EDFA boosts the optical signal, with a Santec OTF-350 tunable optical filter subsequently removing amplified spontaneous emission noise. For electrical control, a reverse bias voltage is applied to the microring modulators using a DC source, while high-speed electrical signals are generated by a Keysight M8199B AWG operating at 224 GSa/s with dual output channels. The resulting optical waveforms are captured and analyzed using a Keysight N1000A digital communication analyzer, completing a comprehensive measurement system capable of both static and dynamic device characterization.

## Supplementary information

Supplementary Information

Supplementary Dataset 1

Transparent Peer Review file

## Data Availability

All the data supporting the findings in this study are available in the paper and Supplementary Information. Additional data related to this paper are available from the corresponding authors upon request.

## References

[1] Waldrop, M. M. The chips are down for Moore’s law. *Nat. News* **530**, 144 (2016). 10.1038/530144a 26863965

[2] Lundstrom, M. S. & Alam, M. A. Moore’s law: The journey ahead. *Science* **378**, 722–723 (2022). 36395227 10.1126/science.ade2191

[3] Waldrop, M. M. More than Moore. *Nature* **530**, 144–148 (2016). 26863965 10.1038/530144a

[4] Li, C., Zhang, X., Li, J., Fang, T. & Dong, X. The challenges of modern computing and new opportunities for optics. *PhotoniX* **2**, 20 (2021).

[5] McMahon, P. L. The physics of optical computing. *Nat. Rev. Phys.* **5**, 717–734 (2023).

[6] Bente, I. et al. The potential of multidimensional photonic computing. *Nat. Rev. Phys.* **7**, 439–450 (2025).

[7] Ahmed, S. R. et al. Universal photonic artificial intelligence acceleration. *Nature* **640**, 368–374 (2025). 40205212 10.1038/s41586-025-08854-x

[8] Fang, L. Optical logic array: a photonic solution towards universal computing. *Front. Optoelectron.* **17**, 1–2 (2024). 39719501 10.1007/s12200-024-00145-z PMC11668712

[9] Caulfield, H. J. & Dolev, S. Why future supercomputing requires optics. *Nat. Photonics* **4**, 261–263 (2010).

[10] Cotter, D. et al. Nonlinear optics for high-speed digital information processing. *Science* **286**, 1523–1528 (1999). 10567251 10.1126/science.286.5444.1523

[11] Jiao, S. et al. All-optical logic gate computing for high-speed parallel information processing. *Opto-Electron. Sci.* **1**, 220010 (2022).

[12] Song, X. et al. Multifunctional logic gates via coexistent positive and negative photoconductivity of a single Bi2Se3 photodetector. *Adv. Mater*. **37**, 2416934 (2025). 10.1002/adma.202416934 40345973

[13] Kim, W. et al. From light to logic: Recent advances in optoelectronic logic gate. *Small Sci* **4**, 2400264 (2024). 40213488 10.1002/smsc.202400264 PMC11935027

[14] Ge, Y. et al. All-optical logic processing unit using Kerr nonlinearity of MXene. *Nat. Commun*. **17**, 4078 (2026). 10.1038/s41467-026-70834-0 PMC13144690 41844655

[15] Xu, J. et al. All-in-one, all-optical logic gates using liquid metal plasmon nonlinearity. *Nat. Commun.* **15**, 1726 (2024). 38409174 10.1038/s41467-024-46014-3 PMC10897469

[16] Ding, X. et al. Metasurface-based optical logic operators driven by diffractive neural networks. *Adv. Mater.* **36**, 2308993 (2024). 10.1002/adma.202308993 38032696

[17] Qian, C. et al. Performing optical logic operations by a diffractive neural network. *Light Sci. Appl.* **9**, 59 (2020). 32337023 10.1038/s41377-020-0303-2 PMC7154031

[18] Cong, G. et al. On-chip bacterial foraging training in silicon photonic circuits for projection-enabled nonlinear classification. *Nat. Commun.* **13**, 3261 (2022). 35773261 10.1038/s41467-022-30906-3 PMC9247170

[19] Qiu, C., Xiao, H., Wang, L. & Tian, Y. Recent advances in integrated optical directed logic operations for high performance optical computing: a review. *Front. Optoelectron.* **15**, 1 (2022). 36637553 10.1007/s12200-022-00001-y PMC9756239

[20] Gostimirovic, D. & Ye, W. N. Ultracompact CMOS-compatible optical logic using carrier depletion in microdisk resonators. *Sci. Rep.* **7**, 12603 (2017). 28974692 10.1038/s41598-017-12680-1 PMC5626730

[21] Shekhar, S. et al. Roadmapping the next generation of silicon photonics. *Nat. Commun.* **15**, 751 (2024). 38272873 10.1038/s41467-024-44750-0 PMC10811194

[22] Ghosh, R. R. & Dhawan, A. Low loss, broadband, and non-volatile ‘directed logic operations’ using phase change materials in silicon photonics. *IEEE J. Quantum Electron.* **59**, 1–13 (2023).

[23] Wang, W. et al. Optically-reconfigurable integrated optical directed logic computing based on silicon photonics. *Opt. Express* **32**, 24007–24017 (2024). 39538852 10.1364/OE.523683

[24] Xia, Y. et al. A multifunctional optical computing system based on directed logic and micro-ring resonator. *Opt. Laser Technol.* **175**, 110696 (2024).

[25] Li, F. et al. Bit-scalable optical logic gates based on directed logic and micro-ring resonators. *Opt. Commun*. **587**, 131901 (2025).

[26] Hou, J., Chen, L., Dong, W. & Zhang, X. 40 Gb/s reconfigurable optical logic gates based on FWM in silicon waveguide. *Opt. Express* **24**, 2701–2711 (2016). 26906841 10.1364/OE.24.002701

[27] Soref, R., De Leonardis, F., Ying, Z., Passaro, V. M. & Chen, R. T. Silicon-based group-IV OEO devices for gain, logic, and wavelength conversion. *ACS Photonics* **7**, 800–811 (2020).

[28] Xiao, H. et al. Demonstration of various optical directed logic operations by using an integrated photonic circuit. *Opt. Lett.* **46**, 2457–2460 (2021). 33988609 10.1364/OL.423858

[29] Ruhul Fatin, M., Gostimirovic, D. & Ye, W. N. Reconfigurable optical logic in silicon platform. *Sci. Rep.* **14**, 5950 (2024). 38467741 10.1038/s41598-024-56463-x PMC10928202

[30] Ying, Z. et al. Electronic-photonic arithmetic logic unit for high-speed computing. *Nat. Commun.* **11**, 2154 (2020). 32358492 10.1038/s41467-020-16057-3 PMC7195421

[31] Feng, C. et al. Toward high-speed and energy-efficient computing: a WDM-based scalable on-chip silicon integrated optical comparator. *Laser Photonics Rev.* **15**, 2000275 (2021).

[32] Zhang, W. et al. Time-space multiplexed photonic-electronic digital multiplier. *Photonics Res.* **12**, 499–504 (2024).

[33] Zhang, W. et al. Photonic logic tensor computing beyond Tbit/s per core. *Optica* **12**, 1252–1260 (2025).

[34] Zhang, W. et al. Large-scale optical programmable logic array for two-dimensional cellular automaton. *Adv. Photonics* **6**, 056007 (2024).

[35] Zhang, W. et al. Optical logic convolutional neural network. *Sci. Adv.* **12**, eaea9278 (2026). 41758942 10.1126/sciadv.aea9278 PMC12947856

[36] Gao, X., Gu, W., Dong, J., Dong, W. & Zhang, X. Performance improvement of all-optical programmable logic array chip with loss-optimized silicon waveguides. *Opt. Express* **33**, 48607–48621 (2025). 41414271 10.1364/OE.579368

[37] Meng, X. et al. Digital-analog hybrid matrix multiplication processor for optical neural networks. *Nat. Commun.* **16**, 7465 (2025). 40796556 10.1038/s41467-025-62586-0 PMC12343823

[38] Zhang, L. et al. Electro-optic directed logic circuit based on microring resonators for XOR/XNOR operations. *Opt. Express* **20**, 11605–11614 (2012). 22714146 10.1364/OE.20.011605

[39] Huo, F. & Gong, G. XOR encryption versus phase encryption, an in-depth analysis. *IEEE Trans. Electromagn. Compat.* **57**, 903–911 (2015).

[40] Irsheidat, S. & Duwairi, R. Brain tumor detection using artificial convolutional neural networks. In *Proc. 2020 11th International Conference on Information and Communication Systems (ICICS)*. 197–203 (IEEE, 2020).

