# Supplementary material for: Reconfigurable optical arithmetic logic unit and its applications
Source: Nat Commun. 2026 Jul 18;17:8793. doi: 10.1038/s41467-026-75750-x (PMC13494030; doi:10.1038/s41467-026-75750-x)
Supplement: Supplementary file 1 [file 41467_2026_75750_MOESM3_ESM.pdf]

nature portfolio

Peer Review File

Reconfigurable optical arithmetic logic unit and its applications

Corresponding Author: Professor Yonghui Tian

This file contains all reviewer reports in order by version, followed by all author rebuttals in order by version.

Version 0:

Reviewer comments:

Reviewer #1

(Remarks to the Author)
This work presents a silicon-based reconfigurable optical arithmetic logic unit (ALU) using microring modulators (MRMs), with demonstrations of basic logic operations, 2-bit arithmetic units and practical applications in encryption and image processing. The study addresses the key challenges of low integration density and limited reconfigurability in photonic computing, and the experimental results on computational density and energy efficiency are promising. Overall, the work is well-designed and the findings are relevant to the field of integrated photonic computing. I think it can be published in Nature Communications. However, several critical points need to be clarified, supplemented and improved to enhance the rigor, completeness and impact of the manuscript. Below are detailed comments and suggestions for revision:
1.The manuscript describes the MRM switching between pass-block and block-pass states for logic operation implementation, but the quantitative electrical tuning parameters (e.g., DC bias voltage range for PN junction carrier depletion, heating power/voltage for TiN microheater wavelength tuning, response time of state switching) are lacking. It is recommended to add a dedicated section or supplementary information to provide these key parameters, and plot the tuning curve of MRM transmission spectrum versus bias voltage/heating power for intuitive illustration.
2.In the high-speed dynamic test (20 Gbit/s), the custom DSP algorithm is used for recovery. However, the specific algorithm framework, key processing steps (e.g., adaptive threshold setting criteria, timing alignment method) and performance evaluation indicators (e.g., bit error rate (BER), eye diagram parameters) of the DSP algorithm are not provided. It is suggested to supplement the algorithm flow chart and BER test results of logic operations/ALU functions at 20 Gbit/s to verify the reliability of the device in practical communication/computing scenarios.
3.The manuscript reports a computational density of 528 Gb/s/mm² and energy efficiency of 12.36 fJ/bit, but the calculation method and definition basis of these two key metrics are not clearly stated (e.g., the effective computing area of the device, whether energy consumption includes thermal tuning power, electrical driving power and DSP processing power). It is recommended to give the specific calculation formula and parameter definition, and distinguish the energy consumption contribution of different components (MRM modulation, thermal tuning, optical amplification, etc.) for quantitative analysis.
4.The current work implements a 2-bit reconfigurable ALU; it is suggested to discuss the scalability of the architecture to higher-bit ALUs (e.g., 4/8-bit), analyze the challenges of cascading basic logic units (e.g., signal attenuation, crosstalk, synchronization) and propose corresponding solutions to demonstrate the potential of the architecture for large-scale photonic computing.
5.The manuscript mentions that MRMs have advantages over MZI modulators in integration density and power efficiency, but only a qualitative comparison is given, and a quantitative comparative analysis based on the same evaluation criteria (e.g., computing density per unit area, energy consumption per bit for the same logic operation) is lacking. It is suggested to conduct a simulation comparison to provide specific data on the performance gap between MRM-based and MZI-based optical logic units.

Reviewer #2

(Remarks to the Author)
Optical computing has become an exceptionally high-interest topic recently, particularly due to the escalating hardware demands of AI and high-performance computing. The manuscript "Reconfigurable optical arithmetic logic unit and its

applications" proposes a reconfigurable computing system based on a combination of basic logical units (BLUs). The authors should be commended for achieving impressive high-speed performance metrics and demonstrating a high computational density of 528Gb/s/mm2. Furthermore, the reported energy efficiency of 12.36fJ/bit shows significant potential for low-power operation. However, while these metrics are noted, the architecture appears to be an incremental evolution of existing directed logic schemes, especially given that experiments were conducted for only two rings instead of the full circuit. To reach the threshold for publication, the authors must address several critical concerns regarding the physical implementation and the theoretical scalability of their design.

Novelty: The proposed "Directed Logic" scheme—using electrical operands to shift MRM resonances for optical routing—has been well-established since 2007. The proposed reconfigurable architecture is a straightforward combination of BLUs. The experiments only demonstrate a two-MRM case due to equipment limitations. Furthermore, the "reconfigurability" cited is a standard property of resonant switches and does not introduce a new computational paradigm compared to recent progress. The authors need to justify the novelty of this specific integration.

Application Depth: The demonstrated applications (2-bit grayscale image processing and ASCII encryption) are visually illustrative but technically rudimentary. Electronic systems currently handle 16-bit to 32-bit precision with far higher reliability; a photonic ALU must demonstrate a clear path toward surpassing these electronic benchmarks in speed or efficiency, which this work does not yet show.

Scalability: The paper demonstrates 1-bit and 2-bit operation. However, there is no clear path to scaling to a multi-bit system without an exponential increase in circuit size. The authors should calculate the number of MRRs required and the potential performance for an N-bit ALU (where = 3, 4, 8...).

Signal Synchronization: Each electrical driving signal is applied to multiple MRRs simultaneously. Synchronizing these signals in a high-speed integrated environment is extremely difficult and significantly increases power consumption. The authors must address this practical implementation challenge.

Full-Circuit Verification: I understand high-speed hardware is limited, but can the authors provide DC performance data for the entire 12-MRR circuit? Demonstrating only two-ring cases is insufficient to prove the full system-level integration.

Reconfigurability Claims: The authors claim functions can be switched without physical redesign. However, the circuits in Figure 1d and Figure 4a appear different. Please clarify if these are the same physical device; otherwise, the claim of "reconfigurability" is misleading.

Minor concerns: Figure 2a: Add a scale bar and clearly mark all optical paths to clarify the logic flow.

Reviewer #3

(Remarks to the Author)
This work reports a reconfigurable optical arithmetic logic unit (ALU) on a silicon on insulator platform, in which microring modulators serve as the core functional elements. The device supports fundamental optical logic operations as well as fully reconfigurable 2-bit arithmetic functions, including addition, subtraction, and digital comparison. In addition to basic functional verification, the manuscript further examines its potential utility in application-oriented scenarios such as high-speed data encryption based on optical XOR operations and image processing tasks including medical image watermarking and pathological tissue difference enhancement, which to some extent extends the discussion beyond proof-of-concept demonstrations. By addressing the current limitations of insufficient functional reconfigurability and low integration density in integrated photonic computing, this study presents a technical advance. The experimental results show that, at an operating speed of 20 Gb/s, the device achieves a computational density of 528 Gb/s/mm^2 and an energy efficiency of 12.36 fJ/bit, with performance metrics that compare favorably with existing electro optical computing schemes and that support the advantages of the MRM based architecture in terms of footprint and power consumption for photonic digital computing. Overall, this research is well-conceived with a rigorous experimental design and solid, reproducible results. The proposed reconfigurable optical ALU enriches the technical repertoire of on-chip photonic computing and provides a versatile hardware scheme for next-generation high-performance computing systems that demand high speed, low power consumption and functional flexibility. This manuscript is deemed suitable for publication in Nature Communications following appropriate revisions to address the critical issues outlined below, which will further enhance the scientific rigor, content completeness and academic impact of the work.
1. The work leverages wavelength-division multiplexing for parallel 2-bit processing, but no quantitative characterization of inter-wavelength crosstalk between the employed wavelengths (e.g., λ_1, λ_2) is provided. What is the measured crosstalk level between the wavelength channels, and how does this crosstalk impact the logic state integrity of parallel arithmetic operations? Please add experimental data on crosstalk and discuss its implications for higher-bit WDM-based processing.
2. The manuscript does not address how the input optical signal power impacts the logic operation performance (e.g., extinction ratio, signal-to-noise ratio (SNR) of the output). What is the minimum/maximum input optical power range for reliable logic/ALU operation?? This is critical for practical system integration where input power may vary.
3.The manuscript touts photonic computing's low latency advantage, but lacks a quantitative breakdown of end-to-end latency (including optical signal propagation, MRM modulation, electrical OR operation). Report total latency for a single 2-bit arithmetic operation, distinguish optical/electrical processing latency ratios, and compare with state-of-the-art electronic 2-bit ALUs.
4. Temperature variations in environments may induce resonance drift in thermally tunable devices within the device, particularly for components such MRRs, thereby affecting operational stability. The authors are encouraged to further

discuss possible temperature stabilization strategies to mitigate such drift, or alternatively to explore device design approaches that can intrinsically reduce thermal sensitivity.
5. A few typos to be corrected:
Line 2, Page 13: “performance”.
Line 11, Page 15: “make as”.
6. Figure 2(a) seems to be lacking a scale bar.
7. In recent two years, there are some significant advances in optical arithmetic logic unit and digital optical computing, the authors should credit these related papers.

Version 1:

Reviewer comments:

Reviewer #2

(Remarks to the Author)
I would like to thank the authors for their detailed and comprehensive responses to my previous comments. The authors have fully addressed all of my concerns. While the proposed time-domain approach to address the scalability concern may not be the ideal solution, it remains an acceptable approach for this stage of development. The authors also have clearly illustrated the inherent trade-off between throughput and operational performance in the revised manuscript. Therefore, I recommend this paper for publication after a thorough proofreading to eliminate minor grammatical issues. For instance, the phrasing in the caption of Table 1 ('1-bit ALUs’ the truth table and expression') needs to be rectified.

Reviewer #3

(Remarks to the Author)
Since the authors have addressed the concerns that the reviewers pointed, I can accept manuscript as it is.

Open Access This Peer Review File is licensed under a Creative Commons Attribution 4.0 International License, which permits use, sharing, adaptation, distribution and reproduction in any medium or format, as long as you give appropriate credit to the original author(s) and the source, provide a link to the Creative Commons license, and indicate if changes were made.
In cases where reviewers are anonymous, credit should be given to 'Anonymous Referee' and the source.
The images or other third party material in this Peer Review File are included in the article’s Creative Commons license, unless indicated otherwise in a credit line to the material. If material is not included in the article’s Creative Commons license and your intended use is not permitted by statutory regulation or exceeds the permitted use, you will need to obtain permission directly from the copyright holder.
To view a copy of this license, visit https://creativecommons.org/licenses/by/4.0/

Dear *Nature Communications* Editors,

Please find enclosed our revised manuscript entitled "Reconfigurable optical arithmetic logic unit and its applications" (Manuscript ID: NCOMMS-26-009247), submitted to *Nature Communications*.

We sincerely thank the Editors and all reviewers for their careful evaluation and constructive comments. These insightful suggestions have significantly improved the clarity, rigor, and overall quality of the manuscript. In the revised version, we have carefully addressed all comments raised by the reviewers and have made corresponding revisions throughout the manuscript.

Kind Regards,

Yonghui Tian

(On behalf of all the authors)

Response to the reviewers' comments

To Reviewer #1's comments:

Recommendation: This work presents a silicon-based reconfigurable optical arithmetic logic unit (ALU) using microring modulators (MRMs), with demonstrations of basic logic operations, 2-bit arithmetic units and practical applications in encryption and image processing. The study addresses the key challenges of low integration density and limited reconfigurability in photonic computing, and the experimental results on computational density and energy efficiency are promising. Overall, the work is well-designed and the findings are relevant to the field of integrated photonic computing. I think it can be published in Nature Communications. However, several critical points need to be clarified, supplemented and improved to enhance the rigor, completeness and impact of the manuscript. Below are detailed comments and suggestions for revision.

Author Response:

We thank the reviewer for the positive and insightful overall assessment of our work. We appreciate the recognition of the significance of our silicon-based reconfigurable optical ALU, particularly in addressing integration density and reconfigurability challenges in photonic computing, as well as the acknowledgment of the experimental results on computational density and energy efficiency.

We also agree with the reviewer that further clarification and refinement are necessary to strengthen the rigor, completeness, and overall impact of the manuscript. In the revised version, we have carefully addressed these points through additional analysis, clearer presentation, and expanded discussion to better support our claims and improve the overall quality of the work.

Comment 1-1:

The manuscript describes the MRM switching between pass-block and block-pass states for logic operation implementation, but the quantitative electrical tuning parameters (e.g., DC bias voltage range for PN junction carrier depletion, heating power/voltage for TiN microheater wavelength tuning, response time of state switching)

are lacking. It is recommended to add a dedicated section or supplementary information to provide these key parameters, and plot the tuning curve of MRM transmission spectrum versus bias voltage/heating power for intuitive illustration.

Response:

We thank the reviewer for this important and constructive suggestion. We agree that a quantitative description of the electrical tuning parameters is essential for improving the rigor and completeness of the manuscript.

In the revised supplementary information, we have added a dedicated subsection (Supplementary Note S2) to provide a comprehensive characterization of the microring modulator (MRM) tuning behavior, specifically:

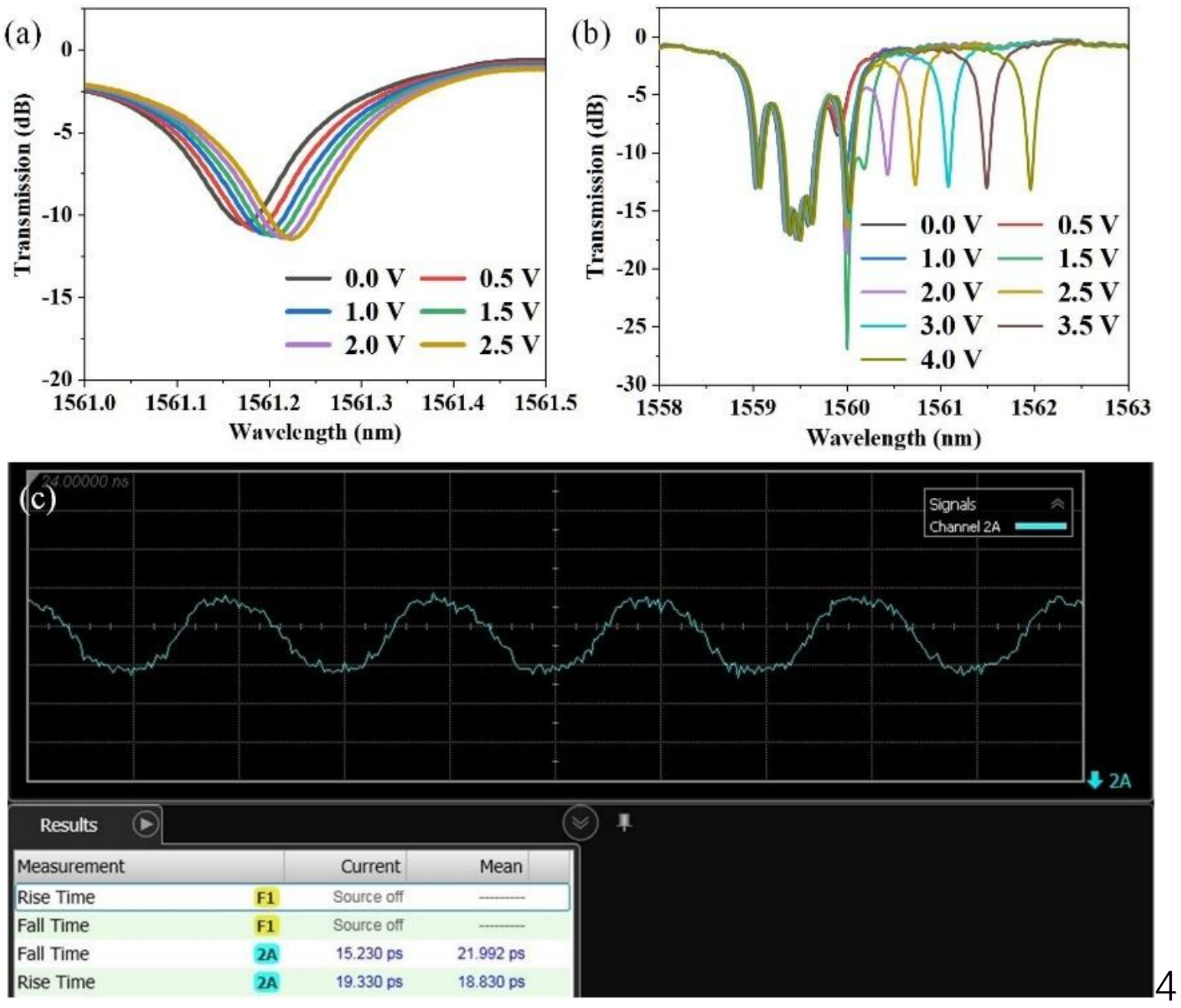

Figure R1. Characterization of a silicon MRM. (a) Resonance shift under different PN junction reverse voltage. (b) Resonance shift under various microheater voltages. (c) Rising and falling edges at 20 Gbit/s modulation.

(1) Electrical tuning via PN junction carrier depletion.

We have included the DC bias voltage range used for switching between the pass–block and block–pass states, along with the corresponding transmission spectra. The relationship between resonance shift (or transmission contrast) and applied bias voltage is now presented to clearly illustrate the modulation mechanism and operating window.

(2) Thermal tuning via TiN microheater.

We have provided the heating voltage/power required for wavelength alignment, together with the measured resonance shift as a function of heating power. This allows direct evaluation of the tuning efficiency and power consumption.

(3) Dynamic response of state switching.

The response time of the MRM under electrical modulation has been experimentally characterized and added to the manuscript, demonstrating the switching speed relevant to logic operations.

In conclusion, following the reviewer's suggestion, we have included plots of the transmission spectrum as a function of bias voltage and heating power, which provide a more intuitive visualization of the transition between logic states.

We believe that these additions significantly improve the transparency of the device operation and strengthen the experimental foundation of the work. Furthermore, we add this section in Supplementary Note 2 as follow:

Supplementary Note 3: Electrical and Thermal Tuning Characteristics of the MRM

To quantitatively characterize the switching behavior of the MRM used for logic operation, we systematically investigate its electrical and thermal tuning responses, including carrier-depletion-induced modulation, thermo-optic wavelength alignment, and dynamic switching performance.

(1) Electrical tuning via PN junction carrier depletion.

The MRM operates under reverse-bias conditions to modulate the effective refractive index through carrier depletion. The applied DC bias voltage is swept from V_{min} to V_{max}, covering the full transition between the pass–block and block–pass states. The transmission spectra are recorded for each bias point, from which the resonance shift and extinction ratio are extracted.

The measured tuning curve (Supplementary Fig. S2a) shows a monotonic dependence of resonance shift (and corresponding transmission) on the applied bias voltage, confirming stable and continuous electrical control within the operating window.

(2) Thermal tuning via TiN microheater.

To compensate for fabrication-induced wavelength detuning and environmental fluctuations, a TiN microheater is integrated for thermo-optic tuning. The heating voltage (or equivalently electrical power) is varied from 0V to 4V, and the resulting resonance shift is extracted from the measured spectra.

The resonance wavelength exhibits a non-linear dependence on heating power within the operating range (Supplementary Fig. S2b), allowing precise alignment to the target wavelength.

(3) Dynamic response of state switching.

The temporal response of the MRM is characterized by applying a square-wave electrical signal and recording the output optical intensity using a high-speed photodetector. The rise time (t_r) and fall time (t_f) are extracted based on the 10–90% transition criterion.

The measured response (Supplementary Figure S2c) confirms that the device supports picosecond-scale switching speeds, which are sufficient for the targeted logic operation bandwidth.

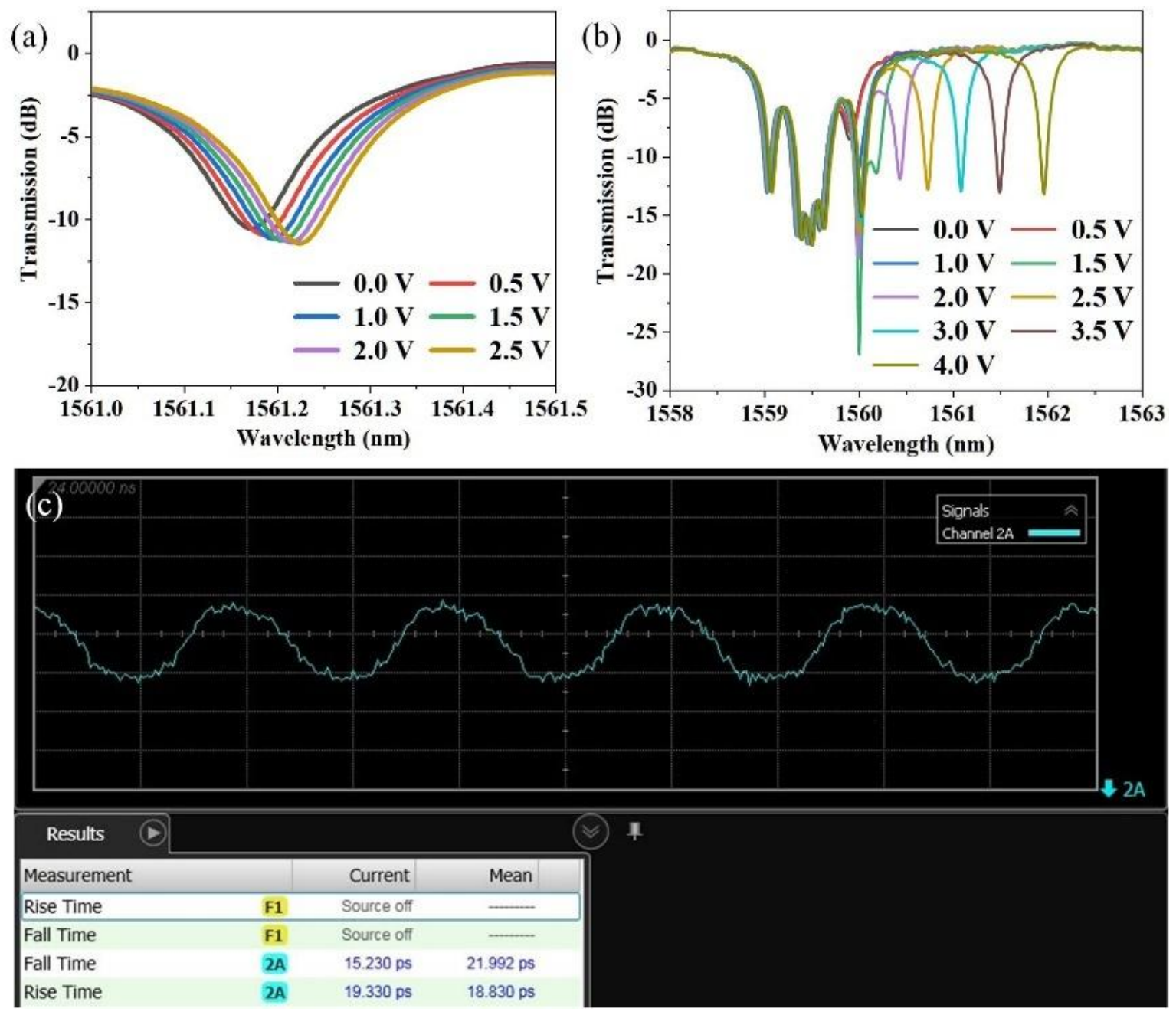

Figure S2 Characterization of the MRM. (a) Transmission spectra of the MRM under varying bias voltages. (b) Transmission spectra of the MRM under thermal tuning. (c) Rising and falling edges of the MRM at 20 Gbit/s

Comment 1-2:

In the high-speed dynamic test (20 Gbit/s), the custom DSP algorithm is used for recovery. However, the specific algorithm framework, key processing steps (e.g., adaptive threshold setting criteria, timing alignment method) and performance evaluation indicators (e.g., bit error rate (BER), eye diagram parameters) of the DSP algorithm are not provided. It is suggested to supplement the algorithm flow chart and BER test results of logic operations/ALU functions at 20 Gbit/s to verify the reliability of the device in practical communication/computing scenarios.

Response:

We thank the reviewer for this insightful and technically important suggestion. We fully agree that a clearer description of the DSP algorithm and quantitative evaluation of high-speed performance are essential to support the reliability of the demonstrated 20 Gbit/s operation.

In the revised Supplementary Note 6, we have added a dedicated description of the DSP processing framework, together with the corresponding algorithm flow chart. The main processing steps are explicitly clarified, including:

(1) Data frame identification and timing alignment.

The beginning of the data sequence is first determined, followed by timing alignment to ensure correct sampling of each bit.

(2) Adaptive threshold setting.

As illustrated in the flow chart, the decision threshold is dynamically determined based on the received signal power and duty cycle, allowing robust discrimination between logic states under high-speed operation.

(3) Logic-level decision and data reconstruction.

The sampled signals are then mapped to logic states according to the optimized threshold, enabling recovery of the ALU/logic outputs.

Regarding performance evaluation, we have performed additional experiments to provide a direct and quantitative assessment of high-speed operation. Specifically, a 10,000-bit non-return-to-zero (NRZ) data sequence at 20 Gbit/s is generated and measured. The acquired waveform is processed using the proposed DSP framework,

and the reconstructed data are directly compared with the transmitted reference sequence. Based on this analysis, an error rate of 4×10^{-4} is obtained (i.e., 4 errors in 10,000 bits), which has been added to the revised Supplementary Information. This bit error rate is much lower than the typical hard-decision forward error correction (HD-FEC) threshold of 3.8×10^{-3}, indicating that lossless transmission can be achieved through backend error correction.

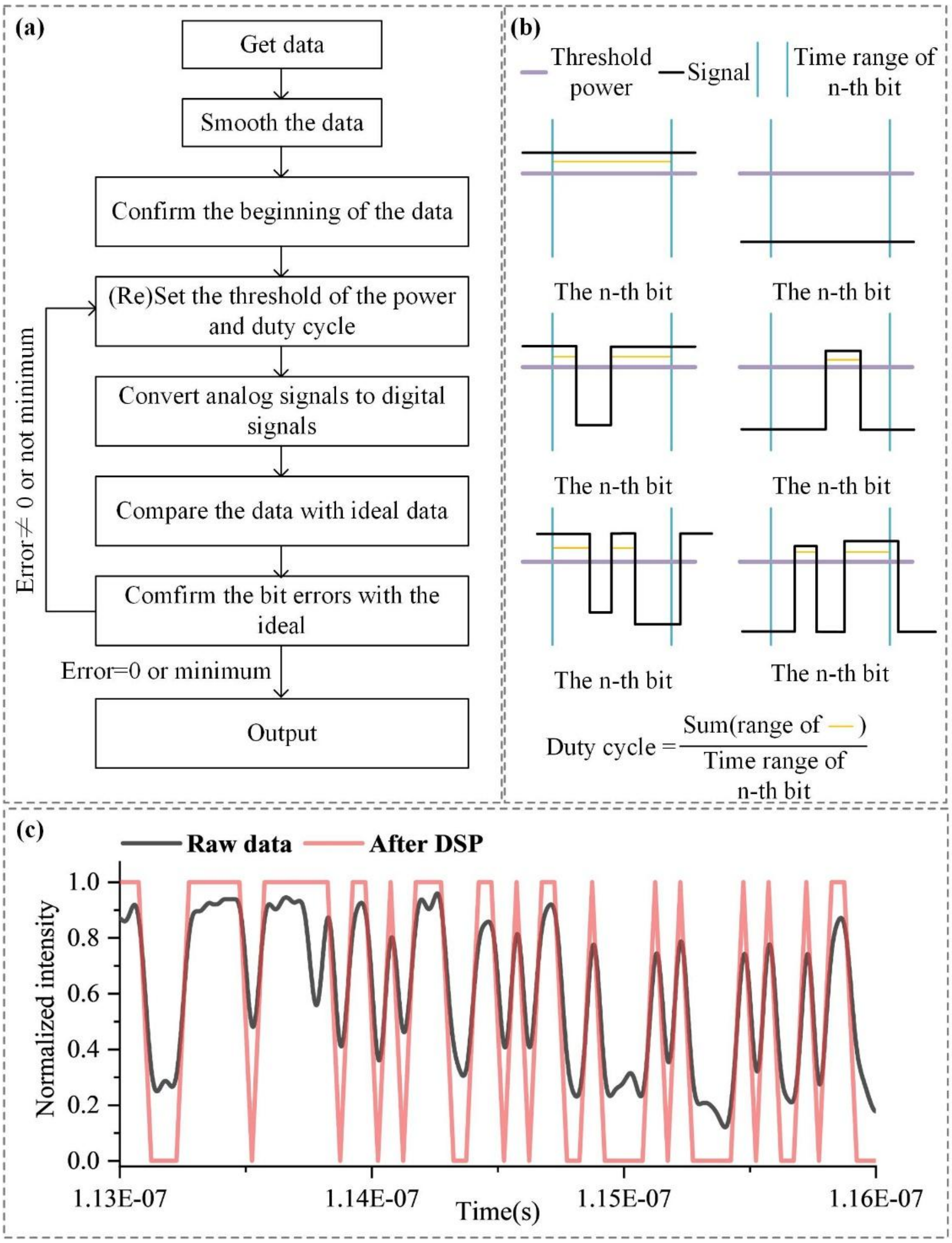

Figure R2. DSP framework for data recovery and its validation at 20 Gbit/s. (a) Three-stage DSP workflow: frame identification, adaptive threshold, and logic decision. (b) Joint decision criteria: amplitude statistics and duty cycle. (c) NRZ waveforms before and after DSP processing.

To further understand the origin of the observed errors, we analyze the corresponding waveform segments and find that most errors occur in specific bit patterns involving long consecutive logic "1" sequences followed by an isolated logic "0". In these cases, the microring modulator cannot fully complete the state transition within a single bit period, leading to incomplete extinction and subsequent misclassification. This behavior is attributed to the finite dynamic response of the device (e.g., carrier dynamics and cavity photon lifetime), rather than limitations of the DSP algorithm itself.

In addition, we provide a comparison of the waveform before and after DSP processing, as shown in Figure R2c, which illustrates the effectiveness of the signal reconstruction and the consistency between the processed signal and the identified error events.

We believe that these revisions provide a more rigorous and quantitative validation of the high-speed performance and significantly improve the technical completeness of the manuscript. Furthermore, we add this section in Supplementary Note 6 as follow:

Supplementary Note 6: DSP description.

The optical waveforms acquired from the digital communications analyzer (DCA) are continuous-time analog signals, which cannot be directly interpreted as discrete binary logic levels due to amplitude fluctuations, timing jitter, and waveform distortion, especially under high-speed operation at 20 Gbit/s. To enable reliable data recovery and quantitative performance evaluation, a dedicated digital signal processing (DSP) framework is developed to convert the measured analog traces into standardized digital sequences.

As illustrated in Figure S8a, the DSP workflow consists of three main stages: data frame identification and timing alignment, adaptive threshold determination, and logic-level decision for data reconstruction. The corresponding decision criteria are summarized in Figure S8b, while the effectiveness of the DSP processing is validated through NRZ comparison before and after processing in Figure S8c.

(1) Data frame identification and timing alignment

Due to the lack of strict synchronization between the arbitrary waveform generator (AWG) and the DCA, the captured waveform typically contains an arbitrary temporal offset relative to the transmitted data sequence. This misalignment, if uncorrected, leads to incorrect bit segmentation and degraded logic interpretation.

To address this issue, a cross-correlation-based synchronization method is employed. The measured waveform is correlated with a predefined reference sequence, and the temporal position corresponding to the maximum correlation coefficient is identified as the starting point of the valid data frame. This procedure effectively aligns the measured signal with the ideal bit sequence.

Once the starting position is determined, the waveform is segmented into discrete bit intervals according to the known data rate, with a bit duration of. This ensures that each bit is sampled within the correct temporal window, minimizing inter-symbol interference induced by timing offsets. The robustness of this synchronization step is critical for subsequent threshold-based decision processes.

(2) Adaptive threshold determination based on amplitude and duty-cycle statistics

At high data rates, the received optical signal exhibits non-ideal characteristics, including amplitude variation, baseline drift, and waveform asymmetry. Under such conditions, a fixed decision threshold is insufficient for reliable logic discrimination. Therefore, an adaptive thresholding strategy is adopted.

As shown in Figure S8b, the decision framework incorporates both amplitude-based and duty-cycle-based criteria:

Amplitude threshold:

The statistical distribution of the signal intensity within a data frame is analyzed to extract representative high-level and low-level values. Based on this distribution, an optimal decision threshold is defined to maximize the separation between logic “1” and logic “0” levels.

Duty-cycle criterion:

For each bit interval, the proportion of time during which the signal remains above the threshold is calculated. This duty-cycle metric provides an additional constraint to

mitigate errors caused by waveform distortion, incomplete transitions, or timing jitter.

By jointly considering these two criteria, the algorithm achieves robust logic discrimination even when the waveform deviates from an ideal rectangular shape. This hybrid strategy is particularly important for maintaining decision reliability under 20 Gbit/s operation, where signal degradation becomes more pronounced.

(3) Logic-level decision and data reconstruction

Following synchronization and threshold determination, binary logic values are assigned to each bit interval. Instead of relying solely on instantaneous sampling or edge detection, the decision process is based on a statistical evaluation of the signal within each bit period.

Specifically, a bit is classified as logic “1” if (i) the average signal level exceeds the adaptive threshold and (ii) the duty-cycle criterion satisfies a predefined condition (i.e., the signal remains above threshold for a dominant fraction of the bit duration). Otherwise, the bit is assigned as logic “0”.

This dual-criterion decision mechanism improves robustness against noise, timing jitter, and partial waveform transitions, ensuring stable data reconstruction across varying signal conditions. The reconstructed binary sequence is subsequently used to recover the logic outputs of the implemented ALU operations.

(4) Validation through long-sequence NRZ data and error analysis

To quantitatively evaluate the reliability of the proposed DSP framework under high-speed operation, we perform data transmission experiments using a non-return-to-zero (NRZ) signal with a sequence length of 10,000 bits at 20 Gbit/s. The experimentally acquired waveform is processed using the DSP procedure described above, and the reconstructed binary sequence is directly compared with the transmitted reference data.

The measured error rate is 4×10^{-4}, corresponding to 4 errors per 10,000 bits. This result provides a direct assessment of system-level performance beyond qualitative visualization metrics such as eye diagrams.

To further understand the origin of the observed errors, we analyze the temporal waveform associated with erroneous bit positions. It is found that the dominant error

mechanism arises in specific bit patterns characterized by long sequences of consecutive logic “1” followed by an isolated logic “0”. In such cases, the MRM (MRM) exhibits a limited transient response when switching from a sustained high-transmission state to a low-transmission state, leading to incomplete extinction within a single period.

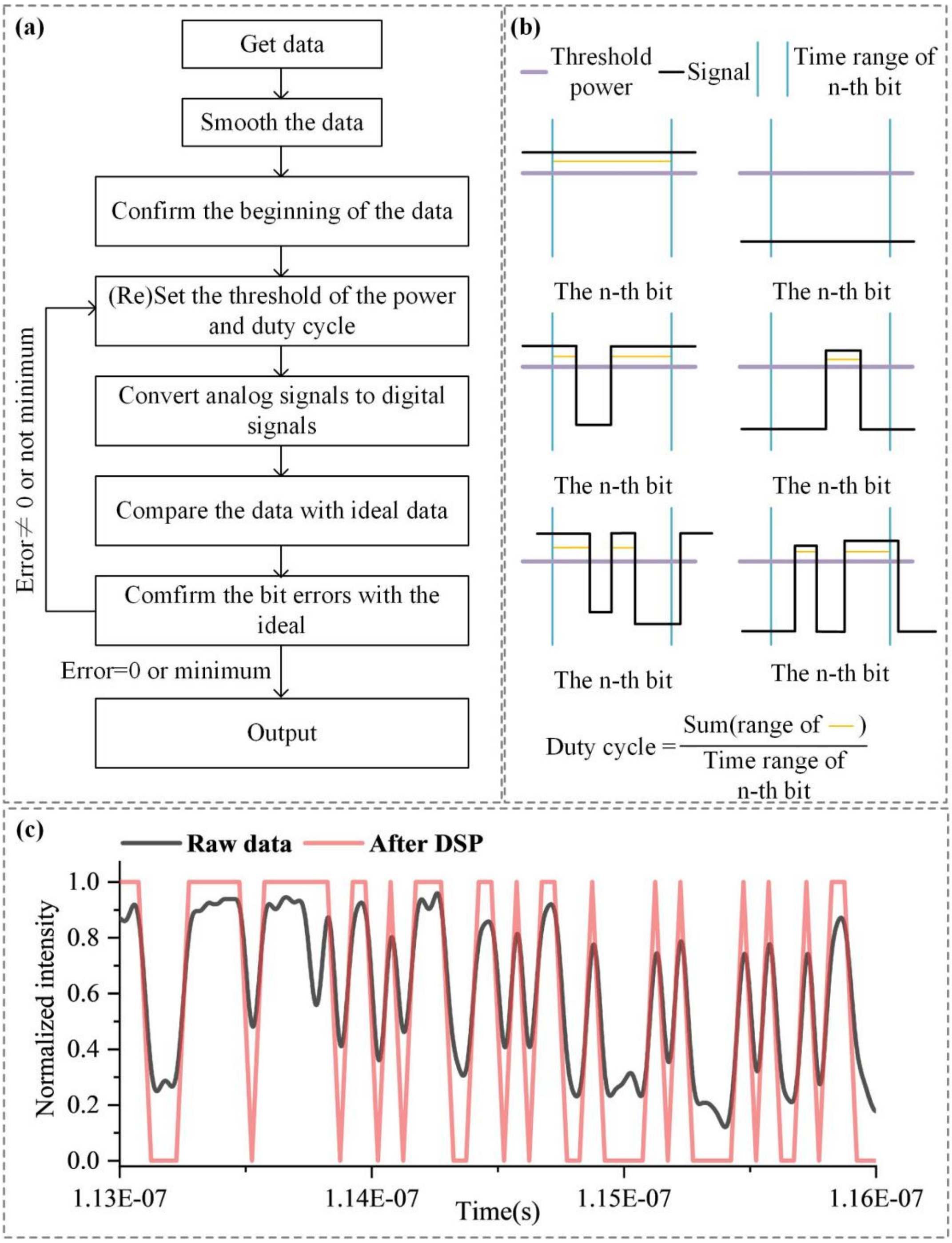

Figure S8. DSP framework for data recovery and its validation at 20 Gbit/s. a Three-stage DSP workflow: frame identification, adaptive threshold, and logic decision. **b** Joint decision criteria: amplitude statistics and duty cycle. **c** NRZ waveforms before and after DSP processing.

This behavior can be attributed to the combined effects of carrier dynamics and cavity photon lifetime, which introduce a finite response time and prevent ideal instantaneous switching. As a result, the optical intensity within the interval does not fully cross the decision threshold, leading to misclassification by the DSP algorithm.

Importantly, this error mechanism is rooted in the physical response limitation of the device rather than the DSP processing itself. The DSP framework faithfully reconstructs the waveform based on the defined decision criteria and does not artificially suppress or introduce errors.

For clarity, Figure S8c presents a comparison of the waveform before and after DSP processing. While the raw waveform exhibits amplitude fluctuation and timing uncertainty, the processed signal demonstrates clear logic-level discretization. The residual errors observed in the reconstructed sequence are consistent with the identified device-level limitations.

These results indicate that the proposed DSP framework enables reliable data recovery at 20 Gbit/s, while the remaining errors highlight the intrinsic speed limitation of the current device configuration under specific input patterns.

Comment 1-3:

The manuscript reports a computational density of 528 Gb/s/mm² and energy efficiency of 12.36 fJ/bit, but the calculation method and definition basis of these two key metrics are not clearly stated (e.g., the effective computing area of the device, whether energy consumption includes thermal tuning power, electrical driving power and DSP processing power). It is recommended to give the specific calculation formula and parameter definition, and distinguish the energy consumption contribution of different components (MRM modulation, thermal tuning, optical amplification, etc.) for quantitative analysis.

Response:

We thank the reviewer for this important suggestion regarding the clarification of computational density and energy efficiency. We agree that explicit definitions and calculation methods are essential for a rigorous and transparent evaluation.

In the revised manuscript and Supplementary Note 7, we have clarified the

calculation formulas, parameter definitions, and the contribution of different components, as summarized below.

(1) Definition and calculation of computational density

The computational density is defined as the total data processing throughput normalized by the effective footprint of the photonic computing core. Specifically, it is calculated as:

$$Computational\ density = \frac{N_{MRM} \times R_R}{A_{CORE}}$$

where N_{MRM} is the number of microring modulators involved in parallel or cascaded computation, R_R is the operating data rate per MRM, and A_{core} is the physical footprint of the photonic computing region.

In this work, a total of 12 MRMs is cascaded to implement the 2-bit ALU, each operating at 20 Gbit/s. The footprint of the device is 1.42 ×0.32 mm^2. Therefore, the computational density is calculated as: (20 Gbit/s× 12)/ (1.42 ×0.32 mm^2) ≈ 528 Gbit/s/mm^2.

(2) Definition and breakdown of energy efficiency

We further clarify that the reported energy efficiency includes contributions from both dynamic modulation and thermal tuning of the MRMs.

Per-device energy consumption: Each MRM exhibits an energy consumption of 1.03 fJ/bit, consisting of: 0.79 fJ/bit for electrical modulation (carrier depletion), 0.24 fJ/bit for thermal tuning (wavelength stabilization). [V1]

System-level energy efficiency: For the implemented 2-bit ALU, the total energy efficiency of 12.36 fJ/bit is obtained by accounting for all MRMs involved in the computation. This value corresponds to the cumulative on-chip energy consumption of the photonic computing core during operation.

(3) Scope and exclusion of off-chip components

The above energy analysis considers only the on-chip photonic components, i.e., the MRMs responsible for computation. External components in the experimental setup, such as lasers, erbium-doped fiber amplifiers (EDFAs), arbitrary waveform generators (AWGs), and digital communications analyzers (DCAs), are not included in the

reported energy efficiency.

(4) Discussion on system-level integration

We further note that recent advances in photonic integration have demonstrated the feasibility of integrating light sources, modulators, and detection units onto a single chip, which can significantly reduce overall system power consumption. While such integration is beyond the scope of the present work, it provides a promising pathway toward improving the practical energy efficiency of photonic computing systems. Furthermore, we revise this section in Supplementary Note 6 as follow:

"For the computational density, the architecture cascade 12 MRMs to achieve the 2-bit ALU, both of the MRM's modulation speed are 20 Gbit/s. As mentioned in the manuscript, the footprint of the device is 1.42 ×0.32 mm^2, so the computational density can be calculated as (20 Gbit/s× 12)/ (1.42 ×0.32 mm^2) $\approx$ 528 Gbit/s/mm^2.

The power consumption mentioned above takes into account only the on-chip MRM component; in actual experimental setups, the power consumption of devices such as lasers, EDFAs, AWGs, and DCAs must also be considered. Recent reports indicate that integrating all these components onto a single chip can significantly reduce the power consumption of the optical chip."

References

V1 Timurdogan E, Sorace-Agaskar CM, Sun J, Shah Hosseini E, Biberman A, Watts MR. An ultralow power athermal silicon modulator. ***Nat Commun*** 5, 4008 (2014).

Comment 1-4:

The current work implements a 2-bit reconfigurable ALU; it is suggested to discuss the scalability of the architecture to higher-bit ALUs (e.g., 4/8-bit), analyze the challenges of cascading basic logic units (e.g., signal attenuation, crosstalk, synchronization) and propose corresponding solutions to demonstrate the potential of the architecture for large-scale photonic computing.

Response:

We thank the reviewer for this critical and technically rigorous comment. We agree that the scalability of the proposed architecture toward multi-bit operation must be

quantitatively analyzed, particularly in terms of device count and performance evolution.

As correctly pointed out by the reviewer, a straightforward extension of the same spatial-parallel design principle would lead to a rapid increase in the number of required MRMs, thereby increasing implementation complexity. In this work, the demonstrated 2-bit ALU is designed as a parallel prototype, which represents the minimal functional unit for arithmetic operations (e.g., addition and subtraction). To address scalability, we clarify that higher-bit operations can be realized using a time-domain iterative computation strategy, rather than purely spatial scaling.

(1) Multi-bit adder implementation via time-domain multiplexing

Taking the adder as an example, the input signals can be decomposed into three components: the initial carry (C_0), and two operand sequences ($A = A_iA_{i-1}\ldots A_3A_2A_1$) and ($B = B_iB_{i-1}\ldots B_3B_2B_1$). Through time-division multiplexing (TDM), the data streams of A and B are encoded into temporal sequences and sequentially loaded onto the corresponding MRMs.

At each time slot, the signals A_i, B_i and C_{i-1} are applied to the MRM-based logic unit, generating the sum *Si* and carry C_i. The carry signal C_i. is optically split into multiple paths, where a portion is fed back as the input carry C_{i-1} for the next computation stage via a conventional optical–electrical–optical (OEO) loop, while the remaining portion is used for output acquisition. In this way, a sequence of carry bits $C_1C_2\ldots C_i$ is generated iteratively.

For addition, only the most significant carry bit is required as the final output, while all sum bits Si are retained. The iterative carry computation can be efficiently managed through backend DSP, enabling multi-bit addition without requiring a proportional increase in hardware resources.

This approach allows the realization of higher-bit adders with constant hardware complexity, where the scalability is primarily limited by the efficiency of OEO conversion and the minimum signal power required for reliable operation.

Table R1 2-bit ALUs' expression

Adder	C_i	$C_{i-1}(A_i \oplus B_i) + (A_i \bullet B_i)$
	S_i	$A_i \oplus B_i \oplus C_{i-1}$
Subtractor	C_i	$C_{i-1}(\overline{A_i \oplus B_i}) + (\overline{A_i} \bullet B_i)$
	D_i	$A_i \oplus B_i \oplus C_{i-1}$
Comparator	$F_{A>B}$	$A_2 \bullet \overline{B_2} + (A_2 \odot B_2)(A_1 \bullet \overline{B_1})$
	$F_{A<B}$	$\overline{A_2} \bullet B_2 + (A_2 \odot B_2)(\overline{A_1} \bullet B_1)$
	$F_{A=B}$	$(A_1 \odot B_1) + (A_2 \odot B_2)$

(2) Subtractor scalability

The subtractor follows a similar structure to the adder, as the expressions in Table R1 share the same logical form. By appropriately reconfiguring the MRM states and input ports, the same time-domain iterative scheme can be applied to realize multi-bit subtraction without additional hardware overhead.

(3) Comparator scalability

In contrast to adders and subtractors, comparators exhibit a different scaling behavior. The number of required BLU operations increases with bit width due to the need for multi-bit comparison logic. Specifically, a 2-bit comparator requires 3 BLU operations, a 3-bit comparator requires 6, and a 4-bit comparator requires 10. Extending this trend, an 8-bit comparator would require 36 BLU operations. Unlike arithmetic units, the comparator logic does not readily reduce to a simple iterative structure. Therefore, the time-domain reuse strategy is less effective. In our architecture, the BLU operations can still be executed in the optical domain, while the final decision logic can be completed in the electrical domain to maintain system efficiency.

(4) Trade-off between scalability and throughput

We emphasize that the proposed time-domain approach enables multi-bit operation without exponential growth in hardware resources, effectively avoiding the scalability limitations of purely spatial implementations. However, this comes with a

trade-off: as the bit width increases, the computation transitions from parallel to partially serial operation, leading to a reduction in overall throughput under a fixed modulation rate.

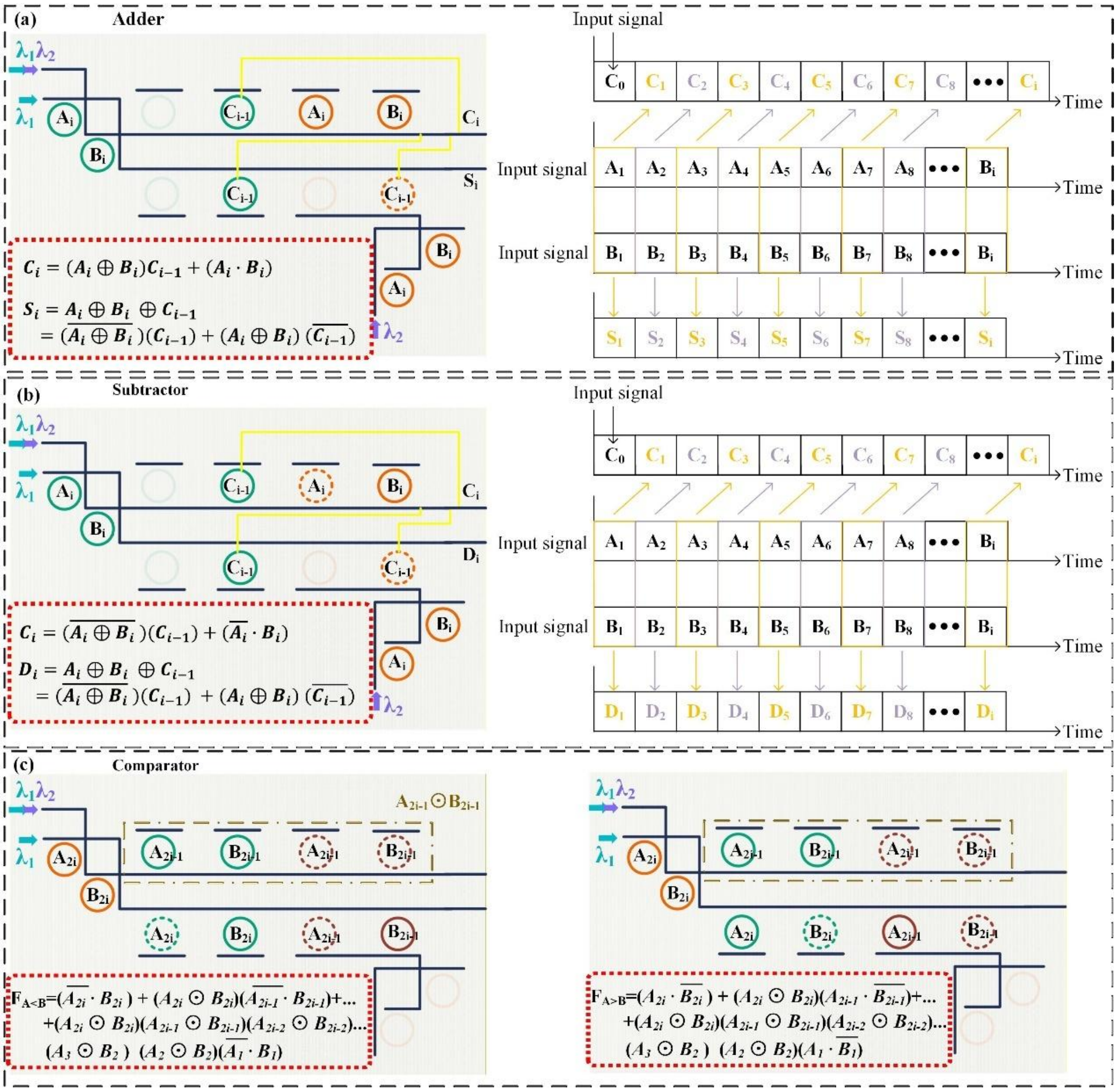

Figure R3. Schemes for bit-width extension of arithmetic units. (a) Bit-width extension scheme for full adders. (b) Bit-width extension scheme for full subtractors. (c) Bit-width extension scheme for comparators.

In summary, while a native spatial extension would lead to a rapid increase in circuit size, the proposed architecture provides a feasible and resource-efficient pathway toward multi-bit ALUs through time-domain multiplexing and iterative computation. This approach transforms the scaling behavior from spatial resource growth to temporal processing overhead. As a result, the number of required MRMs can remain approximately constant for arithmetic operations (adder/subtractor), while

the effective computation rate scales inversely with bit width.

We have revised the manuscript to explicitly include this scalability analysis, along with quantitative examples of BLU usage for different bit widths. We believe this clarification addresses the reviewer’s concern and provides a more complete picture of the scalability and practical design trade-offs of the proposed architecture. Furthermore, we add this section in Supplementary Note 9 as follow:

Supplementary Note 9: Scalability toward multi-bit photonic ALUs

To evaluate the potential of the proposed architecture for large-scale photonic computing, we analyze its scalability toward higher-bit ALUs, and discuss the associated challenges and feasible mitigation strategies.

A direct spatial extension of the demonstrated 2-bit architecture would require a proportional increase in the number of MRMs, leading to rapidly growing circuit complexity and footprint. To address this limitation, the proposed architecture adopts a time-domain iterative computation strategy, which enables multi-bit operation without proportional hardware scaling.

Taking the adder as an example, the input operands A·B are decomposed into bit sequences and encoded into time-division multiplexed (TDM) signals. At each time slot, the corresponding bits A_i, B_i, and the carry input C_{i-1} are applied to the MRM-based logic unit, generating the sum S_i and carry C_i. The carry signal is partially fed back through an optical–electrical–optical (OEO) loop to serve as the input for the next time step, enabling iterative computation across multiple bit positions. Under this scheme, multi-bit addition and subtraction can be realized with approximately constant hardware resources, where scalability is primarily determined by temporal processing depth rather than spatial circuit size.

The scalability characteristics differ for arithmetic and comparison operations. For addition and subtraction, the iterative structure allows efficient reuse of the same set of BLUs. In contrast, comparator functions require an increasing number of BLU operations with bit width due to multi-bit comparison logic. For example, a 2-bit comparator requires 3 BLUs, while 4-bit and 8-bit implementations require 10 and 36

BLUs, respectively. To maintain system efficiency, the architecture performs core comparison operations in the optical domain, while the final decision logic can be completed in the electrical domain.

Scaling to larger systems introduces several practical challenges associated with cascading photonic logic units. Optical signal attenuation accumulates due to waveguide propagation loss, coupling loss, and device insertion loss, which can degrade signal integrity in deep cascaded configurations. This can be mitigated through low-loss waveguide design, optimized coupling structures, and, when necessary, the incorporation of optical amplification stages or regeneration mechanisms. Crosstalk and spectral interference may arise from dense integration of resonant devices, particularly under closely spaced wavelength channels. These effects can be reduced through careful resonance engineering, wavelength allocation, and layout-level isolation strategies.

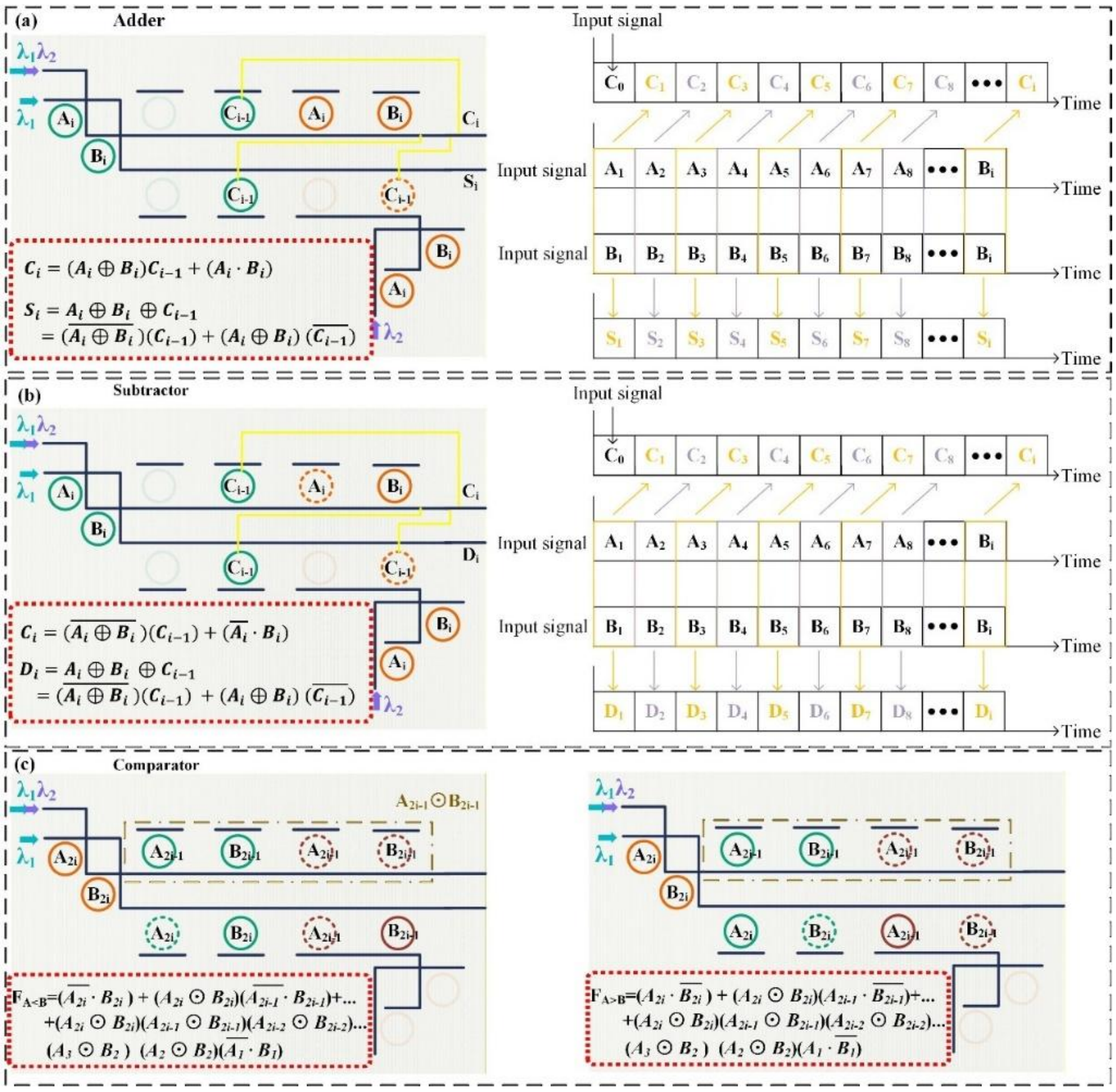

Figure S10. Schemes for bit-width extension of arithmetic units. (a) Bit-width extension scheme for full adders. (b) Bit-width extension scheme for full subtractors. (c) Bit-width extension scheme for comparators.

Signal synchronization is another critical consideration in multi-channel operation. However, this requirement is consistent with standard practices in high-speed electronic–photonic integrated systems and can be addressed through co-design of driver electronics and photonic devices, matched interconnect routing, and localized clock distribution. In addition, the use of time-domain multiplexing reduces the number of simultaneously active channels, thereby relaxing synchronization constraints in large-scale implementations.

The adoption of time-domain iterative processing introduces a fundamental trade-off between scalability and throughput. As the bit width increases, the computation becomes partially serialized, leading to a reduction in effective throughput under a fixed modulation rate. Nevertheless, given the high intrinsic speed of photonic devices, the resulting performance remains competitive for high-bandwidth applications.

Overall, the proposed architecture transforms the scaling behaviour from spatial resource growth to temporal processing overhead, providing a feasible pathway toward multi-bit photonic ALUs. Combined with advances in photonic integration and system-level co-design, this approach offers a scalable foundation for future large-scale photonic computing systems.

Comment 1-5:

The manuscript mentions that MRMs have advantages over MZI modulators in integration density and power efficiency, but only a qualitative comparison is given, and a quantitative comparative analysis based on the same evaluation criteria (e.g., computing density per unit area, energy consumption per bit for the same logic operation) is lacking. It is suggested to conduct a simulation comparison to provide specific data on the performance gap between MRM-based and MZI-based optical logic units.

Response:

We thank the reviewer for this important and constructive suggestion. We fully agree that a quantitative comparison between MRMs and MZI modulators under consistent evaluation criteria is necessary to rigorously support the claimed advantages.

To ensure fairness, both MRM- and MZI-based implementations are evaluated

under the same assumptions, including identical logic functionality (2-bit ALU), operating data rate (20 Gbit/s), and comparable silicon photonic platforms.

(1) Computational density and footprint comparison

The computational density is fundamentally constrained by the device footprint. In our implementation, the MRM employs a compact resonant structure with a radius of 20 μm, leading to a footprint on the order of 10^2 $μm^2$ per device.

In contrast, a silicon-based MZI modulator typically requires millimeter-scale phase shifters to accumulate sufficient phase shift (π phase shift), due to the relatively weak electro-optic effect in silicon. As a result, the footprint of a single MZI modulator is generally on the order of 10^6 $μm^2$., which is 3–4 orders of magnitude larger than that of an MRM.

When scaled to multi-device logic architectures (e.g., the 12-element configuration used in this work), this footprint difference directly translates into a substantial gap in computational density. Under the same functionality and data rate, the MRM-based architecture achieves significantly higher integration density due to its compact resonant geometry.

(2) Driving voltage and switching mechanism

Another key difference lies in the required electrical driving conditions. The MRM operates based on resonance tuning, where relatively small refractive index changes are sufficient to switch between pass–block and block–pass states. As a result, the required voltage swing is relatively low.

In contrast, MZI modulators rely on achieving a π phase shift between the two interferometer arms. This typically requires a larger voltage-length product ($V_{\Pi}L$), leading to higher driving voltages and/or longer phase shifters. Consequently, MZI-based implementations generally exhibit higher electrical driving energy under comparable operating conditions.

This difference becomes particularly relevant for logic operations, where frequent state switching is required. The ability of MRMs to achieve switching with lower voltage directly contributes to reduced energy consumption per bit.

(3) Modulation efficiency and energy consumption

The improved energy efficiency of MRMs can be further understood from the perspective of resonant enhancement. In MRMs, the optical field circulates within the cavity, effectively increasing the interaction length between light and the modulating medium. This resonance effect enables a stronger modulation response for a given refractive index change.

In contrast, MZI modulators rely on single-pass interference, where the modulation efficiency is limited by the physical length of the phase shifter. As a result, achieving the same extinction ratio typically requires either longer devices or higher drive voltages.

Under equivalent operating conditions, this difference leads to a lower energy consumption per bit for MRM-based implementations.

(4) Trade-offs and application scope

For completeness, we also emphasize that MZI-based modulators offer distinct advantages, including broader optical bandwidth, reduced sensitivity to wavelength detuning, and simpler bias control. In contrast, MRMs require precise wavelength alignment and are more sensitive to temperature variations due to their resonant nature.

Therefore, the choice between MRM- and MZI-based architectures depends on the target application: MRMs are particularly advantageous for high-density and energy-efficient computing, while MZIs may be preferred in broadband and wavelength-insensitive systems.

We believe that this expanded and quantitative comparison provides a clearer and more rigorous justification of the advantages of the proposed MRM-based architecture under consistent evaluation criteria.

To Reviewer #2 's comments:

Recommendation: Optical computing has become an exceptionally high-interest topic recently, particularly due to the escalating hardware demands of AI and high-performance computing. The manuscript "Reconfigurable optical arithmetic logic unit and its applications" proposes a reconfigurable computing system based on a combination of basic logical units (BLUs). The authors should be commended for achieving impressive high-speed performance metrics and demonstrating a high

computational density of 528Gb/s/mm2. Furthermore, the reported energy efficiency of 12.36fJ/bit shows significant potential for low-power operation. However, while these metrics are noted, the architecture appears to be an incremental evolution of existing directed logic schemes, especially given that experiments were conducted for only two rings instead of the full circuit. To reach the threshold for publication, the authors must address several critical concerns regarding the physical implementation and the theoretical scalability of their design.

Author Response:

We sincerely thank the reviewer for the thorough and critical assessment of our manuscript. We appreciate the recognition of the relevance of this work in the context of optical computing for AI and high-performance systems, as well as the acknowledgment of the achieved high-speed performance, computational density, and energy efficiency.

We also take seriously the reviewer's concerns regarding the level of novelty, the current extent of experimental implementation, and the scalability of the proposed architecture. We agree that these aspects are crucial for evaluating the broader impact and practical potential of the work. In the revised manuscript, we have carefully addressed these points by strengthening the clarification of the architectural contributions, expanding the discussion on physical implementation constraints, and providing a more comprehensive analysis of scalability and system-level considerations.

Specifically, we have revised the manuscript to more clearly position our approach relative to existing directed logic schemes, highlighting the distinctions in reconfigurability and system integration. We have also clarified the scope of the current experimental demonstrations and their relationship to the full ALU architecture, while adding detailed discussions on the challenges and feasible pathways toward large-scale implementation.

We believe these revisions significantly improve the rigor, clarity, and positioning of the manuscript, and we hope that the revised version adequately addresses the reviewer's concerns.

Comment 2-1:

Novelty: The proposed "Directed Logic" scheme—using electrical operands to shift MRM resonances for optical routing—has been well-established since 2007. The proposed reconfigurable architecture is a straightforward combination of BLUs. The experiments only demonstrate a two-MRM case due to equipment limitations. Furthermore, the "reconfigurability" cited is a standard property of resonant switches and does not introduce a new computational paradigm compared to recent progress. The authors need to justify the novelty of this specific integration.

Author Response:

We thank the reviewer for this rigorous and insightful comment regarding the novelty of our work. Note that microring-based directed logic and resonance-shift-based optical routing have been extensively studied since early demonstrations, and reconfigurability at the device level is an inherent property of resonant photonic structures. We appreciate the opportunity to clarify the specific contributions and positioning of this work.

(1) Clarification of novelty beyond device-level directed logic

We would like to emphasize that the primary contribution of this work is not the introduction of a new physical mechanism for optical logic, but rather the system-level integration of directed logic units into a reconfigurable ALU architecture. While prior works have demonstrated individual logic gates or limited logic functions based on microring resonance tuning, these implementations are typically: task-specific, with fixed functionality, or limited to isolated logic operations without a unified computational framework. In contrast, our work introduces a modular architecture based on BLUs that enables: programmable composition of multiple logic and arithmetic functions, reuse of the same physical hardware for different operations, and a mapping between photonic logic primitives and standard digital ALU functionalities.

(2) On the “straightforward combination” of BLUs.

While the overall architecture can be viewed as a composition of basic logic units, the novelty lies in how these units are organized, interfaced, and programmed to realize a reconfigurable computing structure, rather than in the individual units themselves. Specifically, the proposed design: establishes a unified operational framework where

BLUs can be dynamically configured to perform both logic and arithmetic functions (e.g., AND, XOR, addition), enables consistent signal encoding and routing rules across different operations, and demonstrates functional completeness at the ALU level, which is not typically addressed in prior directed logic works.

(3) On the limited experimental demonstration (two-MRM case)

Current experimental implementation is limited to a reduced-scale configuration due to practical constraints in the available testing setup. In the revised manuscript and Supplementary Note 1, we have clarified this limitation and, more importantly, provided additional validation to ensure that it does not affect the correctness of the demonstrated functionality. Specifically, due to the limited availability of a four-channel AWG during high-speed testing, the experimental characterization of basic logic operations was performed using a two-channel AWG configuration. In this setup, certain combinational logic operations (e.g., AND between different basic logic outputs) are implemented through post-processing in the electrical domain. To verify the validity of this approach, we conducted additional comparative experiments using a four-channel AWG at lower data rates, where the required logic combinations are directly generated in the optical domain. The resulting waveforms are then systematically compared with those obtained by applying electrical-domain logic operations to the outputs measured from the two-channel configuration. As presented in Supplementary Figure S3, the waveforms obtained from these two approaches show strong agreement across multiple test cases and input conditions. In particular, the directly generated optical-domain logic results closely match those reconstructed via electrical-domain processing, demonstrating that the latter preserves the logical correctness and signal integrity of the operations. This comparison confirms that the use of electrical-domain post-processing does not introduce functional discrepancies, and can serve as a reliable surrogate for full multi-channel optical logic implementation under current experimental constraints.

Table R2 Comparison of representative optical logic implementations

Work	OU.	BLU	R.f.	ALU	R.f.	Fre. [Gbit/s]
[Z1]	DC	6	No	3	No	/
[Z2]	MRR	/	Yes	3	No	/
[Z3]	MRM	>3	Yes	/	No	/
[Z4]	MRM+PD	>6	Yes	/	No	9.4
[Z5]	MRM	3	Yes	1	No	No
[Z6]	MZI+MRR	>7	No	1	No	/
[Z7]	MZI	/	No	1	No	20
[Z8]	PLA	/	No	3	Yes	10
[Z9]	MZI	2	No	1	No	20
[Z10]	MZI	>5	Yes	/	No	25
This work	MRM	>7	Yes	3	Yes	20

Note: OU: operation unit, R.f.: Reconfigurable, Fre.: Frequency.

(4) On the definition of "reconfigurability"

Resonance tuning is a standard property of microring devices. In particular, we distinguish between: device-level tunability (resonance shift), and system-level reconfigurability, defined as the ability to program and switch between multiple computational functions within a unified architecture. Our use of "reconfigurability" refers to the latter, i.e., the functional programmability of the ALU, rather than the intrinsic tunability of individual devices. This clarification has been explicitly added to the manuscript.

(5) Positioning relative to recent progress

We have strengthened the discussion to more carefully position this work within the broader landscape of photonic computing. In this manuscript, we emphasize that this work represents a step toward bridging device-level optical logic and system-level programmable photonic computing architectures.

The comparative analysis in Table R2 further highlights that, while prior works

primarily focus on device-level demonstrations or fixed-function implementations, this work advances toward architecture-level programmability and ALU-oriented functionality.

In summary, we have revised the manuscript to more clearly and precisely articulate the novelty of this work as an architecture-level advancement, rather than a device-level innovation. We believe that these clarifications address the reviewer's concern and provide a more accurate and rigorous positioning of the contribution.

We sincerely thank the reviewer for prompting this important clarification, which has significantly improved the presentation and framing of our work. Furthermore, we add this section in Supplementary Note 1 as follow:

Supplementary Note 1: Advantages of MRM-based implementation for BLU and ALU operations

The proposed architecture leverages microring modulators (MRMs) as the fundamental building blocks for both basic logic units (BLUs) and higher-level arithmetic logic unit (ALU) functions. While resonance-based switching is a well-established mechanism, its use in the present work provides specific advantages at both the BLU and system levels, which are clarified below.

At the level of BLU implementation, MRMs enable a compact and efficient realization of logic operations through resonance-controlled transmission states. The transition between pass and block states is achieved via small refractive index perturbations, allowing logic operations to be directly mapped onto optical routing behavior. Compared to interferometric implementations, this resonance-based mechanism does not require precise phase matching over extended optical paths, which reduces sensitivity to phase noise and fabrication-induced imbalance. In addition, the localized nature of the resonant cavity allows BLUs to be implemented with a significantly reduced footprint, facilitating dense integration.

Another important characteristic at the BLU level is the high modulation efficiency enabled by resonance enhancement. Small electrical perturbations can induce large changes in optical transmission, which lowers the required driving voltage and

improves energy efficiency. This property is particularly beneficial when multiple BLUs are cascaded, as it helps maintain sufficient signal contrast without excessive electrical overhead.

Table S1 Comparison of representative optical logic implementations

Work	OU.	BLU	ALU	R.f.	Fre. [Gbit/s]
[S1]	DC	6	3	No	/
[S2]	MRR	/	3	Yes	/
[S3]	MRM	>3	/	Yes	/
[S4]	MRM+PD	>6	/	Yes	9.4
[S5]	MRM	3	1	Yes	/
[S6]	MZI+MRR	>7	1	No	/
[S7]	MZI	/	1	Yes	20
[S8]	PLA	/	3	Yes	10
[S9]	MZI	2	1	No	20
[S10]	MZI	>5	/	Yes	25
This work	MRM	>7	3	Yes	20

At the ALU level, the use of MRMs provides structural advantages in terms of modularity and reconfigurability. Since each BLU operates based on the same underlying resonance tuning mechanism, different logic and arithmetic functions can be realized through consistent control of electrical inputs, without modifying the physical interconnect topology. This enables a unified mapping between optical device states and digital logic operations, which is essential for constructing a reconfigurable ALU architecture.

Furthermore, the compact footprint of MRMs allows multiple BLUs to be integrated within a limited area, supporting the implementation of composite operations such as addition, subtraction, and comparison. This high integration density is critical for scaling toward more complex computational units, where spatial resource constraints become a dominant factor. As summarized in Table S1, compared with representative

prior works, the proposed architecture supports a larger number of BLUs and multiple ALU-level functions within a unified framework, while maintaining competitive operation speed. This comparison highlights that the advantage of the present work lies not in individual device performance, but in the level of architectural integration and functional programmability.

References

Z1. Ghosh RR, Dhawan A. Low loss, broadband, and non-volatile 'directed logic operations' using phase change materials in silicon photonics. ***IEEE J Quantum Electron*** 59, 1-13 (2023).

Z2. Xia Y, et al. A multifunctional optical computing system based on directed logic and micro-ring resonator. ***Opt Laser Technol*** 175, 110696 (2024).

Z3. Ying Z, Feng C, Zhao Z, Soref R, Pan D, Chen RT. Integrated multi-operand electro-optic logic gates for optical computing. ***Appl Phys Lett*** 115, (2019).

Z4. Soref R, De Leonardis F, Ying Z, Passaro VM, Chen RT. Silicon-based group-IV OEO devices for gain, logic, and wavelength conversion. ***ACS Photonics*** 7, 800-811 (2020).

Z5. Ruhul Fatin M, Gostimirovic D, Ye WN. Reconfigurable optical logic in silicon platform. ***Sci Rep*** 14, 5950 (2024).

Z6. Ghosh RR, Dhawan A. Integrated non-volatile plasmonic switches based on phase-change-materials and their application to plasmonic logic circuits. ***Sci Rep*** 11, 18811 (2021).

Z7. Feng C, Ying Z, Zhao Z, Gu J, Pan DZ, Chen RT. Toward high-speed and energy-efficient computing: a WDM-based scalable on-chip silicon integrated optical comparator. ***Laser Photonics Rev*** 15, 2000275 (2021).

Z8. Zhang W, et al. Large-scale optical programmable logic array for two-dimensional cellular automaton. ***Adv Photonics*** 6, 056007-056007 (2024).

Z9. Ying Z, et al. Electronic-photonic arithmetic logic unit for high-speed computing. ***Nat Commun*** 11, 2154 (2020).

Z10. Zhang W, et al. Photonic logic tensor computing beyond Tbit/s per core. ***Optica*** 12, 1252-1260 (2025).

Comment 2-2:

Application Depth: The demonstrated applications (2-bit grayscale image processing and ASCII encryption) are visually illustrative but technically rudimentary. Electronic

systems currently handle 16-bit to 32-bit precision with far higher reliability; a photonic ALU must demonstrate a clear path toward surpassing these electronic benchmarks in speed or efficiency, which this work does not yet show.

Author Response:

We appreciate the reviewer's comment on application depth. While electronic processors routinely achieve 16-bit to 32-bit precision, the strength of our photonic ALU lies in leveraging the intrinsic parallelism and high-throughput capability of optical systems. Even at 2-bit precision, our approach demonstrates functional correctness while exploiting wavelength-division multiplexing, spatial parallelism, and ultrafast electro-optic modulation to achieve effective computational throughput far exceeding that of conventional electronic ALUs in comparable serial processing. This demonstrates a clear pathway for scaling photonic computation toward higher-bandwidth and massively parallel tasks, highlighting the unique advantages of photonic architectures beyond what is possible with electronic systems.

(1) Fundamental difference in optimization targets: precision vs. throughput

Electronic ALUs are optimized for high-precision, sequential logic operations, typically operating at clock frequencies on the order of a few GHz (~1–3 GHz in conventional digital systems). [Y1, Y2] In contrast, photonic systems are inherently suited for high-throughput data processing, where multiple bits can be processed at symbol rates far exceeding electronic clock speeds. [Y3, Y4]

In this work, we experimentally demonstrate operation at 20 Gbit/s, which already exceeds typical electronic ALU clock rates when considering serial bit processing. More importantly, prior studies have reported photonic modulation and processing speeds exceeding 100–400 Gbit/s per channel[Y5, Y6], enabled by ultrafast electro-optic effects and wavelength-division multiplexing (WDM). These results indicate a clear pathway toward significantly higher aggregate throughput than electronic counterparts.

(2) Bandwidth and parallelism advantages (beyond single-channel comparison)

A key advantage of photonic systems is that computation can be performed not only in time but also in wavelength and spatial domains. While electronic ALUs scale primarily with clock frequency, photonic systems can leverage: wavelength-division

multiplexing (WDM), spatial parallelism, and analog amplitude encoding to achieve massively parallel processing. This allows the effective computational throughput to scale far beyond single-channel bit rates.

(3) Energy efficiency and data movement considerations

In modern computing systems, energy consumption is increasingly dominated by data movement rather than arithmetic operations. Photonic systems inherently reduce capacitive charging and interconnect losses, offering potential advantages in energy per bit, particularly for high-bandwidth data paths.

Recent studies have reported sub-fJ/bit modulation energy in microring-based devices [Y5, Y7], highlighting the potential for energy-efficient high-speed operation when integrated at scale.

(4) Role of this work: architectural foundation rather than application endpoint

The current demonstrations are limited in precision and application complexity. These examples are intended to validate the functional correctness and programmability of the photonic ALU architecture.

The key contribution of this work is to establish a reconfigurable architectural framework, which can be extended—through higher-speed modulators, multiplexing techniques, and improved device integration—toward higher-throughput and more complex computational tasks.

In summary, while electronic systems remain dominant for high-precision general-purpose computing, photonic ALUs offer a complementary pathway optimized for ultra-high-speed, high-bandwidth, and parallel processing scenarios. We have clarified this positioning in the revised manuscript and added relevant references to better contextualize the advantages and future potential of the proposed approach. Furthermore, we add this section in Supplementary Note 10 as follow:

Supplementary Note 10. Advantages of photonic ALUs for high-throughput computation

Photonic ALUs differ fundamentally from conventional electronic ALUs in their optimization targets. Electronic ALUs are primarily designed for high-precision sequential computation, where performance scaling is typically constrained by clock

frequency, interconnect delay, and electrical power dissipation[S12, S13]. Modern electronic processors routinely support 16-bit to 32-bit precision arithmetic with high reliability, but their throughput scaling increasingly faces limitations imposed by RC delays, memory bandwidth, and data movement overhead.

In contrast, photonic ALUs are inherently suited for high-throughput parallel computation by exploiting the unique degrees of freedom available in optical systems[S14, S15]. In addition to temporal processing, optical computation can be simultaneously scaled through WDM, spatial parallelism, and high-speed electro-optic modulation. This enables multiple logic operations to be executed concurrently without relying solely on increased clock frequency or extensive hardware replication.

In this work, the proposed photonic ALU experimentally demonstrates operation at 20 Gbit/s. More importantly, prior studies have shown that substantially higher operating speeds are feasible in integrated photonic platforms. For example, in S16, while recent microring-based devices have reported modulation speeds exceeding 400 Gb/s per wavelength[S16, S17]. These advances provide a clear pathway toward significantly higher aggregate computational throughput in future photonic ALU systems.

Beyond raw throughput, photonic ALUs also offer architectural advantages in bandwidth-intensive workloads. Since optical signals propagate without capacitive charging losses and support direct parallel transmission across multiple channels, photonic architectures can significantly reduce interconnect bottlenecks and data movement overhead. As discussed in S13 and S15, these characteristics become increasingly important as communication overhead dominates modern computing systems.

Therefore, while electronic ALUs remain advantageous for high-precision general-purpose computing, photonic ALUs provide a complementary computing paradigm optimized for ultra-high-speed, high-bandwidth, and massively parallel information processing. The proposed architecture establishes a scalable foundation for extending photonic computation toward higher precision and more complex computational tasks.

References

Y1. Waldrop MM. More than Moore. ***Nature*** 530, 144-148 (2016).

Y2. Tucker RS, Hinton K. Energy consumption and energy density in optical and electronic signal processing. ***IEEE Photonics J*** 3, 821-833 (2011).

Y3. Ying Z, et al. Electronic-photonic arithmetic logic unit for high-speed computing. ***Nat Commun*** 11, 2154 (2020).

Y4. McMahon PL. The physics of optical computing. ***Nat Rev Phys*** 5, 717-734 (2023).

Y5. Hu F, et al. 400-Gbps/λ Ultrafast Silicon Microring Modulator for Scalable Optical Compute Interconnects. ***Laser Photonics Rev***, e02840 (2025).

Y6. Wang X, et al. A 400 Gbps Microring Modulator with 4.2 THz FSR Using Optimized Bezier Bend in a 300 mm CMOS Platform. ***ACS Photonics***, (2026).

Y7. Wang X, et al. 290-Gbps Silicon Photonic Microring Modulator with 83-aJ/bit Ultra-Low Power Efficiency. **Optical Fiber Communications Conference (OFC)**, (2025).

Comment 2-3:

Scalability: The paper demonstrates 1-bit and 2-bit operation. However, there is no clear path to scaling to a multi-bit system without an exponential increase in circuit size. The authors should calculate the number of MRRs required and the potential performance for an N-bit ALU (where = 3, 4, 8...).

Author Response:

We thank the reviewer for this important comment regarding scalability. We agree that a clear scaling pathway toward multi-bit ALU implementations is necessary.

In the revised manuscript and Supplementary Note 8, we have added a dedicated analysis on scalability, including quantitative estimates of the required number of MRMs and the corresponding performance for multi-bit cases (e.g., N=3,4,8).

In this work, the demonstrated 2-bit ALU is designed as a parallel prototype, which represents the minimal functional unit for arithmetic operations (e.g., addition and subtraction). To address scalability, we clarify that higher-bit operations can be realized using a time-domain iterative computation strategy, rather than purely spatial scaling.

(1) Multi-bit adder implementation via time-domain multiplexing

Taking the adder as an example, the input signals can be decomposed into three

components: the initial carry (C_0), and two operand sequences ($A = A_iA_{i-1}...A_3A_2A_1$) and ($B = B_iB_{i-1}...B_3B_2B_1$). Through time-division multiplexing (TDM), the data streams of A and B are encoded into temporal sequences and sequentially loaded onto the corresponding MRMs.

As the expression in Table T1,, the signals A_i, B_i and C_{i-1} are applied to the MRM-based logic unit, generating the sum Si and carry C_i. The carry signal C_i. is optically split into multiple paths, where a portion is fed back as the input carry C_{i-1} for the next computation stage via a conventional optical–electrical–optical (OEO) loop, while the remaining portion is used for output acquisition. In this way, a sequence of carry bits $C_1C_2...C_i$ is generated iteratively, which can be seen in the Figure R3a.

For addition, only the most significant carry bit is required as the final output, while all sum bits Si are retained. This approach allows the realization of higher-bit adders with constant hardware complexity, where the scalability is primarily limited by the efficiency of OEO conversion and the minimum signal power required for reliable operation.

Table R1 2-bit ALUs' expression

Adder	C_i	$C_{i-1}(A_i \oplus B_i) + (A_i \bullet B_i)$
	S_i	$A_i \oplus B_i \oplus C_{i-1}$
Subtractor	C_i	$C_{i-1}(\overline{A_i \oplus B_i}) + (\overline{A_i} \bullet B_i)$
	D_i	$A_i \oplus B_i \oplus C_{i-1}$
Comparator	$F_{A>B}$	$A_2 \bullet \overline{B_2} + (A_2 \odot B_2)(A_1 \bullet \overline{B_1})$
	$F_{A<B}$	$\overline{A_2} \bullet B_2 + (A_2 \odot B_2)(\overline{A_1} \bullet B_1)$
	$F_{A=B}$	$(A_1 \odot B_1) + (A_2 \odot B_2)$

(2) Subtractor scalability

The subtractor follows a similar structure to the adder, as the expressions in Table R1 share the same logical form. By appropriately reconfiguring the MRM states and input ports, the same time-domain iterative scheme can be applied to realize multi-bit

subtraction without additional hardware overhead, which can be seen in the Figure R3b.

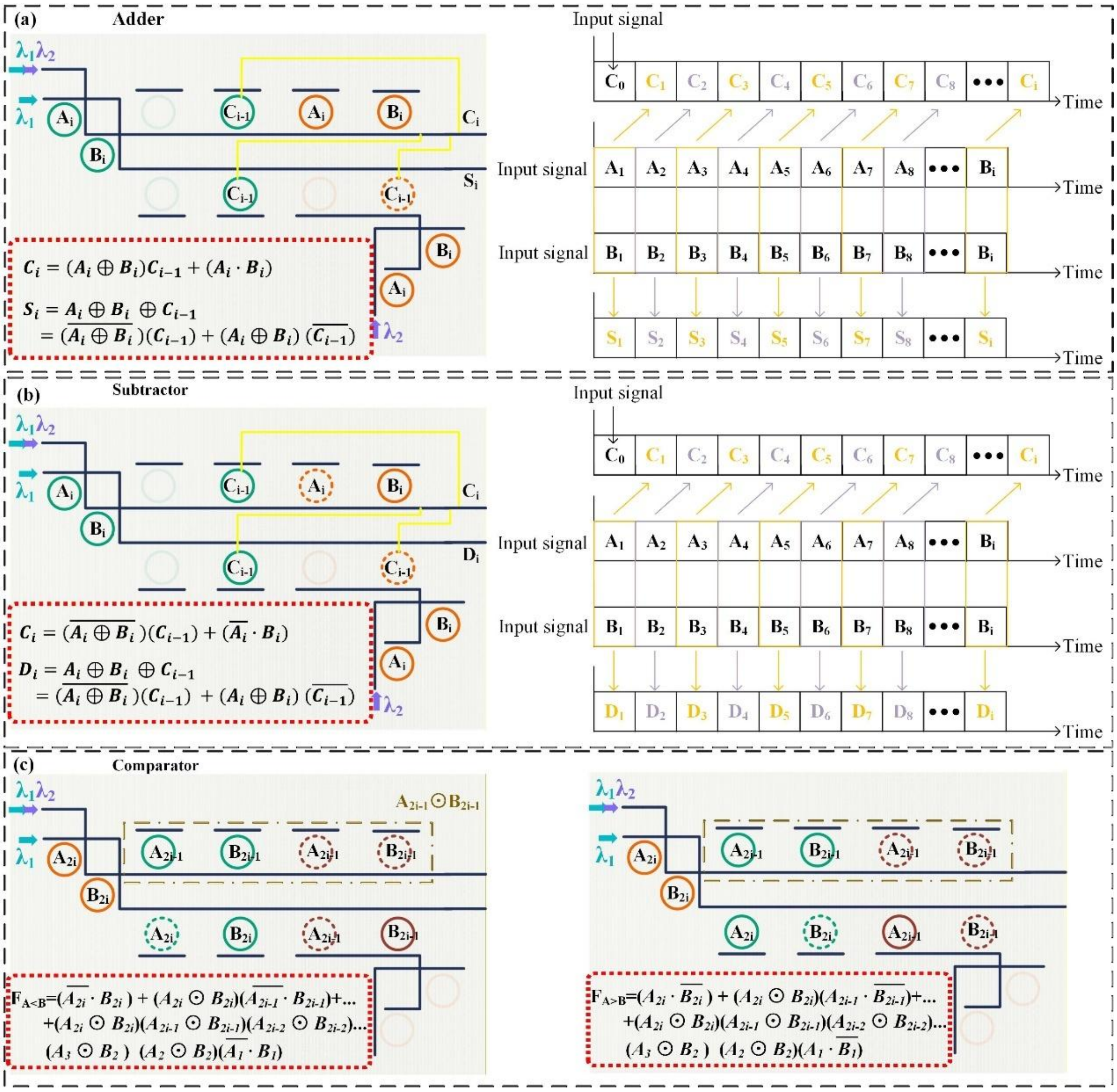

Figure R3. Schemes for bit-width extension of arithmetic units. (a) Bit-width extension scheme for full adders. (b) Bit-width extension scheme for full subtractors. (c) Bit-width extension scheme for comparators.

(3) Comparator scalability

In contrast to adders and subtractors, comparators exhibit a different scaling behavior. The number of required BLU operations increases with bit width due to the need for multi-bit comparison logic. Specifically, a 2-bit comparator requires 3 BLU operations, a 3-bit comparator requires 6, and a 4-bit comparator requires 10. Extending this trend, an 8-bit comparator would require 36 BLU operations. Unlike arithmetic units, the comparator logic does not readily reduce to a simple iterative structure. In our architecture, the BLU operations can still be executed in the optical domain, while the

final decision logic can be completed in the electrical domain to maintain system efficiency.

(4) Trade-off between scalability and throughput

We emphasize that the proposed time-domain approach enables multi-bit operation without exponential growth in hardware resources, effectively avoiding the scalability limitations of purely spatial implementations. However, this comes with a trade-off: as the bit width increases, the computation transitions from parallel to partially serial operation, leading to a reduction in overall throughput under a fixed modulation rate. Nevertheless, by leveraging MRMs with a higher modulation rate, we can maintain the overall computational throughput while enabling ALU computation with a significantly wider bit width.

In summary, while a native spatial extension would lead to a rapid increase in circuit size, the proposed architecture provides a feasible and resource-efficient pathway toward multi-bit ALUs through time-domain multiplexing and iterative computation. This approach transforms the scaling behavior from spatial resource growth to temporal processing overhead. As a result, the number of required MRMs can remain approximately constant for arithmetic operations (adder/subtractor), while the effective computation rate scales inversely with bit width.

We have revised the manuscript to explicitly include this scalability analysis, along with quantitative examples of BLU usage for different bit widths. We believe this clarification addresses the reviewer's concern and provides a more complete picture of the scalability and practical design trade-offs of the proposed architecture. Furthermore, we add this section in discussion the manuscript and Supplementary Note 9 as follow:

"Such improvements would leverage higher electro-optic modulation speeds to elevate computational density while maintaining strict power constraints. Second, while the current device successfully implements reconfigurable 2-bit adders, subtractors, and comparators by cascaded basic logic units, we have validated a time-domain iterative computation strategy to enable multi-bit ALU operation without proportional hardware scaling, with full scalability analysis and mitigation strategies for large-scale integration challenges detailed in Supplementary Note 9. Future work

could further refine this cascading and iterative processing methodology to support more diverse and computationally complex applications without sacrificing reconfigurability or energy efficiency."

Supplementary Note 9: Scalability toward multi-bit photonic ALUs

To evaluate the potential of the proposed architecture for large-scale photonic computing, we analyze its scalability toward higher-bit ALUs, and discuss the associated challenges and feasible mitigation strategies.

A direct spatial extension of the demonstrated 2-bit architecture would require a proportional increase in the number of MRMs, leading to rapidly growing circuit complexity and footprint. To address this limitation, the proposed architecture adopts a time-domain iterative computation strategy, which enables multi-bit operation without proportional hardware scaling.

Taking the adder as an example, the input operands $A{\cdot}B$ are decomposed into bit sequences and encoded into time-division multiplexed (TDM) signals. At each time slot, the corresponding bits A_i, B_i, and the carry input C_{i-1} are applied to the MRM-based logic unit, generating the sum S_i and carry C_i. The carry signal is partially fed back through an optical–electrical–optical (OEO) loop to serve as the input for the next time step, enabling iterative computation across multiple bit positions. Under this scheme, multi-bit addition and subtraction can be realized with approximately constant hardware resources, where scalability is primarily determined by temporal processing depth rather than spatial circuit size.

The scalability characteristics differ for arithmetic and comparison operations. For addition and subtraction, the iterative structure allows efficient reuse of the same set of BLUs. In contrast, comparator functions require an increasing number of BLU operations with bit width due to multi-bit comparison logic. For example, a 2-bit comparator requires 3 BLUs, while 4-bit and 8-bit implementations require 10 and 36 BLUs, respectively. To maintain system efficiency, the architecture performs core comparison operations in the optical domain, while the final decision logic can be completed in the electrical domain.

Scaling to larger systems introduces several practical challenges associated with cascading photonic logic units. Optical signal attenuation accumulates due to waveguide propagation loss, coupling loss, and device insertion loss, which can degrade signal integrity in deep cascaded configurations. This can be mitigated through low-loss waveguide design, optimized coupling structures, and, when necessary, the incorporation of optical amplification stages or regeneration mechanisms. Crosstalk and spectral interference may arise from dense integration of resonant devices, particularly under closely spaced wavelength channels. These effects can be reduced through careful resonance engineering, wavelength allocation, and layout-level isolation strategies.

Signal synchronization is another critical consideration in multi-channel operation. However, this requirement is consistent with standard practices in high-speed electronic–photonic integrated systems and can be addressed through co-design of driver electronics and photonic devices, matched interconnect routing, and localized clock distribution. In addition, the use of time-domain multiplexing reduces the number of simultaneously active channels, thereby relaxing synchronization constraints in large-scale implementations.

The adoption of time-domain iterative processing introduces a fundamental trade-off between scalability and throughput. As the bit width increases, the computation becomes partially serialized, leading to a reduction in effective throughput under a fixed modulation rate. Nevertheless, given the high intrinsic speed of photonic devices, the resulting performance remains competitive for high-bandwidth applications.

Overall, the proposed architecture transforms the scaling behaviour from spatial resource growth to temporal processing overhead, providing a feasible pathway toward multi-bit photonic ALUs. Combined with advances in photonic integration and system-level co-design, this approach offers a scalable foundation for future large-scale photonic computing systems.

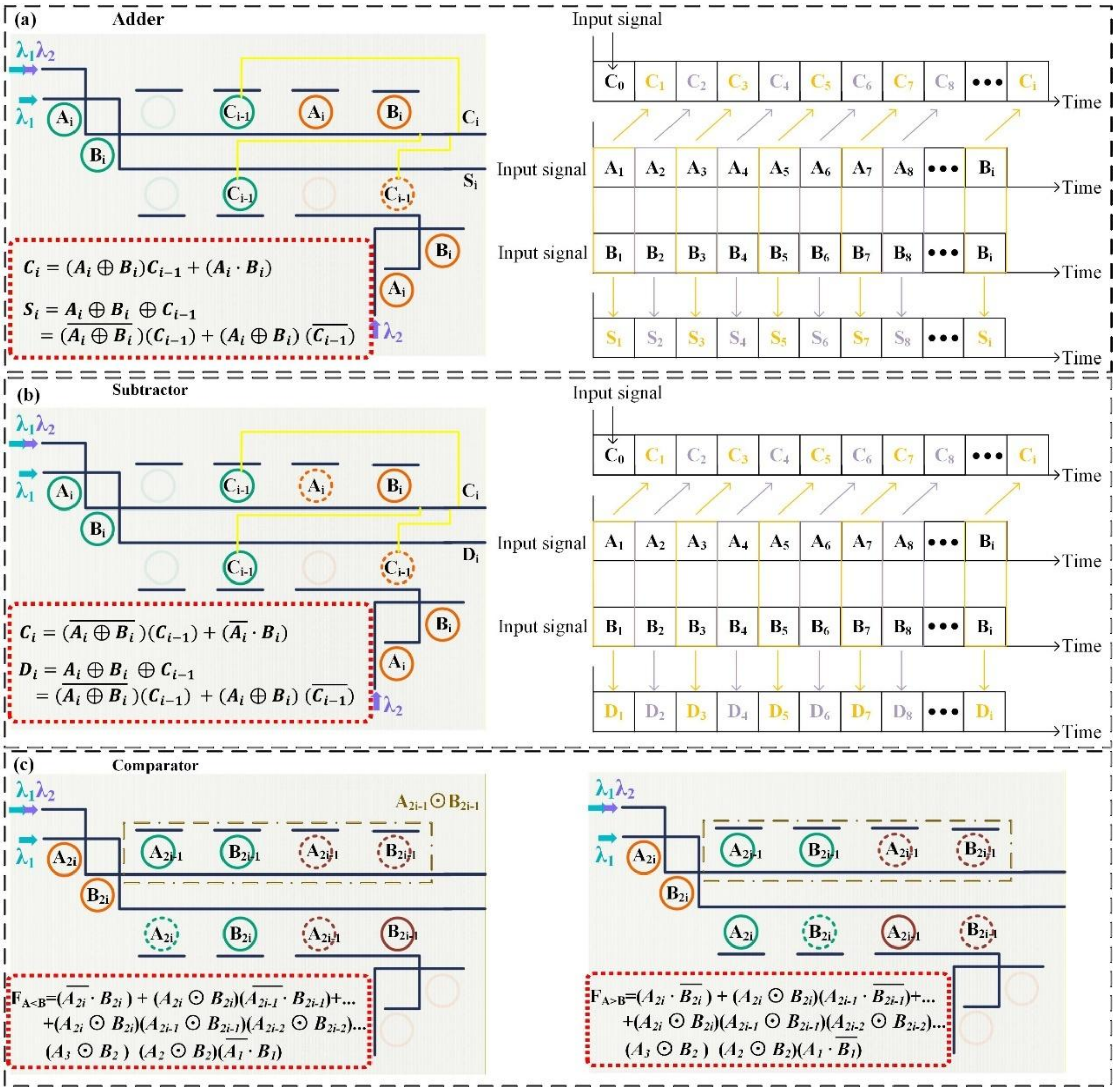

Figure S10. Schemes for bit-width extension of arithmetic units. (a) Bit-width extension scheme for full adders. (b) Bit-width extension scheme for full subtractors. (c) Bit-width extension scheme for comparators.

Comment 2-4:

Signal Synchronization: Each electrical driving signal is applied to multiple MRRs simultaneously. Synchronizing these signals in a high-speed integrated environment is extremely difficult and significantly increases power consumption. The authors must address this practical implementation challenge.

Author Response:

We thank the reviewer for this important comment regarding signal synchronization in multi-channel MRM-based systems. We agree that distributing and synchronizing high-speed electrical signals across multiple devices is a critical practical

challenge.

However, we would like to clarify that this issue has already been systematically addressed in recent large-scale photonic computing systems, and therefore does not represent a fundamental limitation of the proposed architecture.

(1) Evidence from large-scale photonic systems

Recent large-scale demonstrations have shown that synchronized multi-channel electrical–optical operation is feasible even at significantly larger scales than considered in this work.

For example, in the Ref. X1, the researches reports a photonic computing system integrating over 16,000 photonic components, where photonic circuits are co-integrated with electronic driver and control circuits using advanced packaging. The system operates with a 1 GHz clock and nanosecond-level latency, relying on coordinated multi-channel modulation and detection.

Importantly, this work explicitly demonstrates that large-scale photonic systems can achieve synchronized operation across thousands of devices, enabled by electronic–photonic co-design and integrated driver circuits.

Similarly, in Ref. X2, the authors demonstrates a photonic processor capable of executing complex AI models (e.g., ResNet and BERT), which inherently requires coordinated control of a large number of optical computing elements. This further confirms that synchronization is a solved system-level challenge in state-of-the-art photonic computing platforms, rather than a limiting factor of specific device architectures.

(2) Nature of the synchronization challenge

We emphasize that synchronization in our system is conceptually equivalent to clock distribution in electronic processors and driver synchronization in high-speed optical transceivers, where multi-channel alignment at tens of GHz is routinely achieved.

Moreover, the operating data rate in this work is well within the regime of existing silicon photonic modulators and driver circuits, which have demonstrated synchronized operation at 25-100+ Gbit/s per channel in prior studies[X3,X4].

(3) Power consumption considerations

The signal distribution introduces additional power overhead. However, as also discussed in an integrated large-scale photonic accelerator with ultralow latency, the dominant system-level advantages of photonic computing arise from: reduced data movement, parallel optical signal processing, and avoidance of resistive–capacitive (RC) losses [X1, X4].

As a result, the overall energy efficiency is not limited by synchronization overhead, but is instead governed by the modulation and system architecture.

(4) Implications for this work

In comparison to the above large-scale systems, the number of simultaneously driven MRMs in this work is significantly smaller. Therefore, the synchronization requirement is substantially less demanding and can be addressed using established electronic–photonic co-design techniques.

In addition, the proposed architecture can further mitigate synchronization complexity through: localized signal distribution, modular BLU-based design, and time-domain multiplexing strategies for scalable operation.

In summary, while signal synchronization is indeed an important practical consideration, recent large-scale photonic computing systems published in top-tier journals demonstrate that synchronized multi-channel operation is already achievable at much larger scales. Therefore, it does not constitute a fundamental limitation of the proposed architecture.

References

X1. Hua S, et al. An integrated large-scale photonic accelerator with ultralow latency. ***Nature*** 640, 361-367 (2025).

X2. Ahmed SR, et al. Universal photonic artificial intelligence acceleration. ***Nature*** 640, 368-374 (2025).

X3. Yan P, et al. Integrated optical transceivers: architectures, key technologies, and applications. ***Opto-Electronic Technology***, 2026.

X4. Liu, H, et al. "A 4×112-Gb/s, 3.51-pJ/Bit Monolithically Integrated Silicon-Photonic Transceiver for High-Density Co-Packaged Optics." ***2025 Symposium on VLSI Technology and Circuits (VLSI***

Technology and Circuits). IEEE, 2025.
X5. Ivonne Bente, et al. The potential of multidimensional photonic computing. ***Nat Rev Phys*** 7, 439–450 (2025).

Comment 2-5:

Full-Circuit Verification: I understand high-speed hardware is limited, but can the authors provide DC performance data for the entire 12-MRR circuit? Demonstrating only two-ring cases is insufficient to prove the full system-level integration.

Author Response:

We thank the reviewer for this important and constructive comment. In the revised Supplementary Note 5, we have supplemented additional DC characterization results involving multiple MRMs and wavelengths to better reflect the functionality of the full circuit. Specifically, we provide the following experimental validations:

(1) Two wavelength channels with independent logic operations.

We experimentally demonstrate that two distinct wavelength channels can simultaneously perform two different logic operations, verifying the capability of wavelength-division multiplexing for parallel computation, as in Figure R4.

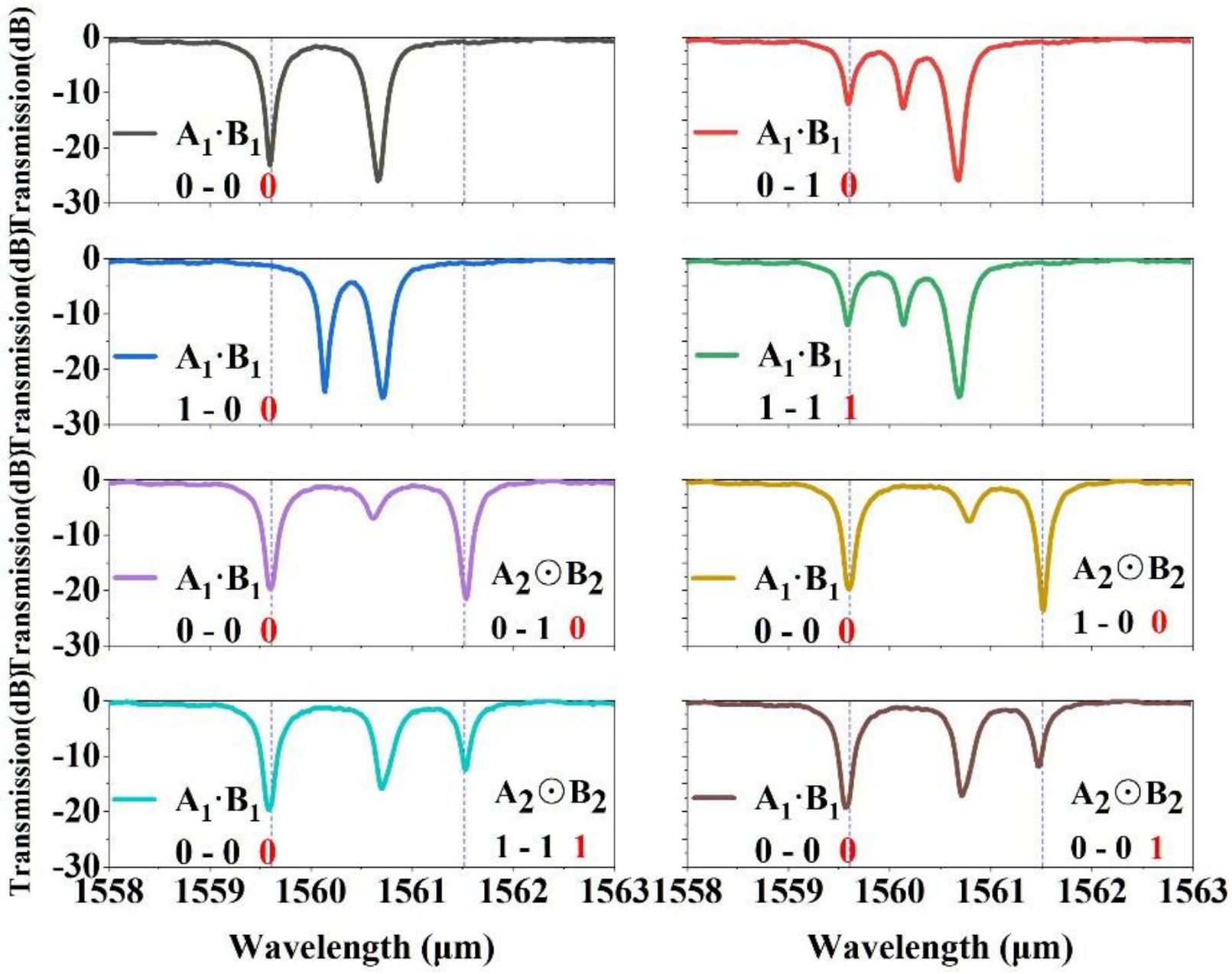

Figure R4. Two wavelength channels with independent logic operations

The results show all the operations are correct and the BLUs can process at two different wavelengths.

(2) Single-wavelength BLU operation.

We perform AND operations within a single wavelength channel using the BLU and experimentally confirm the correctness of the logic outputs, validating the fundamental operation of the building block, as in Figure R5.

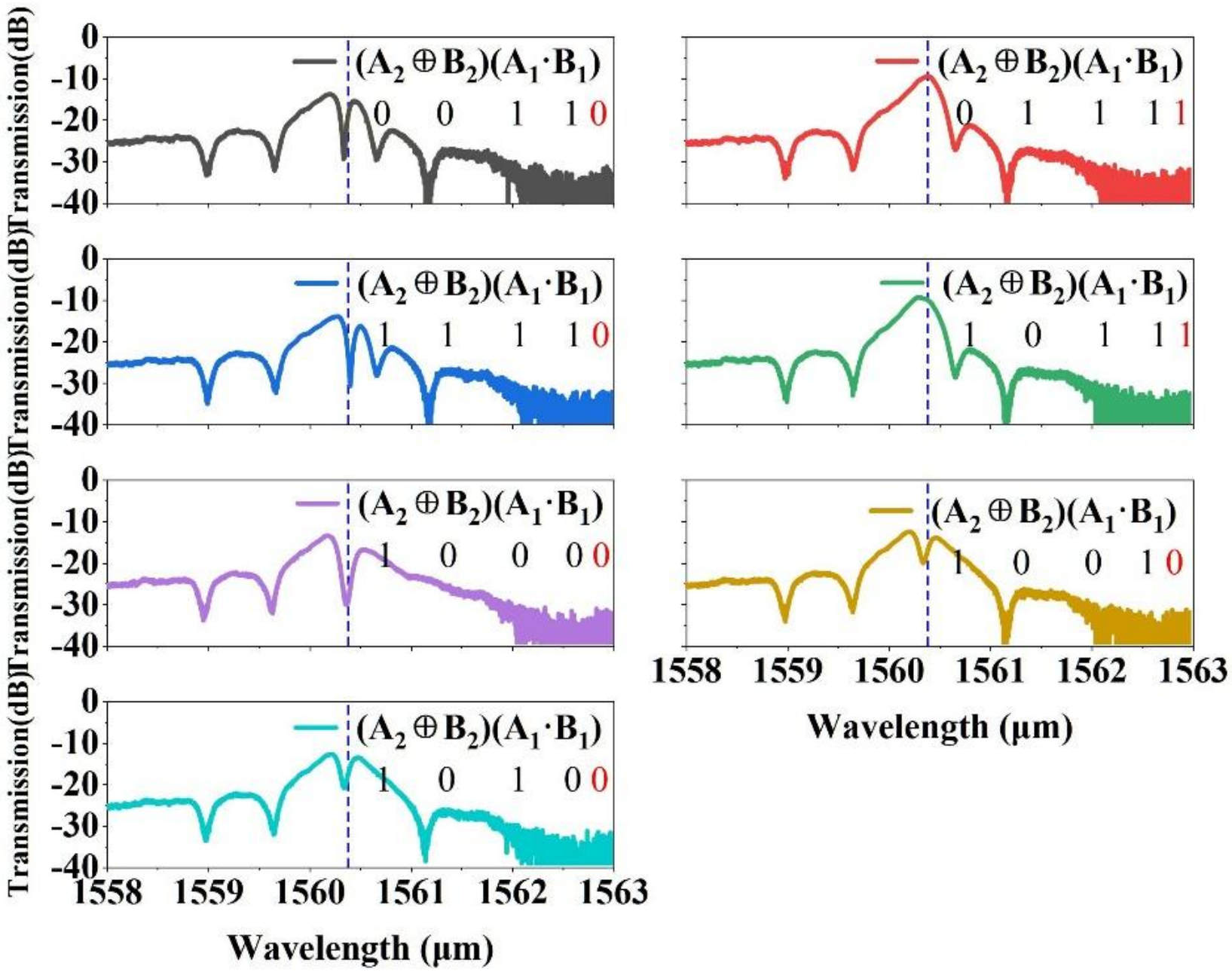

Figure R5. Spectra of two BLUs modulated on a single wavelength.

The results show all the operations are correct and the BLUs can process at single wavelengths for the AND operation of BLUs.

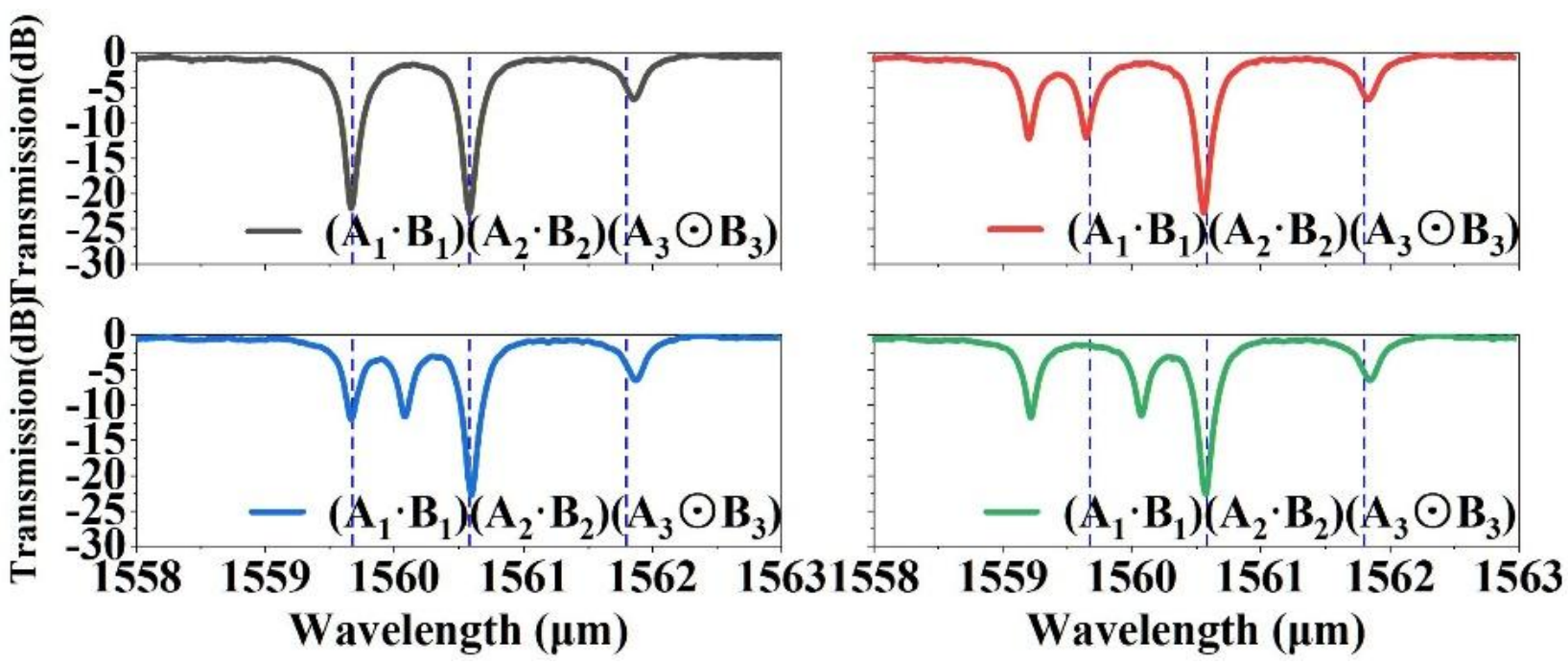

Figure R6. Spectra of three distinct BLUs at three wavelengths.

(3) Three-wavelength multi-operation demonstration.

We extend the experiment to three wavelength channels, where three different logic operations are executed in parallel. The results confirm that multiple BLUs can operate concurrently without functional interference, as in Figure R6.

(4) Independent dual-wavelength logic operations.

We further demonstrate that two separate wavelength pairs can independently perform two different logic operations, highlighting the flexibility and parallelism of the architecture in a multi-channel configuration, as in Figure R7.

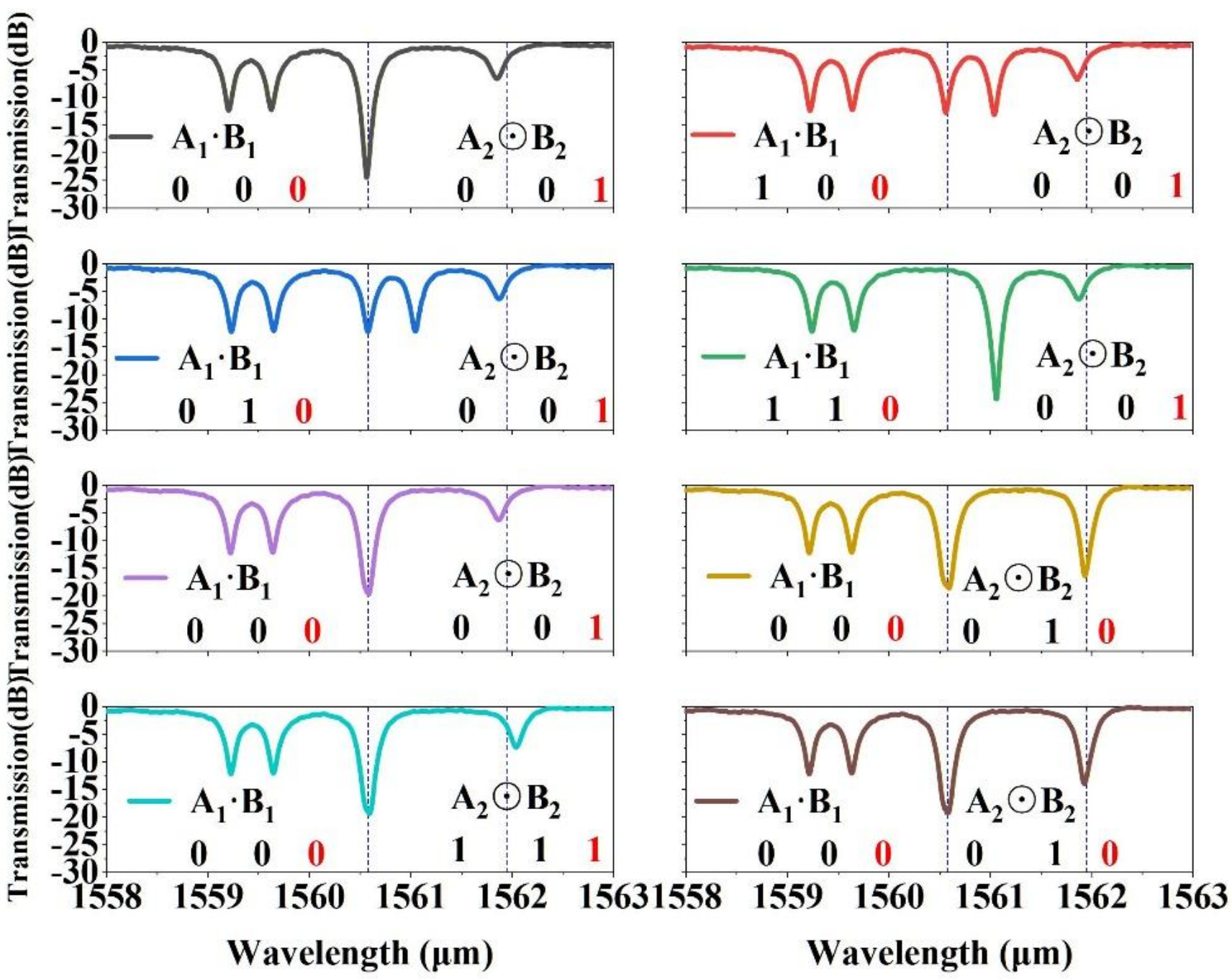

Figure R7. Spectra of two distinct BLUs at other two wavelengths

These results collectively verify that, although the high-speed measurements are performed on a reduced configuration, the multi-wavelength and multi-MRM DC operation confirms the correct functionality of the larger-scale integrated system.

We have added these results and corresponding discussions in the revised manuscript to better support the system-level integration capability. We believe these additions address the reviewer's concern and provide stronger evidence for the scalability of the proposed architecture. Furthermore, we add this section in Supplementary Note 5 as follow:

Supplementary Note 5: System-level DC characterization of multi-MRM circuits

To further validate the system-level integration capability of the proposed architecture beyond the two-MRM high-speed demonstration, we perform comprehensive DC characterization involving multiple MRMs and wavelength channels. These measurements are designed to verify the functionality of cascaded BLUs under multi-channel operation and to assess the feasibility of scaling toward larger circuits.

First, we demonstrate parallel logic operations using two independent wavelength channels, as shown in Fig. S4. In this configuration, two distinct wavelengths are simultaneously injected into the circuit, each performing a different logic operation. The experimental results confirm that both operations are correctly executed without mutual interference, indicating that the BLUs can operate independently across different wavelength channels and supporting the feasibility of wavelength-division multiplexing for parallel computation.

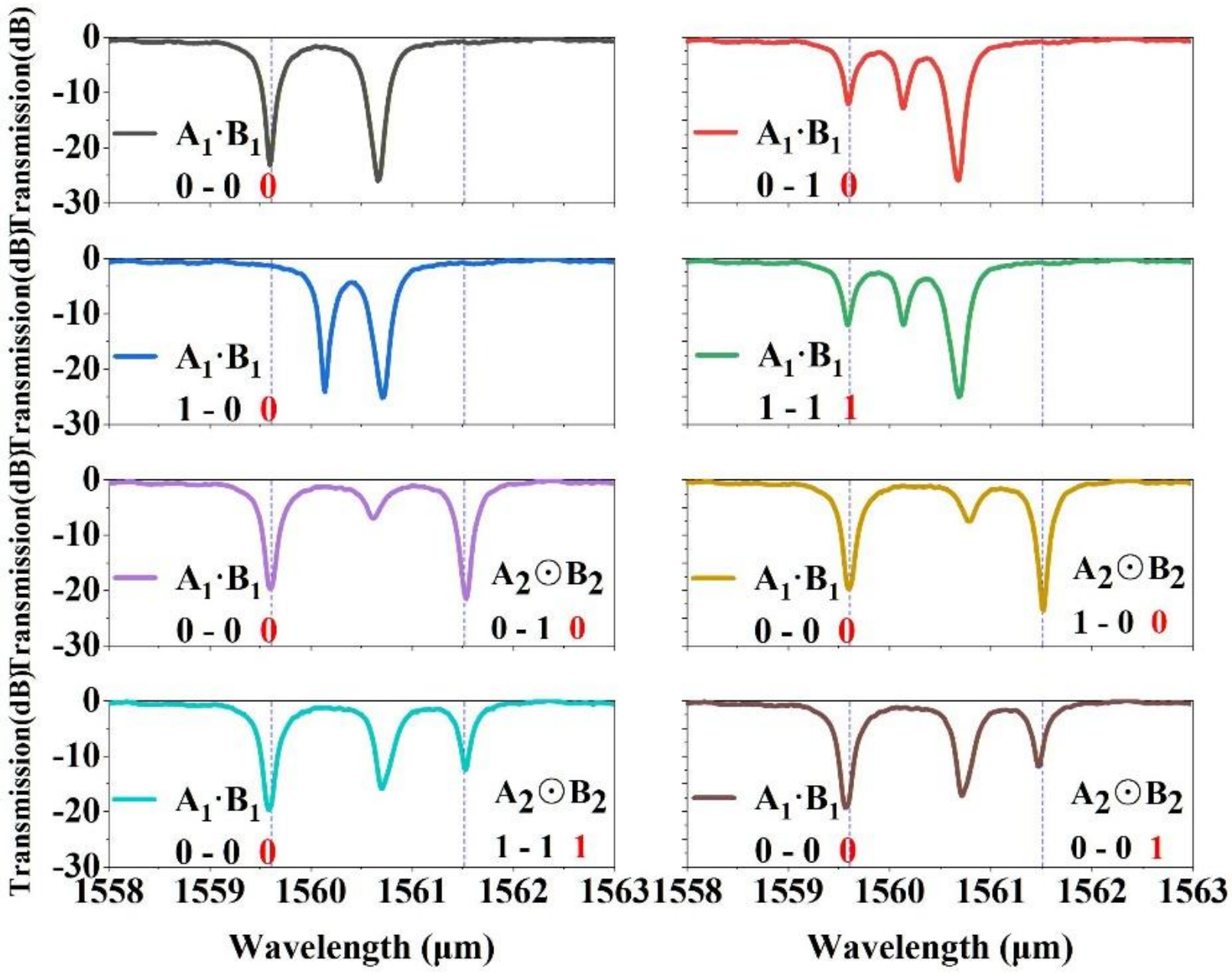

Figure S4. Two wavelength channels with independent logic operations

To validate the fundamental functionality of the BLU at the single-channel level, we perform logic operations within a single wavelength channel. As presented in Fig. S5, AND operations are implemented using the BLU, and the measured outputs match the expected logic results. This confirms the correctness and robustness of the basic logic unit under single-wavelength operation.

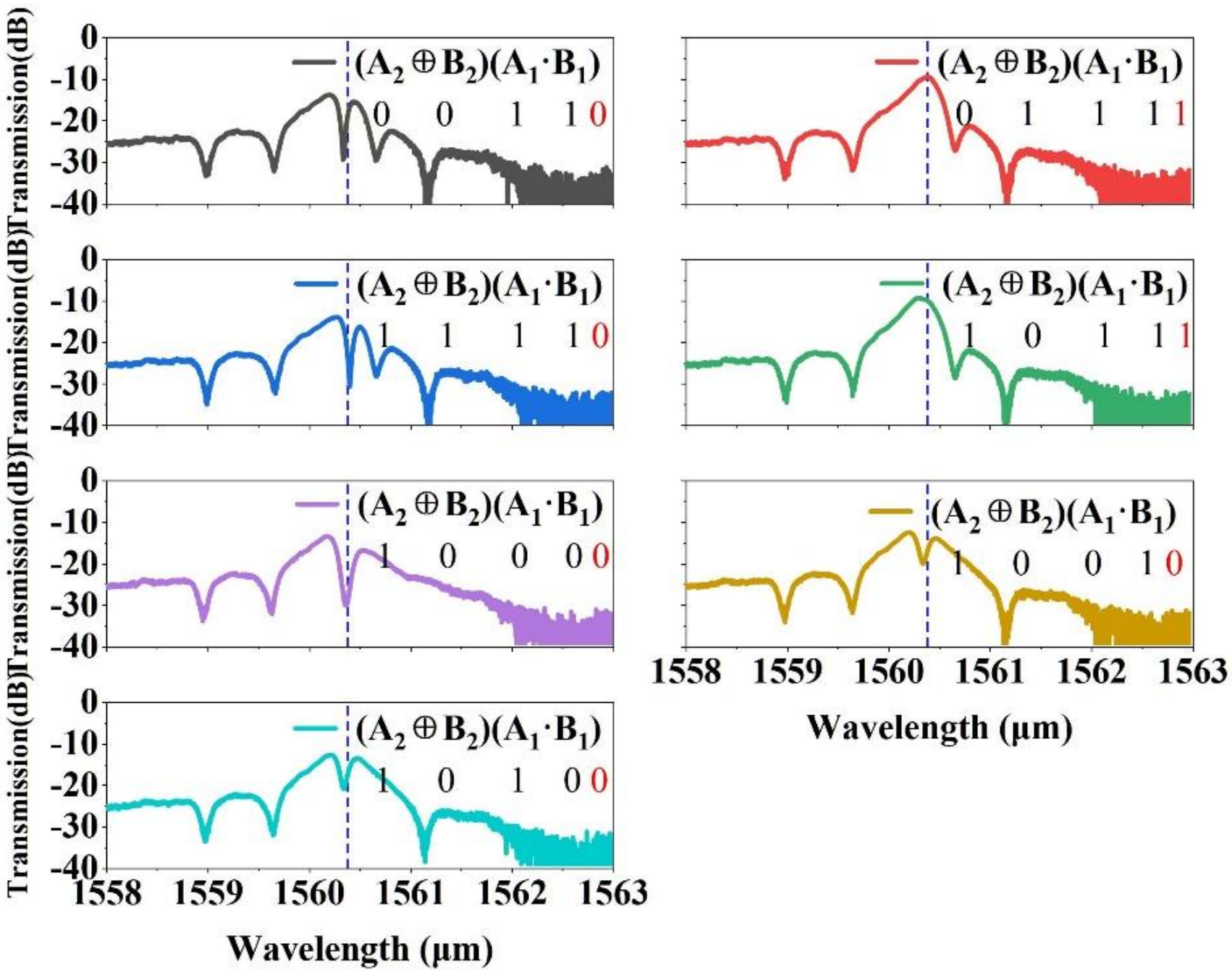

Figure S5. Spectra of two BLUs modulated on a single wavelength.

We further extend the demonstration to three wavelength channels, where three distinct logic operations are executed simultaneously, as shown in Fig. S6. The results show that all operations are correctly performed, with no observable functional degradation or interference between channels. This experiment verifies that multiple BLUs can operate concurrently in a multi-wavelength configuration, providing further evidence of the scalability of the architecture.

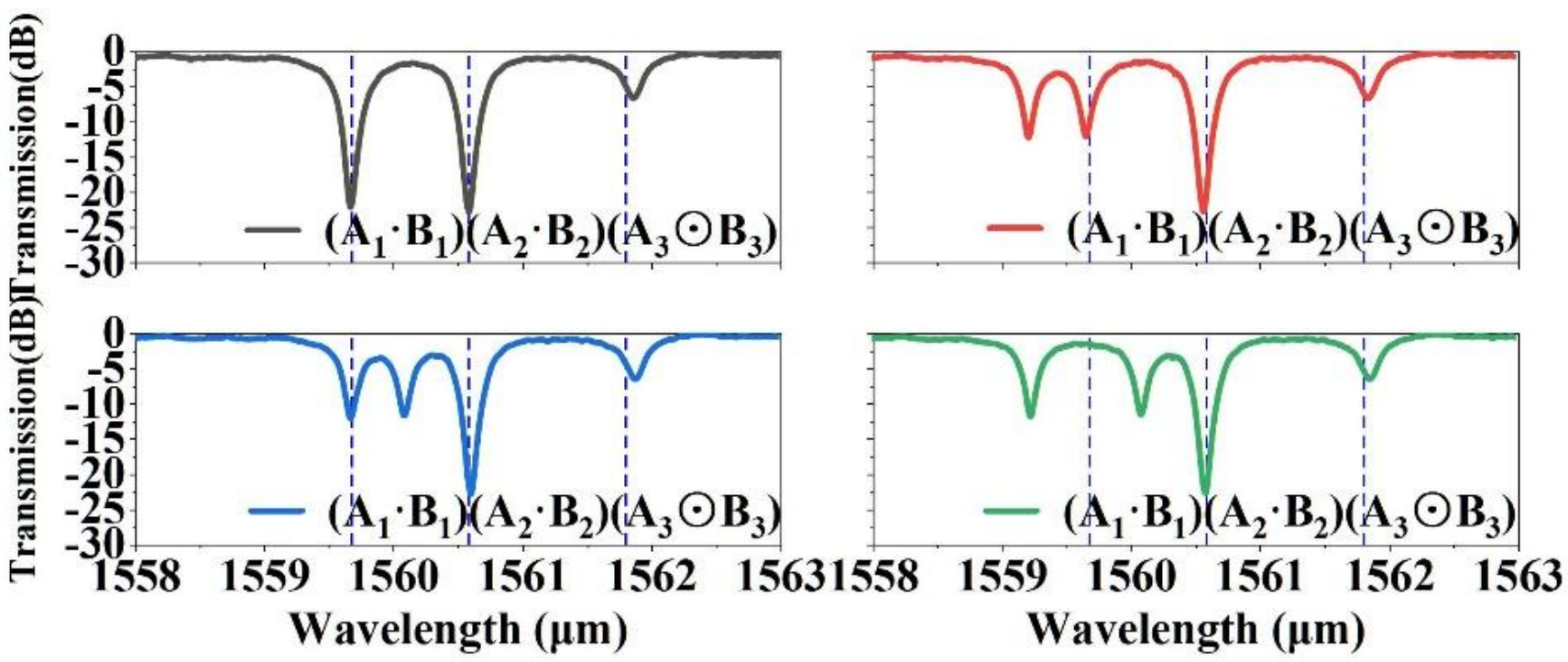

Figure S6. Spectra of three distinct BLUs at three wavelengths.

In addition, we investigate a more complex multi-channel scenario involving independent wavelength pairs. As illustrated in Fig. S7, two separate wavelength pairs are used to perform two different logic operations independently within the same circuit. The experimental results confirm that these operations can be executed simultaneously

without cross-channel interference, demonstrating the flexibility and configurability of the system in handling multiple logic functions in parallel.

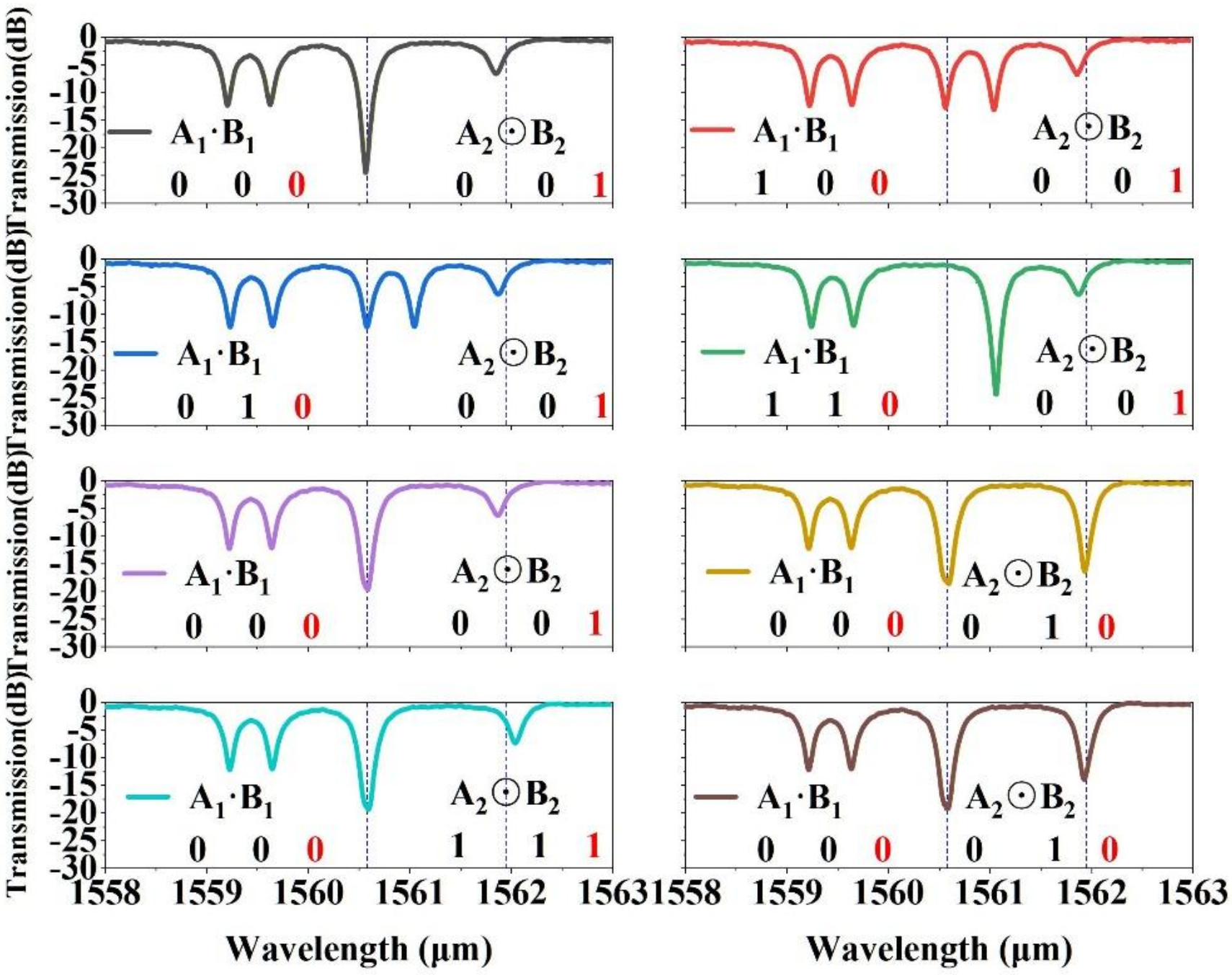

Figure S7. Spectra of two distinct BLUs at other two wavelengths

Collectively, these results show that, although high-speed measurements are conducted on a reduced two-MRM configuration, the DC characterization of multi-wavelength and multi-MRM operation provides strong evidence for correct functionality at the system level. The demonstrated capability of parallel, independent logic processing across multiple wavelength channels supports the scalability of the proposed architecture toward larger photonic computing systems.

Comment 2-6:

Reconfigurability Claims: The authors claim functions can be switched without physical redesign. However, the circuits in Figure 1d and Figure 4a appear different. Please clarify if these are the same physical device; otherwise, the claim of "reconfigurability" is misleading.

Author Response:

We thank the reviewer for this careful observation and for pointing out the potential ambiguity regarding the reconfigurability claim.

We would like to clarify that Figure 1d and Figure 4a correspond to the same

underlying physical architecture, and no physical redesign of the core device is required to switch between different functions. The apparent difference between the two figures arises from the way the layouts are presented for explanatory purposes.

Specifically, Figure 1d illustrates the overall ALU architecture and signal flow at the system level, while Figure 4a is intentionally redrawn to explicitly show the generation and configuration of individual BLUs. In order to clearly highlight how each BLU is formed and how different logic functions are realized, the waveguide layout in Figure 4a is rearranged and simplified for readability. However, this modification is purely schematic and illustrative, rather than representing a different fabricated structure.

In the actual implementation, all logic and arithmetic functions are realized on the same physical device by dynamically adjusting: the electrical driving signals applied to the MRMs, and the corresponding input/output configurations. No structural changes, re-fabrication, or physical re-routing of waveguides are required. Therefore, the term "reconfigurability" in this work refers to functional programmability within a fixed hardware architecture, rather than changes in physical layout.

We thank the reviewer for highlighting this issue, which has helped us improve the clarity and precision of the presentation.

Comment 2-7:

Minor concerns: Figure 2a: Add a scale bar and clearly mark all optical paths to clarify the logic flow.

Author Response:

We thank the reviewer for this helpful suggestion.

In the revised manuscript, we have updated Figure 2a by adding a scale bar and clearly marking all optical paths to improve the clarity of the device layout and logic flow. These modifications make the signal propagation and functional regions more intuitive and easier to follow.

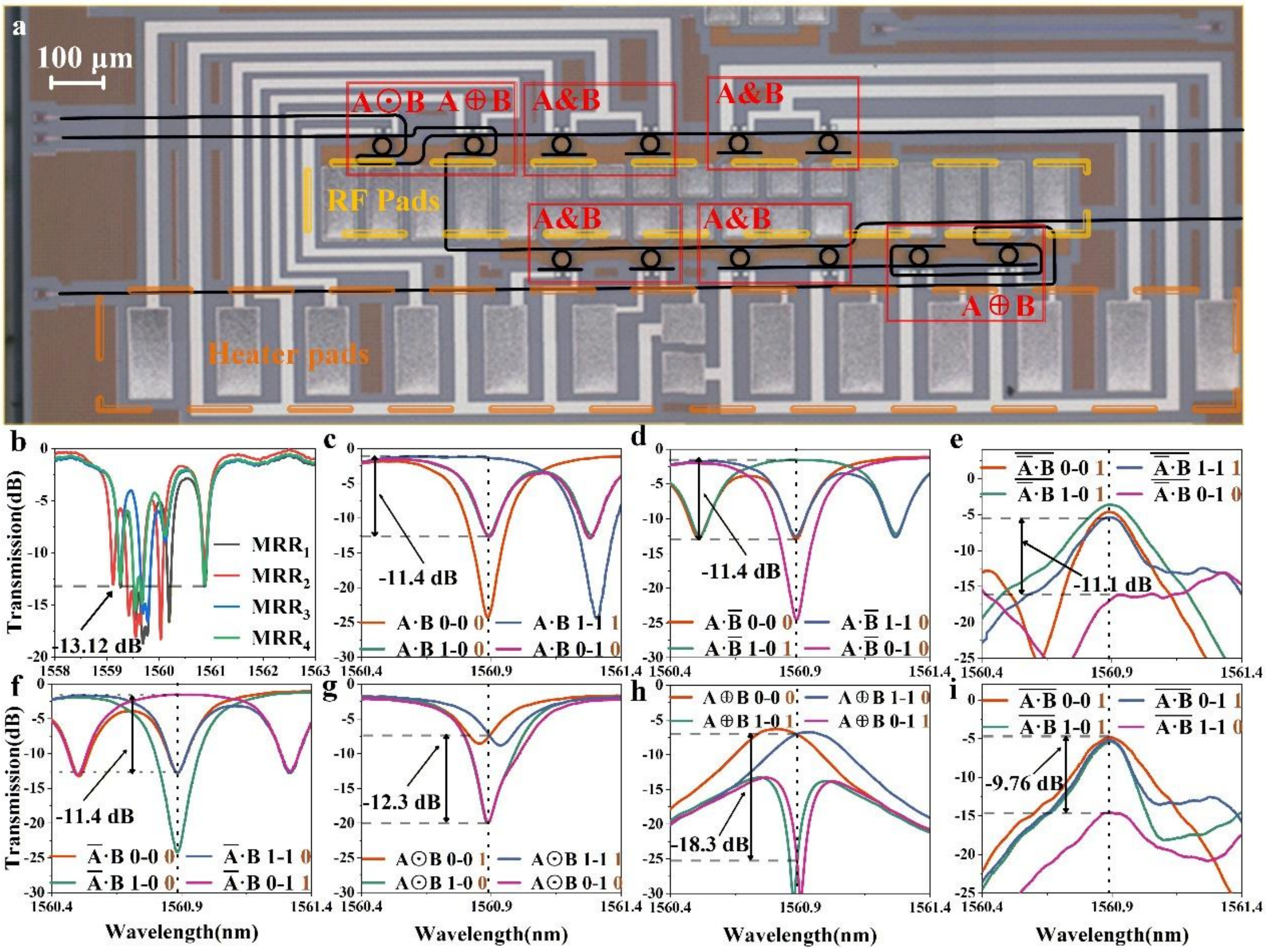

Figure R8. Micrograph with scale bar and light propagation path.

We believe that these revisions enhance the readability of the figure and better support the understanding of the proposed design. Furthermore, we revise the manuscript in the follow:

The proposed photonic computing device is fabricated on a silicon-on-insulator (SOI) platform comprising a 220-nm-thick top silicon layer and a 2-µm buried oxide layer, with a complete device micrograph shown in **Figure 2a**. And in Figure 2a, we have additionally added red boxes to indicate the units performing different BLUs, black lines to represent the waveguide routing, pads within orange boxes for applying thermal signals, and yellow boxes to denote the pads for high-speed RF signals.

To Reviewer #3's comments:

Recommendation: This work reports a reconfigurable optical arithmetic logic unit (ALU) on a silicon on insulator platform, in which microring modulators serve as the core functional elements. The device supports fundamental optical logic operations as well as fully reconfigurable 2-bit arithmetic functions, including addition, subtraction, and digital comparison. In addition to basic functional verification, the manuscript

further examines its potential utility in application-oriented scenarios such as high-speed data encryption based on optical XOR operations and image processing tasks including medical image watermarking and pathological tissue difference enhancement, which to some extent extends the discussion beyond proof-of-concept demonstrations. By addressing the current limitations of insufficient functional reconfigurability and low integration density in integrated photonic computing, this study presents a technical advance. The experimental results show that, at an operating speed of 20 Gb/s, the device achieves a computational density of 528 Gb/s/mm² and an energy efficiency of 12.36 fJ/bit, with performance metrics that compare favorably with existing electro optical computing schemes and that support the advantages of the MRM based architecture in terms of footprint and power consumption for photonic digital computing.

Overall, this research is well-conceived with a rigorous experimental design and solid, reproducible results. The proposed reconfigurable optical ALU enriches the technical repertoire of on-chip photonic computing and provides a versatile hardware scheme for next-generation high-performance computing systems that demand high speed, low power consumption and functional flexibility. This manuscript is deemed suitable for publication in Nature Communications following appropriate revisions to address the critical issues outlined below, which will further enhance the scientific rigor, content completeness and academic impact of the work.

Author Response:

We sincerely thank the reviewer for the comprehensive and constructive evaluation of our work. We greatly appreciate the positive recognition of the significance of our reconfigurable optical ALU, particularly in terms of functional flexibility, integration density, and its potential relevance to high-speed, low-power photonic computing systems.

We are also grateful for the reviewer's acknowledgment of the experimental rigor and the effort to extend the demonstrations toward application-oriented scenarios. At the same time, we fully agree that further clarification and refinement are necessary to enhance the scientific rigor, completeness, and overall impact of the manuscript.

In response, we have carefully revised the manuscript to address the critical issues raised. Specifically, we have strengthened the quantitative analysis of key performance metrics, clarified the architectural positioning and reconfigurability, and expanded the discussions on scalability, system-level implementation, and practical limitations. These revisions aim to provide a more transparent and rigorous presentation of the work and to better support its relevance to future high-performance photonic computing systems.

We believe that these improvements substantially enhance the quality and clarity of the manuscript, and we sincerely thank the reviewer for the insightful comments that have helped us strengthen this work.

Comment 3-1:

The work leverages wavelength-division multiplexing for parallel 2-bit processing, but no quantitative characterization of inter-wavelength crosstalk between the employed wavelengths (e.g., λ_1, λ_2) is provided. What is the measured crosstalk level between the wavelength channels, and how does this crosstalk impact the logic state integrity of parallel arithmetic operations? Please add experimental data on crosstalk and discuss its implications for higher-bit WDM-based processing.

Author Response:

We thank the reviewer for this important and technically relevant comment. We agree that quantitative characterization of inter-wavelength crosstalk is essential for evaluating the reliability of wavelength-division multiplexing (WDM)-based parallel processing.

In this comment, we have added additional experimental results to explicitly investigate the inter-wavelength crosstalk and its impact on logic operation integrity.

Specifically:

(1) Two-wavelength crosstalk characterization.

We performed experiments using two wavelength channels (λ_1 and λ_2) assigned to independent logic operations. By properly spacing the operating wavelengths to avoid spectral overlap of the microring resonances, in Figure R7, we observe that the

transmission responses of the two channels remain well isolated. The experimental results show that each wavelength channel can perform its corresponding logic function independently, with no observable degradation in logic state integrity. This indicates that the inter-wavelength crosstalk is sufficiently suppressed under the current design.

(2) Extension to three-wavelength operation.

To further evaluate scalability, we extended the experiment to three wavelength channels. The results demonstrate that all three channels can operate simultaneously and independently, each executing a different logic function, as shown in Figure R6. This confirms that the proposed architecture maintains low inter-wavelength interference even as the number of channels increases.

(3) Impact on logic correctness.

From the measured results, the logic outputs under multi-wavelength operation are consistent with those obtained in single-wavelength operation, indicating that inter-wavelength crosstalk does not introduce observable logic errors within the tested configuration. This suggests that the spectral isolation provided by the microring resonators is sufficient to preserve logic-level discrimination.

(4) Implications for scalability.

The inter-wavelength crosstalk is primarily determined by the resonance linewidth, channel spacing, and extinction ratio of the microring resonators. While the current results demonstrate reliable operation for up to three wavelength channels, scaling to a larger number of channels will require careful optimization of these parameters, such as increasing channel spacing, improving resonance quality factors, and employing more precise thermal tuning.

These results and discussions have been incorporated into the revised manuscript to provide a more complete and quantitative assessment of WDM performance. We believe this addition addresses the reviewer's concern and strengthens the validation of parallel multi-wavelength operation.

Comment 3-2:

The manuscript does not address how the input optical signal power impacts the logic operation performance (e.g., extinction ratio, signal-to-noise ratio (SNR) of the

output). What is the minimum/maximum input optical power range for reliable logic/ALU operation?? This is critical for practical system integration where input power may vary.

Author Response:

We thank the reviewer for this important and practically relevant comment. We agree that the tolerance of the proposed system to input optical power variations is critical for evaluating its robustness and applicability in realistic scenarios.

In this response, we have added experimental characterization of the system performance under different input optical power levels. Specifically, we performed high-speed measurements with input optical powers of 21 dBm to 13 dBm, and analyzed the corresponding eye diagrams.

The results show that, as the input optical power decreases, the eye opening gradually degrades and the signal-to-noise ratio (SNR) decreases, while the logic functionality remains correct within the tested range. This trend indicates that the system maintains functional robustness over a relatively wide input power window, but with reduced noise margin at lower powers.

The observed SNR degradation can be attributed to several factors:

(1) EDFA operating threshold.

As noted by the reviewer, when the input power before the erbium-doped fiber amplifier (EDFA) falls below its optimal operating range, the amplifier provides insufficient gain, leading to reduced output SNR.

(2) Accumulated insertion loss.

The propagation loss of waveguides, coupling loss, and device insertion loss reduce the effective optical power reaching the detector, making the signal more susceptible to noise.

(3) Detector noise and thermal noise.

At lower input power levels, the detected photocurrent decreases, while the relative impact of thermal noise and shot noise becomes more significant, degrading the eye quality.

(4) Finite extinction ratio of MRMs.

The limited extinction ratio of the microring modulators results in incomplete separation between logic states, which becomes more pronounced at lower optical power levels.

(5) Resonance detuning sensitivity.

Small wavelength drifts or thermal fluctuations can introduce additional signal fluctuations, particularly when the signal power is low and the system is more sensitive to perturbations.

(6) Minimum input power requirement.

Based on the experimental results, we observe that reliable logic/ALU operation can be maintained down to approximately 9 dBm in the current setup, below which the eye opening becomes significantly degraded. This value is influenced by the specific link configuration (including EDFA and coupling losses) and can be improved through system-level optimization.

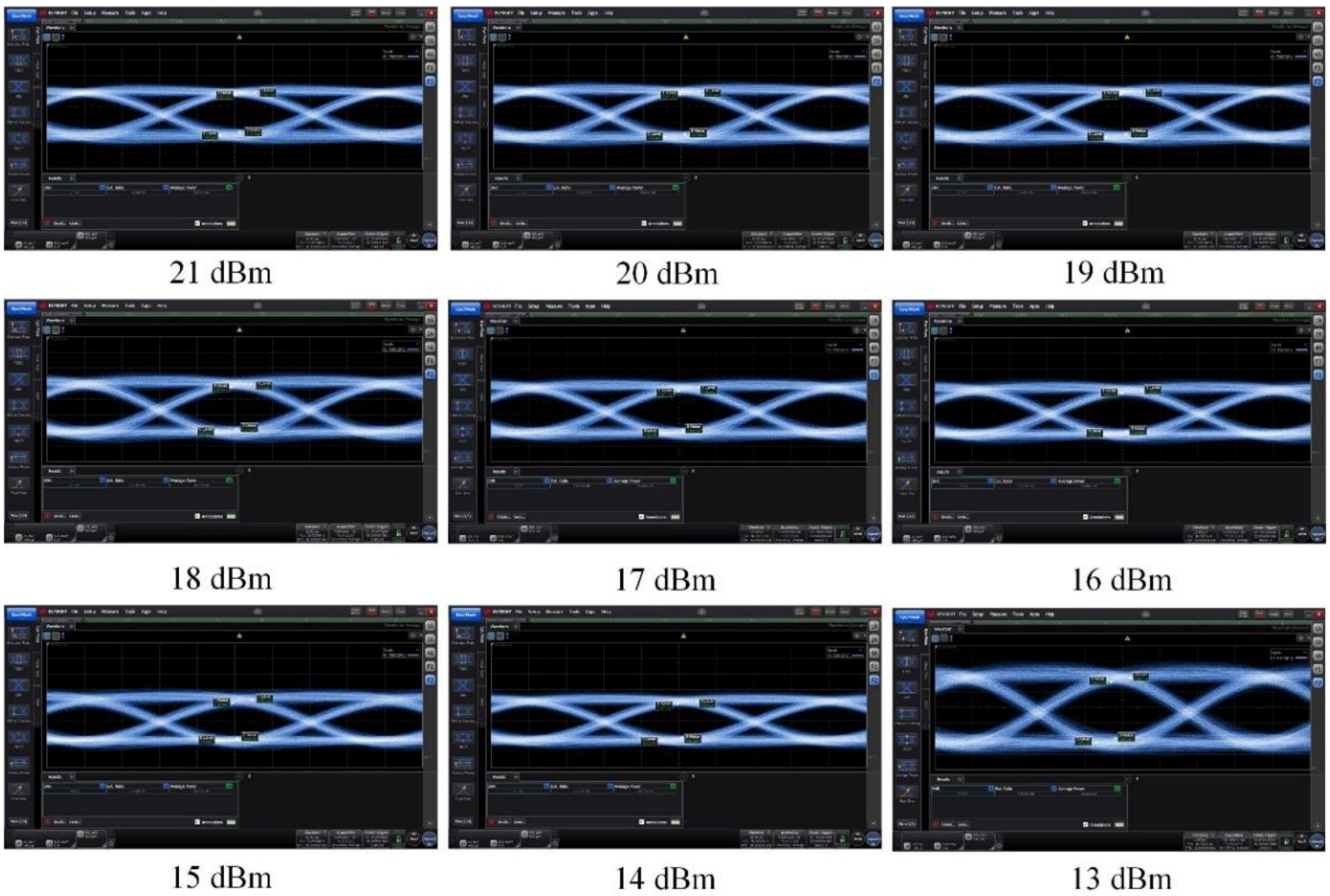

Figure R9. 20 Gbit/s eye diagrams at various input powers.

(7) Maximum input power consideration.

We agree that the upper bound of input optical power is also important. In silicon photonic waveguides, the maximum allowable optical power is typically limited by nonlinear absorption effects (e.g., two-photon absorption and free-carrier absorption)

and potential material damage.

Previous studies have shown that silicon waveguides can sustain optical powers on the order of tens to hundreds of milliwatts (>20 dBm) before significant nonlinear effects or damage occur, depending on waveguide geometry and operation conditions.[W1,W2]

We have added a discussion clarifying that the acceptable input power range is determined by a trade-off between maintaining sufficient SNR and avoiding nonlinear or thermal effects. For large-scale systems, this range can be further optimized through: low-loss photonic design, improved modulator extinction ratio, and optimized amplifier and detector configurations.

In summary, the added experimental results demonstrate that the proposed system maintains correct logic functionality over a range of input optical powers, while the SNR degrades at lower power due to both amplifier and device-level limitations. We believe this addition provides a more complete evaluation of system robustness and addresses the reviewer's concern.

References

W1 Singer S, et al. Nonlinear loss and damage threshold in silicon photonic waveguides: Modelling and experimental verification. In: CLEO: Science and Innovations). ***Optica Publishing Group*** (2022).

W2 Tsang H, Liu Y. Nonlinear optical properties of silicon waveguides. ***Semicond Sci Technol*** 23, 064007 (2008).

Comment 3-3:

The manuscript touts photonic computing's low latency advantage, but lacks a quantitative breakdown of end-to-end latency (including optical signal propagation, MRM modulation, electrical OR operation). Report total latency for a single 2-bit arithmetic operation, distinguish optical/electrical processing latency ratios, and compare with state-of-the-art electronic 2-bit ALUs.

Author Response:

We thank the reviewer for this important and quantitative suggestion. We agree that a clear breakdown of end-to-end latency is essential to substantiate the claimed

low-latency advantage of photonic computing.

In the revised manuscript, we have added a detailed latency analysis for a single 2-bit arithmetic operation, including both optical and electrical contributions.

(1) Optical-domain latency

The total optical latency consists of two main components:

MRM modulation response time: Based on our high-speed measurements at 20 Gbit/s, the rise and fall times of the microring modulators are both < 20 ps, which defines the dominant temporal response of the optical logic operation.

Optical signal propagation delay: The propagation delay is determined by the on-chip waveguide length and group index. For the current device, this delay is approximately ~3 ps.

Combining these contributions, the total on-chip optical latency is ~23 ps, which represents the intrinsic device-level processing time.

(2) Electrical-domain contributions

In addition to the optical processing, the overall system involves limited electrical-domain operations, including:

Electrical driving signal distribution: The delay is minimized through short interconnects and high-speed drivers, typically on the order of a few picoseconds in integrated configurations.

Backend logic (e.g., OR operation or signal decision): These operations are implemented using high-speed electronics and DSP. While their latency depends on the specific implementation, they can be pipelined and parallelized, and are not on the critical path of the optical computation.

We clarify in the revised manuscript that the reported ~23 ps latency corresponds to the core optical processing path, while additional electrical processing introduces a relatively small overhead in a co-designed system.

(3) Optical vs. electrical latency ratio

From the above analysis, the dominant latency contribution arises from the optical modulation process, while the propagation delay is negligible and electrical contributions can be effectively amortized or decoupled through system design. This

indicates that the architecture benefits from the inherently fast response of photonic devices.

(4) Comparison with electronic implementations

We have added a comparative discussion noting that, in electronic systems, the latency of multi-bit arithmetic operations is typically limited by: transistor switching speed, interconnect RC delay, and carry propagation in arithmetic units.

Even in advanced CMOS technologies, the latency of small-bit ALUs generally falls in the tens to hundreds of picoseconds range, depending on design and clocking schemes. In contrast, the proposed photonic ALU demonstrates a device-level latency of ~23 ps, highlighting its potential advantage in high-speed, low-latency computing scenarios.

In summary, we have quantified the end-to-end latency of the proposed system, showing that the intrinsic optical processing can be completed within ~23 ps, with minimal additional overhead from electrical components. These results support the low-latency advantage of the photonic approach and have been incorporated into the revised manuscript.

We thank the reviewer for this suggestion, which has significantly improved the quantitative rigor of our performance evaluation.

Comment 3-4:

Temperature variations in environments may induce resonance drift in thermally tunable devices within the device, particularly for components such MRRs, thereby affecting operational stability. The authors are encouraged to further discuss possible temperature stabilization strategies to mitigate such drift, or alternatively to explore device design approaches that can intrinsically reduce thermal sensitivity.

Author Response:

We thank the reviewer for this important and practically relevant comment. We agree that temperature-induced resonance drift is a critical factor affecting the stability of microring-based photonic systems and deserves a more comprehensive discussion.

In the revised manuscript, we have expanded the discussion on the origin of thermal sensitivity and potential stabilization strategies from both device-level and

system-level perspectives.

(1) Origin of resonance drift: thermal crosstalk and environmental fluctuations

In microring resonator, resonance wavelength shifts are primarily governed by the thermo-optic effect in silicon. In our current implementation, the dominant source of resonance drift arises from thermal crosstalk between adjacent MRMs, rather than ambient temperature fluctuations alone.

To mitigate this effect, we have adopted a spatial separation strategy in the layout design. Specifically, the spacing between adjacent resonators is increased to approximately 200 μm, which significantly reduces heat diffusion and minimizes unintended resonance shifts induced by neighboring devices. The experimental results confirm that this approach effectively suppresses inter-device thermal interference.

(2) On-chip thermal isolation engineering

Beyond spatial separation, we discuss additional device-level approaches to further suppress thermal crosstalk:

Thermal isolation trenches: Introducing etched isolation trenches (air gaps) around the MRMs can significantly reduce lateral heat conduction through the substrate. This method has been widely used in silicon photonics to improve thermal confinement and reduce cross-heating effects.

Optimized heater design: Engineering the geometry and placement of microheaters can localize heating more effectively, thereby reducing unintended thermal spreading to adjacent structures.

(3) System-level temperature stabilization

To address global temperature fluctuations, we outline the use of external thermal control mechanisms, including:

Thermoelectric cooling (TEC): A TEC module can be employed to stabilize the chip temperature at a constant operating point, thereby minimizing drift due to environmental variations.

Package-level thermal management: Advanced packaging with controlled heat dissipation pathways can further improve temperature uniformity across the chip.

(4) Active feedback control for resonance stabilization

We further discuss the implementation of closed-loop feedback control systems to dynamically compensate for residual thermal drift:

By monitoring the output optical power or resonance condition of each MRM in real time. The corresponding heater voltage can be adaptively adjusted to maintain the desired resonance alignment.

Such feedback mechanisms have been demonstrated to effectively stabilize microring-based systems against both thermal crosstalk and ambient fluctuations, enabling robust long-term operation.

(5) Design strategies for reduced thermal sensitivity

In addition to active stabilization, we note that intrinsic thermal sensitivity can be reduced through: Athermal design approaches (e.g., material or structural compensation), and wider resonance linewidth operation, which relaxes the sensitivity to wavelength drift at the expense of selectivity.

In summary, we have clarified that thermal crosstalk is the primary source of resonance drift in the current system and demonstrated that it can be effectively mitigated through layout optimization (e.g., 200 μm spacing). We further outline a combination of on-chip thermal isolation, external temperature control (TEC), and active feedback stabilization as practical solutions for improving system robustness. These discussions have been incorporated into the revised manuscript to provide a more complete assessment of operational stability.

We thank the reviewer for this valuable suggestion, which has significantly strengthened the practical relevance of our work.

Comment 3-5:

A few typos to be corrected:

Line 2, Page 13: “performance”.

Line 11, Page 15: “make as”.

Author Response:

We thank the reviewer for carefully identifying these typographical errors.

In the revised manuscript, we have corrected:

“performance” in Line 2, Page 13, and

“make as” in Line 11, Page 15.

We appreciate the reviewer’s attention to detail, which has helped improve the overall quality of the manuscript.

Comment 3-6:

Figure 2(a) seems to be lacking a scale bar.

Author Response:

We thank the reviewer for this helpful comment.

In the revised manuscript, we have added a scale bar to Figure 2(a) to clearly indicate the physical dimensions of the device. This addition improves the clarity and interpretability of the figure.

We appreciate the reviewer’s suggestion, which has helped enhance the presentation quality.

Comment 3-7:

In recent two years, there are some significant advances in optical arithmetic logic unit and digital optical computing, the authors should credit these related papers.

Author Response:

We thank the reviewer for this valuable suggestion. We fully agree that recent advances in optical arithmetic logic units and digital optical computing should be properly acknowledged to better position this work within the current research landscape.

In the revised manuscript, we have incorporated and discussed several recent representative studies on optical logic operations and photonic digital computing. These include works on integrated photonic logic gates, reconfigurable optical computing architectures, and high-speed photonic information processing systems. The added references highlight recent progress in areas such as wavelength-division multiplexed logic, photonic tensor computing, and optical neural/logic hybrid systems.

We have updated the Introduction and Discussion sections to better contextualize our work relative to recent developments in optical logic and digital photonic

computing, clarify the distinctions between prior demonstrations (e.g., fixed-function logic gates or task-specific processors) and our reconfigurable ALU architecture, and explicitly acknowledge the contributions of these recent studies.

We believe that these additions improve the completeness of the literature review and strengthen the positioning of our work within the rapidly evolving field of photonic computing.

We thank the reviewer for this important comment, which has helped enhance the scholarly depth and balance of the manuscript. Here shows the revised manuscript as follow:

Although recent works have demonstrated large-scale optical computing systems capable of implementing logic-related operations, significantly advancing the development of photonic logic computing, these approaches generally rely on large and complex network architectures, which differ from compact and reconfigurable ALU-oriented designs

In answer 1-1: What is the resistance of the TiN heater? Also, please clarify what is meant by "exhibits a nonlinear dependence on heating power." This shift is expected to be linear with power. If not, please explain the underlying reason for the observed nonlinearity.

Response:

We thank the editor for this helpful question and apologize for the ambiguity in our previous description. The TiN microheater used in this work has a resistance of approximately 200 Ω. In the revised Supplementary Information, we have explicitly added this value together with the corresponding electrical tuning parameters. Regarding the statement that the resonance shift "exhibits a nonlinear dependence on heating power," we would like to clarify that the observed nonlinearity refers to the dependence on the applied heating voltage rather than on the dissipated electrical power. Under the operating conditions, the resistance of the TiN heater remains approximately constant, and the generated thermal power follows Joule's law, $P = U^2 / R$. Meanwhile, the thermo-optic coefficient of silicon is nearly linear within the relevant temperature range, leading to an approximately linear relationship between resonance wavelength shift and temperature (or equivalently, heating power). Consequently, the resonance shift exhibits a nonlinear dependence on the applied voltage. To avoid confusion, we have revised the manuscript and Supplementary Information to explicitly distinguish the dependence on heating voltage and heating power, and have clarified the physical origin of the observed tuning behavior. The revised Supplementary Note 3 are showing below:

(2) Thermal tuning via TiN microheater.

To compensate for fabrication-induced wavelength detuning and environmental fluctuations, a TiN microheater is integrated for thermo-optic tuning. The heating voltage (or equivalently electrical power) is varied from 0V to 4V, and the resulting resonance shift is extracted from the measured spectra.

The TiN microheater exhibits a resistance of approximately 200 Ω. The resonance wavelength shift is approximately linear with the dissipated electrical power, consistent

with the nearly linear thermo-optic response of silicon. When expressed as a function of the applied heating voltage, the resonance shift shows a nonlinear dependence due to the Joule heating relation $P = U^2 / R$.

In answer 1-2: Looking at Figure 2, the actual footprint seems larger than the value provided in the text. Please verify or clarify the discrepancy.

Response:

We thank the editor for this careful observation and appreciate the opportunity to clarify the footprint definition used in this work. The footprint reported in the manuscript refers only to the active computational region, namely the microring modulators and their immediately adjacent functional waveguides that directly participate in optical logic operations. For clarity, this active area has now been explicitly marked in the revised Figure 2. The larger overall layout visible in Figure 2 mainly originates from test-access structures required for experimental characterization rather than from the computational device itself. Specifically, two factors contribute to the additional area: (1) To enable simultaneous optical and electrical probing, sufficient spacing is reserved between the grating couplers and the probe pads. The intermediate waveguides only serve as optical transmission paths and do not participate in the computation process. (2) The metal routing and pad arrangement are constrained by the probe configurations available in our laboratory. In particular, the thermo-optic tuning probes require a pitch of 200 μm, whereas the electro-optic tuning probes use a pitch of 100 μm, resulting in a larger overall wiring footprint for measurement accessibility. These test-access structures represent experimental overhead rather than intrinsic device requirements and can be substantially reduced in a practical integrated implementation. Therefore, the area used for calculating the computational density metrics corresponds exclusively to the active logic units, whose actual footprint is significantly smaller than the total chip area shown in Figure 2. To avoid ambiguity, we have revised the manuscript to explicitly distinguish the active computational footprint from the full experimental layout and have highlighted the corresponding active region in Figure 2. The revised part in Discussion is showing below:

The proposed architecture employs MRMs to implement digital logic computing, offering significant advantages in integration density, power efficiency, and modulation performance. To balance integration scale with modulation capability, the microring resonators are designed with a radius of 20 μm, substantially more compact than conventional MZI-based modulators. **The reported device dimensions of 1.42 × 0.32 mm² correspond to the active computational footprint, which includes only the MRMs and their adjacent functional waveguides directly involved in logic operations. The larger layout shown in Figure 2 additionally contains passive routing waveguides, optical access ports, and electrical probe-pad structures required for experimental characterization, and these components are not included in the computational density evaluation. The current experimental implementation is primarily constrained by the 100 μm pitch requirement for high-frequency electrical probes and the 200 μm pitch requirement for thermo-optic tuning probes. With optimized optoelectronic co-packaging and electrical routing, the overall footprint could be further reduced to 0.66 × 0.25 mm².**

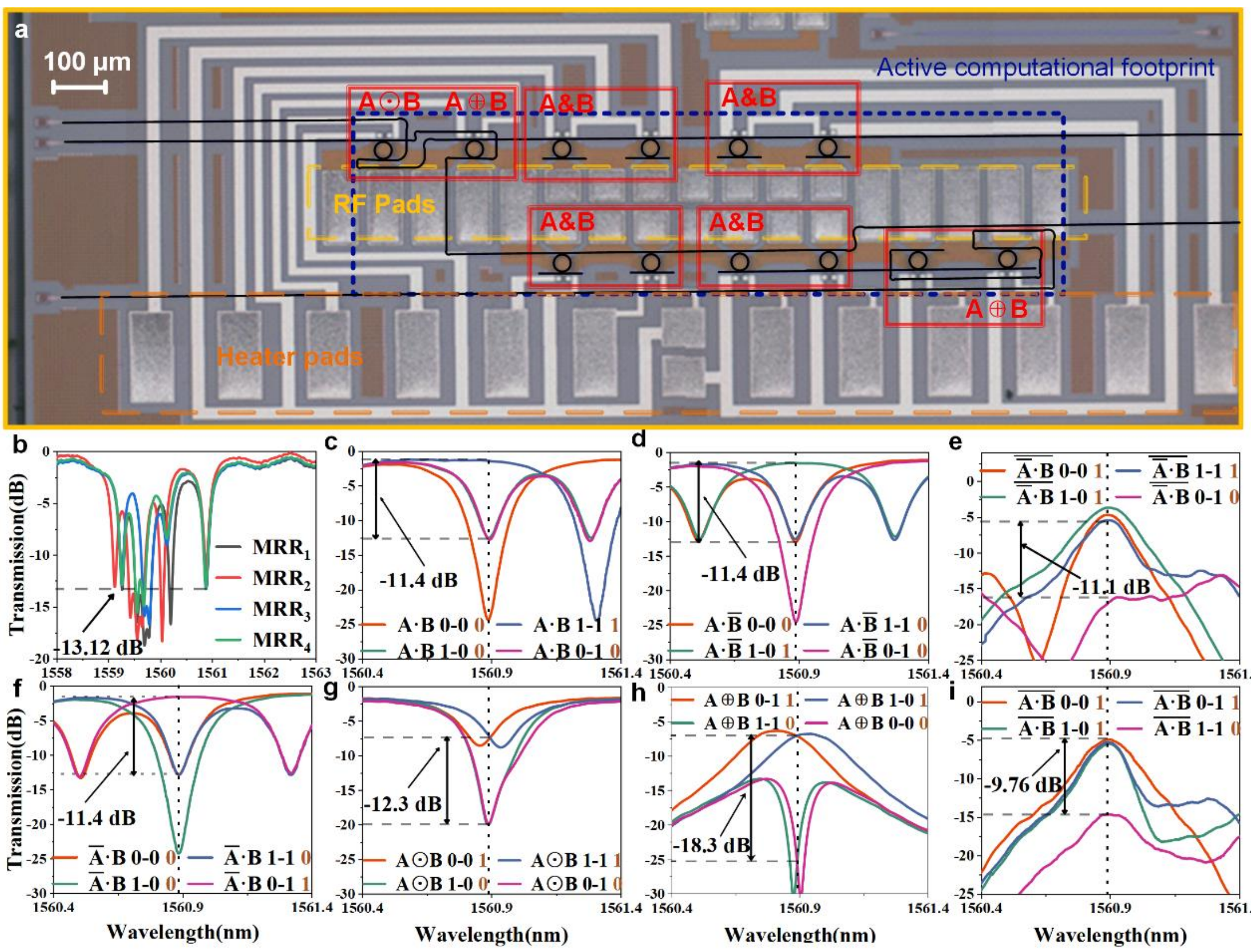

Figure 2. Architectural micrographs and basic logic operation characterization. **a** The microscope image of the proposed device with different region. **b** The different microrings resonator at same wavelength. **c-i** static spectral response of different fundamental optical operations **c** $A \cdot B$ **d** $A \cdot \bar{B}$ **e** $\overline{\bar{A} \cdot B}$ **f** $\bar{A} \cdot B$ **g** $A \odot B$ **h** $A \oplus B$ **i** $\overline{A \cdot B}$.

Reviewer #2 (Remarks to the Author):

I would like to thank the authors for their detailed and comprehensive responses to my previous comments. The authors have fully addressed all of my concerns. While the proposed time-domain approach to address the scalability concern may not be the ideal solution, it remains an acceptable approach for this stage of development. The authors also have clearly illustrated the inherent trade-off between throughput and operational performance in the revised manuscript. Therefore, I recommend this paper for publication after a thorough proofreading to eliminate minor grammatical issues. For instance, the phrasing in the caption of Table 1 ('1-bit ALUs' the truth table and expression') needs to be rectified.

Response:

We sincerely thank the reviewer for the positive evaluation of our revised manuscript and for recognizing our efforts in addressing the previous comments. We appreciate the reviewer's acknowledgment that the proposed time-domain approach provides an acceptable solution to the scalability issue at the current stage, and that the revised manuscript clearly presents the trade-off between throughput and operational performance.

Following the reviewer's suggestion, we have carefully proofread the entire manuscript to correct minor grammatical issues. In particular, the caption of Table 1 has been revised from "1-bit ALUs' the truth table and expression" to "Truth table and logic expressions for the 1-bit optical ALU operations" to improve grammatical correctness and clarity.

We thank the reviewer again for the constructive comments and recommendation for publication.

Reviewer #3 (Remarks to the Author):

Since the authors have addressed the concerns that the reviewers pointed, I can

accept manuscript as it is.

Response:

We sincerely thank the reviewer for the positive evaluation of our revised manuscript and for acknowledging that the concerns raised during the review process have been adequately addressed. We greatly appreciate the reviewer's support and recommendation for publication.
