# Supplementary material for: Reconfigurable optical arithmetic logic unit and its applications
Source: Nat Commun. 2026 Jul 18;17:8793. doi: 10.1038/s41467-026-75750-x (PMC13494030; doi:10.1038/s41467-026-75750-x)
Supplement: Supplementary file 2 [file 41467_2026_75750_MOESM1_ESM.docx]

Supplementary information for

**Reconfigurable Optical Arithmetic Logic Unit and Its Applications**

X. Zhou^†^, M, Yuan^†^, H. Xiao, Y. Jiang, P. Zhang, Y. Tian*

School of Physical Science and Technology, Lanzhou University, Lanzhou 730000, Gansu, China

† These authors contributed equally

* Corresponding author: tianyh@lzu.edu.cn

**This supplementary information file includes:**

Supplementary Note 1 - Advantages of MRM-based implementation for BLU and ALU operations.

Supplementary Note 2 - Basic principles of tuning state for MRM.

Supplementary Note 3 - Electrical and Thermal Tuning Characteristics of the MRM.

Supplementary Note 4 - Verifying the feasibility of OR operations in electric domains.

Supplementary Note 5 - System-level DC characterization of multi-MRM circuits.

Supplementary Note 6 - DSP description.

Supplementary Note 7 - Power consumption analysis and computational density of the device.

Supplementary Note 8 - The details of different amplification for image application.

Supplementary Note 9 - Scalability toward multi-bit photonic ALUs.

Supplementary Note 10 - Advantages of photonic ALUs for high-throughput computation

Supplementary Note 1 - Advantages of MRM-based implementation for BLU and ALU operations

The proposed architecture leverages microring modulators (MRMs) as the fundamental building blocks for both basic logic units (BLUs) and higher-level arithmetic logic unit (ALU) functions. While resonance-based switching is a well-established mechanism, its use in the present work provides specific advantages at both the BLU and system levels, which are clarified below.

At the level of BLU implementation, MRMs enable a compact and efficient realization of logic operations through resonance-controlled transmission states. The transition between pass and block states is achieved via small refractive index perturbations, allowing logic operations to be directly mapped onto optical routing behavior. Compared to interferometric implementations, this resonance-based mechanism does not require precise phase matching over extended optical paths, which reduces sensitivity to phase noise and fabrication-induced imbalance. In addition, the localized nature of the resonant cavity allows BLUs to be implemented with a significantly reduced footprint, facilitating dense integration.

Another important characteristic at the BLU level is the high modulation efficiency enabled by resonance enhancement. Small electrical perturbations can induce large changes in optical transmission, which lowers the required driving voltage and improves energy efficiency. This property is particularly beneficial when multiple BLUs are cascaded, as it helps maintain sufficient signal contrast without excessive electrical overhead.

At the ALU level, the use of MRMs provides structural advantages in terms of modularity and reconfigurability. Since each BLU operates based on the same underlying resonance tuning mechanism, different logic and arithmetic functions can be realized through consistent control of electrical inputs, without modifying the physical interconnect topology. This enables a unified mapping between optical device states and digital logic operations, which is essential for constructing a reconfigurable ALU architecture.

Furthermore, the compact footprint of MRMs allows multiple BLUs to be integrated within a limited area, supporting the implementation of composite operations such as addition, subtraction, and comparison. This high integration density is critical for scaling toward more complex computational units, where spatial resource constraints become a dominant factor. As summarized in Supplementary Table 1, compared with representative prior works, the proposed architecture supports a larger number of BLUs and multiple ALU-level functions within a unified framework, while maintaining competitive operation speed. This comparison highlights that the advantage of the present work lies not in individual device performance, but in the level of architectural integration and functional programmability.

Supplementary Table 1 Comparison of representative optical logic implementations

| Work | OU. | BLU | ALU | R.f. | Fre.  [Gbit/s] |
| --- | --- | --- | --- | --- | --- |
| [S1] | DC | 6 | 3 | No | / |
| [S2] | MRR | / | 3 | Yes | / |
| [S3] | MRM | >3 | / | Yes | / |
| [S4] | MRM+PD | >6 | / | Yes | 9.4 |
| [S5] | MRM | 3 | 1 | Yes | / |
| [S6] | MZI+MRR | >7 | 1 | No | / |
| [S7] | MZI | / | 1 | Yes | 20 |
| [S8] | PLA | / | 3 | Yes | 10 |
| [S9] | MZI | 2 | 1 | No | 20 |
| [S10] | MZI | >5 | / | Yes | 25 |
| This work | MRM | >7 | 3 | Yes | 20 |

Supplementary Note 2 - Basic principles of tuning state for MRM.

In the proposed device, MRMs serve as fundamental components for executing various basic logic operations through the application of different electrical signals. As detailed in the manuscript, each MRM operates in two distinct states—pass/block and block/pass—enabling flexible signal routing and logic reconfiguration. The operational principles underlying these states are elaborated as follows. The add-drop configuration of the microring resonators produces complementary responses at different output ports. At the through port, the resonant characteristic appears as a transmission dip, exhibiting a spectral profile that initially decreases then increases with wavelength. As illustrated in Supplementary Figure 1a, in pass/block mode, the application of a high-level voltage induces a red-shift in the resonant wavelength, leading to increased optical transmission intensity at the operating wavelength. Conversely, in block/pass mode (Supplementary Figure 1b), the same voltage condition results in decreased transmission intensity.

**Supplementary Figure 1.** Different state at different port of MRM. **a** Pass/block at through port. **b** Block/pass at through port. **c** Pass/block at drop port. **d** Block/pass at drop port.

At the drop port, the resonant behaviour is inverted, manifesting as a transmission peak rather than a dip. Consequently, the voltage-dependent intensity variations at the drop port are opposite to those observed at the through port, as demonstrated in Supplementary Figure 1c-1d. This complementary response between the output ports forms the physical foundation for implementing reconfigurable logic functions, whereby assigning different operating wavelengths to individual microring resonators enables diverse logic outputs, while tuning facilitates switching between the two operational states of a single resonator.

Supplementary Note 3 - Electrical and Thermal Tuning Characteristics of the MRM

To quantitatively characterize the switching behavior of the MRM used for logic operation, we systematically investigate its electrical and thermal tuning responses, including carrier-depletion-induced modulation, thermo-optic wavelength alignment, and dynamic switching performance.

(1) Electrical tuning via PN junction carrier depletion.

The MRM operates under reverse-bias conditions to modulate the effective refractive index through carrier depletion. The applied DC bias voltage is swept from *V_min_* to *𝑉_max_*, covering the full transition between the pass–block and block–pass states. The transmission spectra are recorded for each bias point, from which the resonance shift and extinction ratio are extracted.

The measured tuning curve (Supplementary Figure 2a) shows a monotonic dependence of resonance shift (and corresponding transmission) on the applied bias voltage, confirming stable and continuous electrical control within the operating window.

(2) Thermal tuning via TiN microheater.

To compensate for fabrication-induced wavelength detuning and environmental fluctuations, a TiN microheater is integrated for thermo-optic tuning. The heating voltage (or equivalently electrical power) is varied from 0V to 4V, and the resulting resonance shift is extracted from the measured spectra.

The TiN microheater exhibits a resistance of approximately 200 Ω. The resonance wavelength shift is approximately linear with the dissipated electrical power, consistent with the nearly linear thermo-optic response of silicon. When expressed as a function of the applied heating voltage, the resonance shift shows a nonlinear (quadratic) dependence due to the Joule heating relation.

(3) Dynamic response of state switching.

The temporal response of the MRM is characterized by applying a square-wave electrical signal and recording the output optical intensity using a high-speed photodetector. The rise time (𝑡_𝑟_) and fall time (𝑡_𝑓_) are extracted based on the 10–90% transition criterion.

The measured response (Supplementary Figure 2c) confirms that the device supports picosecond-scale switching speeds, which are sufficient for the targeted logic operation bandwidth.

**Supplementary Figure 2.** Characterization of the MRM. (a) Transmission spectra of the MRM under varying bias voltages. (b) Transmission spectra of the MRM under thermal tuning. (c) Rising and falling edges of the MRM at 20 Gbit/s

Supplementary Note 4 - Verifying the feasibility of OR operations in electric domains

Owing to the limited availability of a four-channel arbitrary waveform generator (AWG) during high-speed testing, the experimental characterization of basic logic operations was performed using a two-channel AWG configuration. To implement the required OR operations between different basic logic outputs, these logical combinations are subsequently processed in the electrical domain. To validate this methodological approach, we conducted comparative experiments using a four-channel AWG operating at lower speeds to physically generate the OR operations between various basic logic functions. The resulting waveforms were systematically compared with those obtained through electrical-domain OR processing of two-channel AWG measurements. This comparative analysis confirms the functional equivalence between direct multi-channel AWG generation and electrical-domain signal processing, thereby verifying the reliability of our experimental methodology for implementing combinational logic operations.

**Supplementary Figure 3** Experimental results of optical-domain and electrical-domain OR logic operations. **a** The results of${(A}_{2}⨀B_{2})$, ${(A}_{1}\cdot\bar{B_{1}})$, ${(A}_{2}⨀B_{2}){(A}_{1}\cdot\bar{B_{1}})$ which execute OR operation in optical domain, and ${(A}_{2}⨀B_{2}){(A}_{1}\cdot\bar{B_{1}})$ which execute OR operation in electrical domain, respectively. **b** The results of ${(A}_{2}\bigoplus B_{2})$, $(\bar{\bar{A_{1}}\cdot B_{1}})$, ${(A}_{2}\bigoplus B_{2})(\bar{\bar{A_{1}}\cdot B_{1}})$ which execute OR operation in optical domain, and ${(A}_{2}\bigoplus B_{2})(\bar{\bar{A_{1}}\cdot B_{1}})$ which execute OR operation in electrical domain, respectively.

To validate the methodology, we examined the cases of ${(A}_{2}⨀B_{2}){(A}_{1}\cdot\bar{B_{1}})$ and ${(A}_{2}\bigoplus B_{2})(\bar{\bar{A_{1}}\cdot B_{1}})$ at 100 bit/s as representative examples. As shown in Figure S3a, the measured waveforms demonstrate: the green trace represents${(A}_{2}⨀B_{2})$, the blue trace shows ${(A}_{1}\cdot\bar{B_{1}})$, the red trace displays the result obtained by applying four different pseudorandom sequences to the thermal tuners to achieve OR operation in the optical domain, while the black trace corresponds to the electrical-domain OR operation performed on the two basic logic outputs. The close agreement between the black and red waveforms demonstrates that executing the OR operation in the electrical domain preserves signal integrity and produces equivalent results to the optical implementation. This conclusion is further supported by the additional comparative data presented in Figure S3b, which shows consistent behavior across different operational conditions and parameter variations. The comprehensive comparison confirms the viability of electrical-domain signal processing as a reliable alternative for implementing combinational logic operations in photonic computing systems, particularly when facing equipment limitations in experimental setups.

Supplementary Note 5 - System-level DC characterization of multi-MRM circuits

To further validate the system-level integration capability of the proposed architecture beyond the two-MRM high-speed demonstration, we perform comprehensive DC characterization involving multiple MRMs and wavelength channels. These measurements are designed to verify the functionality of cascaded BLUs under multi-channel operation and to assess the feasibility of scaling toward larger circuits.

First, we demonstrate parallel logic operations using two independent wavelength channels, as shown in Supplementary Figure 4. In this configuration, two distinct wavelengths are simultaneously injected into the circuit, each performing a different logic operation. The experimental results confirm that both operations are correctly executed without mutual interference, indicating that the BLUs can operate independently across different wavelength channels and supporting the feasibility of wavelength-division multiplexing for parallel computation.

**Supplementary Figure 4.** Two wavelength channels with independent logic operations

To validate the fundamental functionality of the BLU at the single-channel level, we perform logic operations within a single wavelength channel. As presented in Supplementary Figure 5, AND operations are implemented using the BLU, and the measured outputs match the expected logic results. This confirms the correctness and robustness of the basic logic unit under single-wavelength operation.

**Supplementary Figure 5.** Spectra of two BLUs modulated on a single wavelength.

We further extend the demonstration to three wavelength channels, where three distinct logic operations are executed simultaneously, as shown in Supplementary Figure 6. The results show that all operations are correctly performed, with no observable functional degradation or interference between channels. This experiment verifies that multiple BLUs can operate concurrently in a multi-wavelength configuration, providing further evidence of the scalability of the architecture.

**Supplementary Figure 6.** Spectra of three distinct BLUs at three wavelengths.

In addition, we investigate a more complex multi-channel scenario involving independent wavelength pairs. As illustrated in Supplementary Figure 7, two separate wavelength pairs are used to perform two different logic operations independently within the same circuit. The experimental results confirm that these operations can be executed simultaneously without cross-channel interference, demonstrating the flexibility and configurability of the system in handling multiple logic functions in parallel.

**Supplementary Figure 7.** Spectra of two distinct BLUs at other two wavelengths

Collectively, these results show that, although high-speed measurements are conducted on a reduced two-MRM configuration, the DC characterization of multi-wavelength and multi-MRM operation provides strong evidence for correct functionality at the system level. The demonstrated capability of parallel, independent logic processing across multiple wavelength channels supports the scalability of the proposed architecture toward larger photonic computing systems.

Supplementary Note 6 - DSP description.

The optical waveforms acquired from the digital communications analyzer (DCA) are continuous-time analog signals, which cannot be directly interpreted as discrete binary logic levels due to amplitude fluctuations, timing jitter, and waveform distortion, especially under high-speed operation at 20 Gbit/s. To enable reliable data recovery and quantitative performance evaluation, a dedicated digital signal processing (DSP) framework is developed to convert the measured analog traces into standardized digital sequences.

As illustrated in Supplementary Figure 8a, the DSP workflow consists of three main stages: data frame identification and timing alignment, adaptive threshold determination, and logic-level decision for data reconstruction. The corresponding decision criteria are summarized in Supplementary Figure 8b, while the effectiveness of the DSP processing is validated through NRZ comparison before and after processing in Supplementary Figure 8c.

(1) Data frame identification and timing alignment

Due to the lack of strict synchronization between the arbitrary waveform generator (AWG) and the DCA, the captured waveform typically contains an arbitrary temporal offset relative to the transmitted data sequence. This misalignment, if uncorrected, leads to incorrect bit segmentation and degraded logic interpretation.

To address this issue, a cross-correlation-based synchronization method is employed. The measured waveform is correlated with a predefined reference sequence, and the temporal position corresponding to the maximum correlation coefficient is identified as the starting point of the valid data frame. This procedure effectively aligns the measured signal with the ideal bit sequence.

Once the starting position is determined, the waveform is segmented into discrete bit intervals according to the known data rate, with a bit duration of. This ensures that each bit is sampled within the correct temporal window, minimizing inter-symbol interference induced by timing offsets. The robustness of this synchronization step is critical for subsequent threshold-based decision processes.

(2) Adaptive threshold determination based on amplitude and duty-cycle statistics

At high data rates, the received optical signal exhibits non-ideal characteristics, including amplitude variation, baseline drift, and waveform asymmetry. Under such conditions, a fixed decision threshold is insufficient for reliable logic discrimination. Therefore, an adaptive thresholding strategy is adopted.

As shown in Figure S8b, the decision framework incorporates both amplitude-based and duty-cycle-based criteria:

Amplitude threshold:

The statistical distribution of the signal intensity within a data frame is analyzed to extract representative high-level and low-level values. Based on this distribution, an optimal decision threshold is defined to maximize the separation between logic “1” and logic “0” levels.

Duty-cycle criterion:

For each bit interval, the proportion of time during which the signal remains above the threshold is calculated. This duty-cycle metric provides an additional constraint to mitigate errors caused by waveform distortion, incomplete transitions, or timing jitter.

By jointly considering these two criteria, the algorithm achieves robust logic discrimination even when the waveform deviates from an ideal rectangular shape. This hybrid strategy is particularly important for maintaining decision reliability under 20 Gbit/s operation, where signal degradation becomes more pronounced.

(3) Logic-level decision and data reconstruction

Following synchronization and threshold determination, binary logic values are assigned to each bit interval. Instead of relying solely on instantaneous sampling or edge detection, the decision process is based on a statistical evaluation of the signal within each bit period.

Specifically, a bit is classified as logic “1” if (i) the average signal level exceeds the adaptive threshold and (ii) the duty-cycle criterion satisfies a predefined condition (i.e., the signal remains above threshold for a dominant fraction of the bit duration). Otherwise, the bit is assigned as logic “0”.

This dual-criterion decision mechanism improves robustness against noise, timing jitter, and partial waveform transitions, ensuring stable data reconstruction across varying signal conditions. The reconstructed binary sequence is subsequently used to recover the logic outputs of the implemented ALU operations.

**Supplementary Figure 8.** DSP framework for data recovery and its validation at 20 Gbit/s. **a** Three-stage DSP workflow: frame identification, adaptive threshold, and logic decision. **b** Joint decision criteria: amplitude statistics and duty cycle. **c** NRZ waveforms before and after DSP processing.

(4) Validation through long-sequence NRZ data and error analysis

To quantitatively evaluate the reliability of the proposed DSP framework under high-speed operation, we perform data transmission experiments using a non-return-to-zero (NRZ) signal with a sequence length of 10,000 bits at 20 Gbit/s. The experimentally acquired waveform is processed using the DSP procedure described above, and the reconstructed binary sequence is directly compared with the transmitted reference data.

The measured error rate is 4×10^−4^, corresponding to 4 errors per 10,000 bits. This result provides a direct assessment of system-level performance beyond qualitative visualization metrics such as eye diagrams.

To further understand the origin of the observed errors, we analyze the temporal waveform associated with erroneous bit positions. It is found that the dominant error mechanism arises in specific bit patterns characterized by long sequences of consecutive logic “1” followed by an isolated logic “0”. In such cases, the MRM exhibits a limited transient response when switching from a sustained high-transmission state to a low-transmission state, leading to incomplete extinction within a single period.

This behavior can be attributed to the combined effects of carrier dynamics and cavity photon lifetime, which introduce a finite response time and prevent ideal instantaneous switching. As a result, the optical intensity within the interval does not fully cross the decision threshold, leading to misclassification by the DSP algorithm.

Importantly, this error mechanism is rooted in the physical response limitation of the device rather than the DSP processing itself. The DSP framework faithfully reconstructs the waveform based on the defined decision criteria and does not artificially suppress or introduce errors.

For clarity, Figure S8c presents a comparison of the waveform before and after DSP processing. While the raw waveform exhibits amplitude fluctuation and timing uncertainty, the processed signal demonstrates clear logic-level discretization. The residual errors observed in the reconstructed sequence are consistent with the identified device-level limitations.

These results indicate that the proposed DSP framework enables reliable data recovery at 20 Gbit/s, while the remaining errors highlight the intrinsic speed limitation of the current device configuration under specific input patterns.

Supplementary Note 7 - Power consumption analysis and computational density of the device.

This section presents a comprehensive analysis of power consumption in the proposed photonic computing architecture. Through implementation of state-of-the-art fabrication techniques, the MRM achieves an exceptional power efficiency of 1.03 fJ/bit, with 0.79 fJ/bit allocated to dynamic modulation and 0.24 fJ/bit dedicated to thermal compensation^S11^. The architecture employs varying numbers of MRMs depending on the complexity of the implemented reconfigurable functions, with detailed power consumption specifications systematically documented in Supplementary Table 2 and corresponding schematic representations provided in Figure 1 of the main text.

Supplementary Table 2. Power consumption and other parameter of the device

| Reconfigurable function | Number of MRMs | Frequency | Power consumption (per bit) | Power consumption  (per second) | Note |
| --- | --- | --- | --- | --- | --- |
| Basic logical operation | 2 | 20 GHz | 2.06 fJ | 0.0412 mW | / |
|  |  | 80 GHz | 0.52 fJ | 0.0103 mW | Potential |
| Adder | 12 | 20 GHz | 12.36 fJ | 0.247 mW | / |
|  |  | 80 GHz | 3.09 fJ | 0.062 mW | Potential |
| Subtractor | 12 | 20 GHz | 12.36 fJ | 0.247 mW | / |
|  |  | 80 GHz | 3.09 fJ | 0.062 mW | Potential |
| Comparator | 6 | 20 GHz | 6.18 fJ | 0.123 mW | / |
|  |  | 80 GHz | 1.55fJ | 0.031 mW | Potential |

For the computational density, the architecture cascade 12 MRMs to achieve the 2-bit ALU, both of the MRM’s modulation speed are 20 Gbit/s. As mentioned in the manuscript, the footprint of the device is 1.42 ×0.32 mm^2^, so the computational density can be calculated as (20 Gbit/s× 12)/ (1.42 ×0.32 mm^2^) ≈ 528 Gbit/s/mm^2^.

The power consumption mentioned above takes into account only the on-chip MRM component; in actual experimental setups, the power consumption of devices such as lasers, EDFAs, AWGs, and DCAs must also be considered. Recent reports indicate that integrating all these components onto a single chip can significantly reduce the power consumption of the optical chip.

Supplementary Note 8 - The details of different amplification for image application.

While other demonstrated applications achieve 100% calculation accuracy with straightforward functional implementation, the differential amplification for image processing requires special consideration of its reconstruction principles. As a 2-bit subtractor, the output encompasses seven discrete decimal values ranging from -3 to +3. To transform these computational results into visual representations, multiple grayscale mapping schemes have been developed. For 7-bit grayscale reconstruction, we establish a direct linear mapping between the seven subtractor outputs (-3 to +3) and corresponding intensity levels. The 4-bit grayscale scheme employs the absolute values of the subtraction results, creating a one-to-one correspondence with reduced grayscale resolution. In the 3-bit implementation, grayscale assignment follows a ternary classification based on the result's sign: positive, zero, or negative values. The most compact 2-bit representation utilizes a binary classification that distinguishes between zero and non-zero outcomes, effectively replicating the functionality of an equality comparator. These graduated reconstruction techniques enable flexible trade-offs between image resolution and processing complexity, demonstrating the architecture's adaptability across various application requirements while maintaining mathematical consistency between computational outputs and their visual representations.

**Supplementary Figure 9** 7-bit grayscale images from electrical and optical computation and their residual difference **a** 7-bit grayscale image of electric domain computation. **b** 7-bit grayscale image of optical domain computation. **c** The different results of the above images.

The differential amplification application achieves a calculation accuracy of 98.6% for 7-bit grayscale image reconstruction, representing the lowest accuracy among all evaluated methods despite still demonstrating high operational reliability. A comparative analysis of the reconstructed images reveals three key results: the reference image generated through software simulation, the corresponding output produced by our photonic device, and a detailed difference map highlighting the discrepancies between them. The primary discrepancies are predominantly concentrated along the cranial boundaries, where edge enhancement effects amplify minor alignment variations, while the tumor region exhibits significantly lower displacement due to its structural homogeneity and consistent optical properties. This spatial distribution of errors confirms the system's stronger performance in processing continuous-tissue regions compared to high-contrast boundary transitions, providing insights for future optimization of biomedical image processing algorithms. The maintained accuracy across all functional implementations validates the architectural robustness for practical image processing applications.

Supplementary Note 9 - Scalability toward multi-bit photonic ALUs

To evaluate the potential of the proposed architecture for large-scale photonic computing, we analyze its scalability toward higher-bit ALUs, and discuss the associated challenges and feasible mitigation strategies.

A direct spatial extension of the demonstrated 2-bit architecture would require a proportional increase in the number of MRMs, leading to rapidly growing circuit complexity and footprint. To address this limitation, the proposed architecture adopts a time-domain iterative computation strategy, which enables multi-bit operation without proportional hardware scaling.

Taking the adder as an example, the input operands *A·B* are decomposed into bit sequences and encoded into time-division multiplexed (TDM) signals. At each time slot, the corresponding bits *A_i_, B_i_*, and the carry input *C_i-1_* are applied to the MRM-based logic unit, generating the sum *S_i_* and carry *C_i_*. The carry signal is partially fed back through an optical–electrical–optical (OEO) loop to serve as the input for the next time step, enabling iterative computation across multiple bit positions. Under this scheme, multi-bit addition and subtraction can be realized with approximately constant hardware resources, where scalability is primarily determined by temporal processing depth rather than spatial circuit size.

The scalability characteristics differ for arithmetic and comparison operations. For addition and subtraction, the iterative structure allows efficient reuse of the same set of BLUs. In contrast, comparator functions require an increasing number of BLU operations with bit width due to multi-bit comparison logic. For example, a 2-bit comparator requires 3 BLUs, while 4-bit and 8-bit implementations require 10 and 36 BLUs, respectively. To maintain system efficiency, the architecture performs core comparison operations in the optical domain, while the final decision logic can be completed in the electrical domain.

Scaling to larger systems introduces several practical challenges associated with cascading photonic logic units. Optical signal attenuation accumulates due to waveguide propagation loss, coupling loss, and device insertion loss, which can degrade signal integrity in deep cascaded configurations. This can be mitigated through low-loss waveguide design, optimized coupling structures, and, when necessary, the incorporation of optical amplification stages or regeneration mechanisms. Crosstalk and spectral interference may arise from dense integration of resonant devices, particularly under closely spaced wavelength channels. These effects can be reduced through careful resonance engineering, wavelength allocation, and layout-level isolation strategies.

**Supplementary Figure 10.** Schemes for bit‑width extension of arithmetic units. (a) Bit‑width extension scheme for full adders. (b) Bit‑width extension scheme for full subtractors. (c) Bit‑width extension scheme for comparators.

Signal synchronization is another critical consideration in multi-channel operation. However, this requirement is consistent with standard practices in high-speed electronic–photonic integrated systems and can be addressed through co-design of driver electronics and photonic devices, matched interconnect routing, and localized clock distribution. In addition, the use of time-domain multiplexing reduces the number of simultaneously active channels, thereby relaxing synchronization constraints in large-scale implementations.

The adoption of time-domain iterative processing introduces a fundamental trade-off between scalability and throughput. As the bit width increases, the computation becomes partially serialized, leading to a reduction in effective throughput under a fixed modulation rate. Nevertheless, given the high intrinsic speed of photonic devices, the resulting performance remains competitive for high-bandwidth applications.

Overall, the proposed architecture transforms the scaling behaviour from spatial resource growth to temporal processing overhead, providing a feasible pathway toward multi-bit photonic ALUs. Combined with advances in photonic integration and system-level co-design, this approach offers a scalable foundation for future large-scale photonic computing systems.

Supplementary Note 10 - Advantages of photonic ALUs for high-throughput computation

Photonic ALUs differ fundamentally from conventional electronic ALUs in their optimization targets. Electronic ALUs are primarily designed for high-precision sequential computation, where performance scaling is typically constrained by clock frequency, interconnect delay, and electrical power dissipation^S12, S13^. Modern electronic processors routinely support 16-bit to 32-bit precision arithmetic with high reliability, but their throughput scaling increasingly faces limitations imposed by RC delays, memory bandwidth, and data movement overhead.

In contrast, photonic ALUs are inherently suited for high-throughput parallel computation by exploiting the unique degrees of freedom available in optical systems^S14, S15^. In addition to temporal processing, optical computation can be simultaneously scaled through WDM, spatial parallelism, and high-speed electro-optic modulation. This enables multiple logic operations to be executed concurrently without relying solely on increased clock frequency or extensive hardware replication.

In this work, the proposed photonic ALU experimentally demonstrates operation at 20 Gbit/s. More importantly, prior studies have shown that substantially higher operating speeds are feasible in integrated photonic platforms. For example, in S16, while recent microring-based devices have reported modulation speeds exceeding 400 Gb/s per wavelength^S16, S17^. These advances provide a clear pathway toward significantly higher aggregate computational throughput in future photonic ALU systems.

Beyond raw throughput, photonic ALUs also offer architectural advantages in bandwidth-intensive workloads. Since optical signals propagate without capacitive charging losses and support direct parallel transmission across multiple channels, photonic architectures can significantly reduce interconnect bottlenecks and data movement overhead. As discussed in S13 and S15, these characteristics become increasingly important as communication overhead dominates modern computing systems.

Therefore, while electronic ALUs remain advantageous for high-precision general-purpose computing, photonic ALUs provide a complementary computing paradigm optimized for ultra-high-speed, high-bandwidth, and massively parallel information processing. The proposed architecture establishes a scalable foundation for extending photonic computation toward higher precision and more complex computational tasks.

**Reference**

S1.	Ghosh RR, Dhawan A. Low loss, broadband, and non-volatile ‘directed logic operations’ using phase change materials in silicon photonics. ***IEEE J Quantum Electron*** 59, 1-13 (2023).

S2.	Xia Y, et al. A multifunctional optical computing system based on directed logic and micro-ring resonator. ***Opt Laser Technol*** 175, 110696 (2024).

S3.	Ying Z, Feng C, Zhao Z, Soref R, Pan D, Chen RT. Integrated multi-operand electro-optic logic gates for optical computing. ***Appl Phys Lett*** 115, (2019).

S4.	Soref R, De Leonardis F, Ying Z, Passaro VM, Chen RT. Silicon-based group-IV OEO devices for gain, logic, and wavelength conversion. ***ACS Photonics*** 7, 800-811 (2020).

S5.	Ruhul Fatin M, Gostimirovic D, Ye WN. Reconfigurable optical logic in silicon platform. ***Sci Rep*** 14, 5950 (2024).

S6.	Ghosh RR, Dhawan A. Integrated non-volatile plasmonic switches based on phase-change-materials and their application to plasmonic logic circuits. ***Sci Rep*** 11, 18811 (2021).

S7.	Feng C, Ying Z, Zhao Z, Gu J, Pan DZ, Chen RT. Toward high‐speed and energy‐efficient computing: a WDM‐based scalable on‐chip silicon integrated optical comparator. ***Laser Photonics Rev*** 15, 2000275 (2021).

S8.	Zhang W, et al. Large-scale optical programmable logic array for two-dimensional cellular automaton. ***Adv Photonics*** 6, 056007-056007 (2024).

S9.	Ying Z, et al. Electronic-photonic arithmetic logic unit for high-speed computing. ***Nat Commun*** 11, 2154 (2020).

S10.	Zhang W, et al. Photonic logic tensor computing beyond Tbit/s per core. ***Optica*** 12, 1252-1260 (2025).

S11.	Zhang, W. *et al.* Photonic logic tensor computing beyond Tbit/s per core. *Optica* **12**, 1252-1260 (2025).

S12.	Waldrop MM. More than Moore. ***Nature*** 530, 144-148 (2016).

S13.	Tucker RS, Hinton K. Energy consumption and energy density in optical and electronic signal processing. ***IEEE Photonics J*** 3, 821-833 (2011).

S14.	Ying Z, et al. Electronic-photonic arithmetic logic unit for high-speed computing. ***Nat Commun*** 11, 2154 (2020).

S15.	McMahon PL. The physics of optical computing. ***Nat Rev Phys*** 5, 717-734 (2023).

S16.	Hu F, et al. 400‐Gbps/λ Ultrafast Silicon Microring Modulator for Scalable Optical Compute Interconnects. ***Laser Photonics Rev***, e02840 (2025).

S17.	Wang X, et al. A 400 Gbps Microring Modulator with 4.2 THz FSR Using Optimized Bezier Bend in a 300 mm CMOS Platform. ***ACS Photonics***, (2026).
